# Rising trends in the burden of migraine among children and adolescents: a comprehensive analysis from 1990 to 2021 with future predictions

**DOI:** 10.3389/fpubh.2025.1634098

**Published:** 2025-10-23

**Authors:** Qian Wang, Rong Luo, Qianhui Wen

**Affiliations:** ^1^Department of Pediatrics, West China Second University Hospital, Sichuan University, Chengdu, Sichuan, China; ^2^Key Laboratory of Birth Defects and Related Diseases of Women and Children (Sichuan University), Ministry of Education, Chengdu, Sichuan, China

**Keywords:** global burden of disease, children, adolescents, epidemiology, disability-adjusted life years, trends

## Abstract

**Background:**

Migraine is a leading neurological disorder among children and adolescents. Its high prevalence and risk of chronic progression significantly reduce quality of life and are linked to mental health issues and learning difficulties. Despite available diagnostic standards and treatments, global challenges remain, including underdiagnosis and unequal healthcare access, especially in developing countries where health systems are unprepared for rising cases. Analyzing long-term patterns and contributing factors of pediatric migraine is crucial for improving healthcare planning and prevention strategies.

**Methods:**

Using data from the Global Burden of Disease Study (GBD), this study analyzed migraine epidemiology in 5–19-year-olds across 204 countries and territories during 1990–2021. Disease burden trends were quantified through age-standardized rates (ASR) and estimated annual percentage changes (EAPC). Regions were categorized into five distinct tiers according to the Sociodemographic Index (SDI). Furthermore, a Bayesian age-period-cohort model was implemented to project disease burden trends through 2035.

**Results:**

From 1990 to 2021, global migraine cases among children and adolescents increased from 165,687,027.67 to 205,729,235.09, marking a 24.17% rise, with disability-adjusted life years (DALYs) growing by 24.38%. Low-middle SDI regions bore the heaviest disease burden, reporting 65,004,285.01 cases in 2021—triple that of high SDI regions. Females exhibited significantly higher prevalence, incidence, and DALYs than males. Trend analysis revealed declining disease burden in low-middle SDI regions but rising rates in high SDI regions. Despite projected declines in global age-standardized migraine prevalence, incidence, and DALYs by 2035, adolescents aged 15–19 are still anticipated to face disproportionately high disease burdens.

**Conclusion:**

The burden of migraine among children and adolescents exhibits significant geographical and gender heterogeneity, necessitating targeted optimization of healthcare resource allocation. Strengthening primary care diagnostic capacity, promoting standardized treatment guidelines, and prioritizing disease prevention and control in low-income regions are critical to alleviating the global burden.

## 1 Introduction

Migraine is a common primary headache disorder, ranking as the second most common neurological condition globally. According to the 2021 Global Burden of Disease (GBD) data, approximately 1.16 billion individuals worldwide were living with migraine, representing 14.7% of the global population (1). Its primary clinical manifestations include recurrent episodes of headache accompanied by nausea, vomiting, and sensitivity to light or sound (2). Migraine can cause severe disability, with functional impairment exceeding that of many other neurological disorders, and has emerged as a substantial determinant of the global burden of disease (3).

Despite established diagnostic criteria and treatments, migraine remains highly stigmatized and under-resourced. Furthermore, it is frequently misdiagnosed and systematically underestimated in clinical practice (4–6). Current global approaches to migraine management are suboptimal (7). This challenge is compounded in developing countries by healthcare systems still primarily focused on infectious diseases and inadequately adapted for chronic conditions like migraine (8).

Among children and adolescents, migraine accounts for the highest burden of neurological disability (9). Studies indicate that the global prevalence rate among this population group reaches 11% (10), with some regions reporting rates as high as 25.2% (11). Clinically, migraine significantly compromises patients’ quality of life, predominantly manifesting in impairments to physical functioning, social interactions, and psychological wellbeing (12). Patients exhibit increased school absenteeism (averaging 0.4 missed days per month) (11), academic performance deterioration, and reduced subjective wellbeing (13–17). More concerningly, school-aged patients face higher risks of academic impairment and early dropout (13, 18), while their prevalence of mood disorders is three times that of healthy peers (19).

This disorder is characterized by distinct neurobiological processes, if left untreated during critical neurodevelopmental windows, the majority of pediatric cases will persist into adulthood (20). Despite the prognostic benefits of early treatment, a substantial diagnostic conundrum persists in clinical practice. Nearly 40% of pediatric headache patients encounter phenotyping difficulties due to symptomatic overlap with other headache disorders, notably tension-type headache (21). A UK study found that only 20% of children presenting with headaches received a definitive migraine diagnosis (22), highlighting substantial gaps in current clinical recognition and management.

Migraine is a leading cause of disability-adjusted life years (DALYs) lost among children and adolescents globally. Despite its significant impact, a systematic epidemiological assessment specifically for this age group remains lacking. Existing evidence is often limited by geographic scope, small sample sizes, short follow-up periods, and methodological heterogeneity, which hinder reliable cross-national and temporal comparisons. To address this gap, this study analyzes data from the GBD 2021 study to assess the epidemiological burden and trends of migraine in children and adolescents from 1990 to 2021, and to project disease burden for the next decade.

This study aims to provide long-term trends, projections, and standardized international data on migraine in children and adolescents, thereby providing a critical evidence base for optimizing prevention efforts and targeting high-risk groups and areas.

## 2 Methods

### 2.1 Data source

The migraine data analyzed in this study were sourced from the GBD 2021 database. This database systematically integrates published scientific literature, national and regional health surveys, hospital and outpatient registry systems, and other surveillance data. Through rigorous standardization procedures and advanced statistical modeling tools (DisMod-MR 2.1), it synthesizes and analyzes these inputs to generate comprehensive epidemiological estimates. The resulting estimates cover the burden of 371 diseases and injuries across 204 countries and territories from 1990 to 2021. All data are accessible through the Global Health Data Exchange (GHDx)^1^. For the purposes of this investigation, migraine-specific data were extracted from the GBD Results Tool using the following parameters: Cause = “Migraine” (GBD Level 4 cause), Age Group = “5–9 years,” “10–14 years,” “15–19 years,” Measure = [“Prevalence,” “Incidence,” “DALYs”], Location = [“Global,” “SDI quintiles,” “GBD regions,” “Countries”], Year = [1990–2021].

### 2.2 Disease definition

Within the GBD 2021 study framework, migraine is defined as a distinct entity within the headache disorders category at the fourth level of the disease hierarchy. The case definition adheres to the diagnostic criteria of the International Classification of Headache Disorders, 3rd edition (ICHD-3). During estimation, this standardized definition was applied to source data to identify migraine cases. The source data utilized International Classification of Diseases (ICD) diagnostic codes (ICD-9: 346.0–346.9; ICD-10: G43.0-G43.919) for migraine. These ICD codes were mapped to the GBD/ICHD-3 framework for data synthesis and modeling. It is important to note that diagnostic accuracy, particularly in children and adolescents whose symptoms may differ from adults or in settings with limited specialist access, can vary and may lead to misclassification (under- or over-diagnosis) in the underlying source data.

### 2.3 Socio-demographic index

The GBD study for the year 2021 has delineated countries and territories into five stratifications of the SDI, an integrated metric for evaluating regional development based on income, educational attainment, and fertility indicators. The SDI scoring system ranges from 0 to 100, where 0 represents the lowest per capita income and educational attainment, and the highest fertility rates, while 100 signifies the highest per capita income and educational attainment, and the lowest fertility rates. The SDI categorizes nations into quintiles: low, low-middle, middle, high-middle, and high SDI.

### 2.4 Statistical analysis

To address demographic heterogeneity in age structures and population distributions across GBD datasets, age standardization was systematically implemented to mitigate structural confounding. The ASR per 100,000 population is calculated using the following formula:


$$\mathrm{ASR}=\frac{\sum_{i=1}^{N}\alpha_{i}W_{i}}{\sum_{i=1}^{N}\mathrm{Wi}}\times 10,000$$

(𝑎𝑖: the age-specific rate in i-th the age group; $W_{i}$ : the number of individuals within the same age group; N: the total number of age groups).

The EAPC is a key epidemiological metric for quantifying temporal trends in health indicators over specific time frames. The computation of the EAPC is predicated upon the natural logarithm of the rates, which are obtained from a regression model that has been fitted to the data, with time serving as the explanatory variable. This fitting procedure entails the transformation of the natural logarithms of the observed rates into a linear function. The computational formula is expressed as:


y=α+βx+ε

EAPC=100×(exp(β)−1)

(x: the year; y: the natural logarithm of the rate; α: the intercept; β: the slope; ε: the random error term)

We employed the EAPC along with its 95% confidence interval (95% CI) to analyze trends. An upward trend is indicated when the lower bound of the 95% CI is positive, whereas a negative value signifies a downward trend. Should neither of these conditions be met, it is inferred that there is no statistically significant difference in the trend.

Bayesian age-period-cohort (BAPC) modeling was implemented to project migraine burden trends among children and adolescents from 2022 to 2035. The BAPC framework postulates that adjacent age groups, time periods, and birth cohorts experience comparable temporal influences. Within this architecture, all unknown parameters were conceptualized as stochastic variables with specified prior distributions. Specifically, vague Gaussian priors were assigned to the intercept and linear trend terms. For the random effects representing non-linear variations in age, period, and birth cohort, intrinsic conditional autoregressive priors were employed to induce smoothing across adjacent groups (23). Convergence of the Markov chain Monte Carlo algorithm was assessed using the Gelman-Rubin diagnostic (potential scale reduction factor, R-hat < 1.1 for all key parameters) and visual inspection of trace plots. Projections were generated by extrapolating the fitted age, period, and cohort effects.

Statistical significance was determined using a *p*-value threshold of less than 0.05. All statistical analyses and graphical representations were conducted using R software (version 4.3.2).

## 3 Results

### 3.1 Burden of migraine at the global level

**Figure 1:**
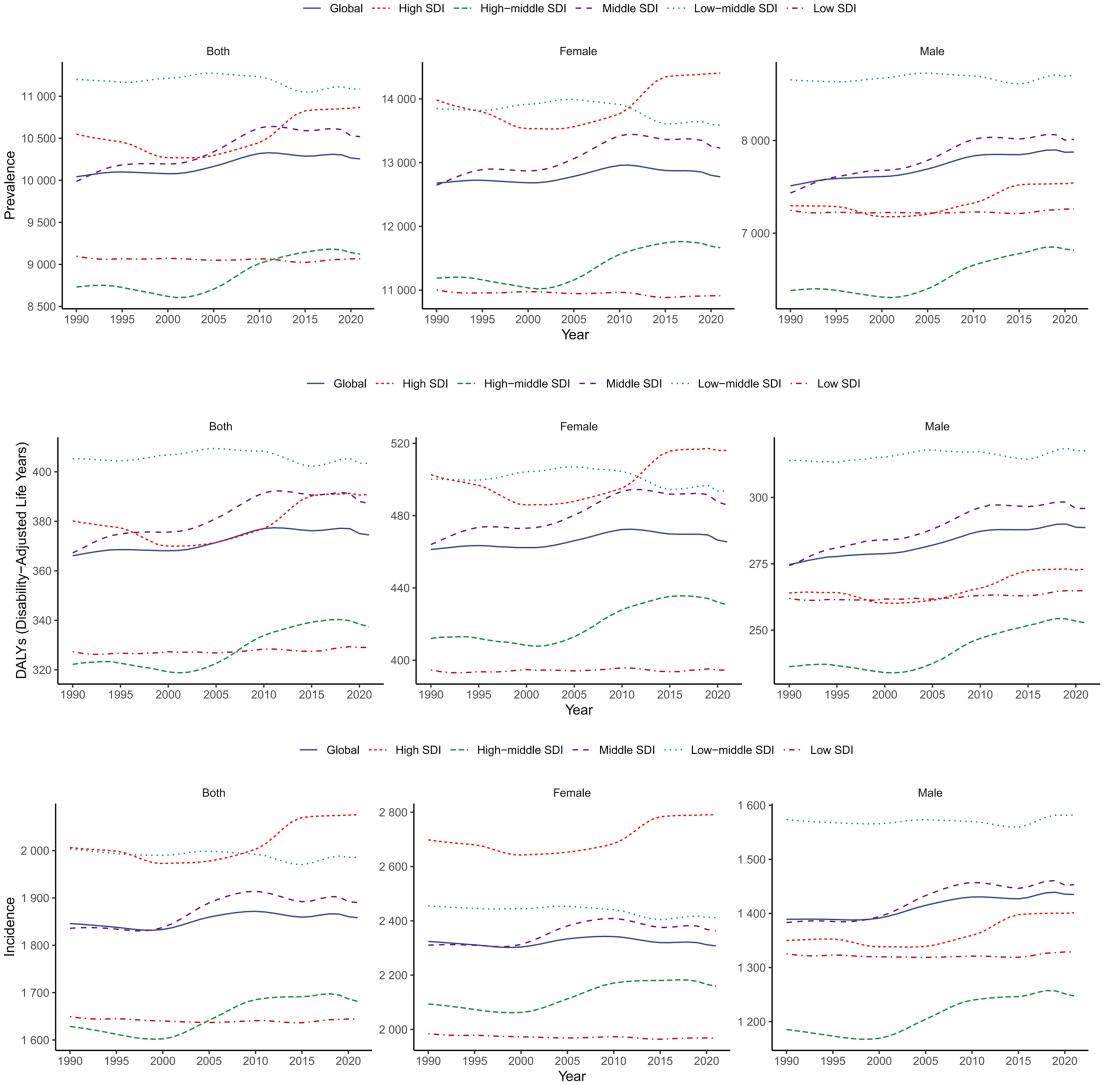
Trends in migraine prevalence, incidence and DALYs from 1990 to 2021. DALYs, disability-adjusted life-years.

Global trends demonstrated a significant increase in migraine among children and adolescents. Specifically, the number of prevalent cases climbed from 165,687,027.67 (95% UI: 122,901,783.76–215,760,523.95) in 1990 to 205,729,235.09 (95% UI: 152,945,711.93–268,680,883.20) in 2021 (+24.17%), with the age-standardized prevalence rate (ASPR) increasing from 10,042.66 (95% UI: 7,447.84–13,085.15) to 10,255.74 (95% UI: 7,620.86–13,399.98) per 100,000 (EAPC = 0.10, 95% CI: 0.08–0.11) (Supplementary Table S1; Figure 1).

Incident cases rose from 30,159,792.80 (95% UI: 20,388,195.20–41,772,246.04) to 36,794,857.75 (95% UI: 24,894,466.96–50,875,831.49) (+22%), accompanied by an elevation in the age-standardized incidence rate (ASIR) from 1,845.92 (95% UI: 1,249.97–2,553.58) to 1,858.23 (95% UI: 1,257.53–2,569.01) per 100,000 population (EAPC = 0.06, 95% CI: 0.04–0.08) (Supplementary Table S2; Figure 1).

The total DALYs increased from 6,042,775.97 (95% UI: 395,006.19–15,051,635.05) to 7,515,775.31 (95% UI: 484,365.23–18,769,692.03) (+24.38%), while the age-standardized DALY rate (ASDR) escalated from 366.11 (95% UI: 23.73–912.43) to 374.50 (95% UI: 24.02–935.71) per 100,000 population (EAPC = 0.11, 95% CI: 0.09–0.12) (Supplementary Table S3; Figure 1).

### 3.2 Burden of migraine at the SDI regional level

In the year 2021, the low-middle SDI region exhibited the most substantial migraine burden, with 65,004,285.01 (95% UI: 48,392,547.78–84,971,164.86) (Supplementary Table S1) prevalent cases, 11,368,438.93 (95% UI: 7,751,647.90–15,673,846.20) (Supplementary Table S2) incident cases, and 2,367,330.51 (95% UI: 136,646.20–5,901,590.28) (Supplementary Table S3) DALYs—the highest values across all SDI quintiles. In stark contrast, the high SDI region demonstrated the lowest burden [20,333,340.38 prevalent cases (95% UI: 14,980,993.54–26,631,114.17) (Supplementary Table S1), 3,742,835.92 incident cases (95% UI: 2,479,098.88–5,280,870.09) (Supplementary Table S2), and 732,009.88 (95% UI: 49,039.73–1,788,214.39) (Supplementary Table S3) DALYs], representing less than one-third of the low-middle SDI burden. From 1990 to 2021, despite bearing the heaviest current burden, low-middle SDI regions experienced significant declines in ASPR, ASIR, and ASDR. Conversely, high-, high-middle-, and middle-SDI regions showed sustained increases across ASPR, ASIR, and ASDR during this period. Notably, low-SDI regions displayed divergent trends: negative development in ASPR and ASIR contrasted with paradoxical growth in ASDR (Figure 1).

### 3.3 Burden of migraine at the GBD regional level

By 2021, South Asia had emerged as the region bearing the highest migraine burden among children and adolescents aged 5–19 years, with 59,338,336.40 prevalent cases (95% UI: 44,447,335.60–77,409,866.19). In contrast, Oceania recorded the lowest burden at 420,794.84 prevalent cases (95% UI: 296,538.09–573,205.39).

During 1990–2021, eight regions (Andean Latin America, Central Latin America, East Asia, Eastern Sub-Saharan Africa, North Africa and Middle East, Southern Latin America, Tropical Latin America, Western Europe) demonstrated significant increases in ASPR, ASIR, and ASDR attributable to migraine (Supplementary Tables S1–S3). Tropical Latin America showed the most marked elevation, with EAPC values of 0.37 (95% UI: 0.20–0.53) for ASPR, 0.32 (95% UI: 0.18–0.46) for ASIR, and 0.39 (95% UI: 0.22–0.56) for ASDR. Conversely, eight other regions (Caribbean, Central Asia, Central Europe, High-income Asia Pacific, South Asia, Southeast Asia, Southern Sub-Saharan Africa, Western Sub-Saharan Africa) exhibited significant declines across all three standardized metrics (Supplementary Tables S1–S3). The most pronounced decreases occurred in High-income Asia Pacific, showing EAPC values of −0.07 (95% UI: −0.08 to −0.06) for ASPR, −0.07 (95% UI: −0.09 to −0.06) for ASIR, and −0.06 (95% UI: −0.07 to −0.05) for ASDR.

**Figure 2:**
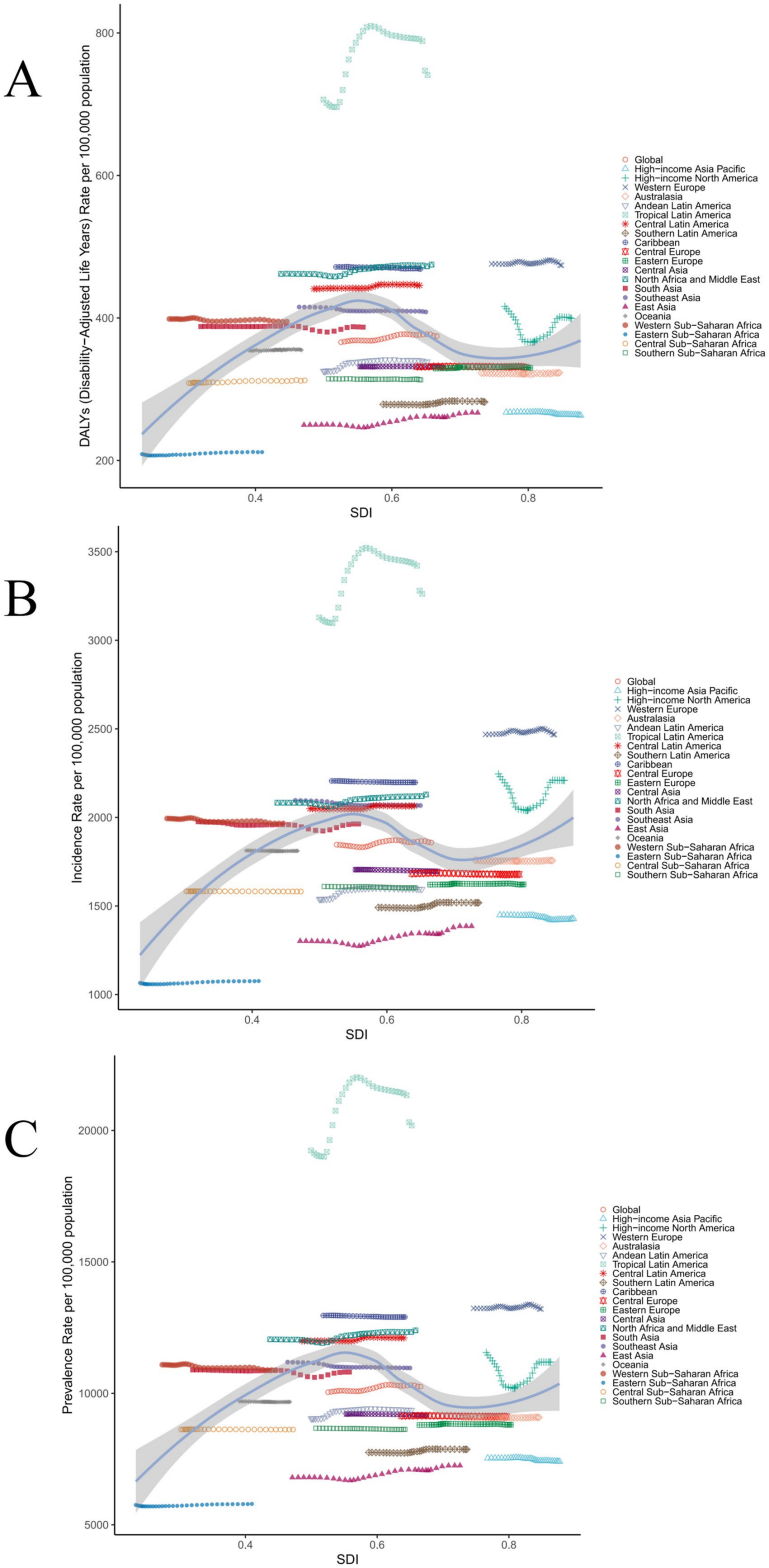
The associations between the SDI and migraine across 21 GBD regions. **(A)** SDI and ASDR; **(B)** SDI and ASIR; **(C)** SDI and ASPR. SDI, sociodemographic index; GBD, Global Burden of Disease; ASPR, age-standardized prevalence rate; ASIR, age-standardized incidence rate; ASDR, Age-standardized DALY rate.

Among the 21 GBD regions, it is observed that Southeast Asia, Western Europe, High-income North America, the Caribbean, Central Latin America, Tropical Latin America, North Africa and Middle East, South Asia, and Western Sub-Saharan Africa are the nine regions wherein the metrics for ASPR, ASDR, and ASIR all surpass the global mean benchmarks in 2021 (Figure 2).

### 3.4 Burden of migraine at the countries level

**Figure 3:**
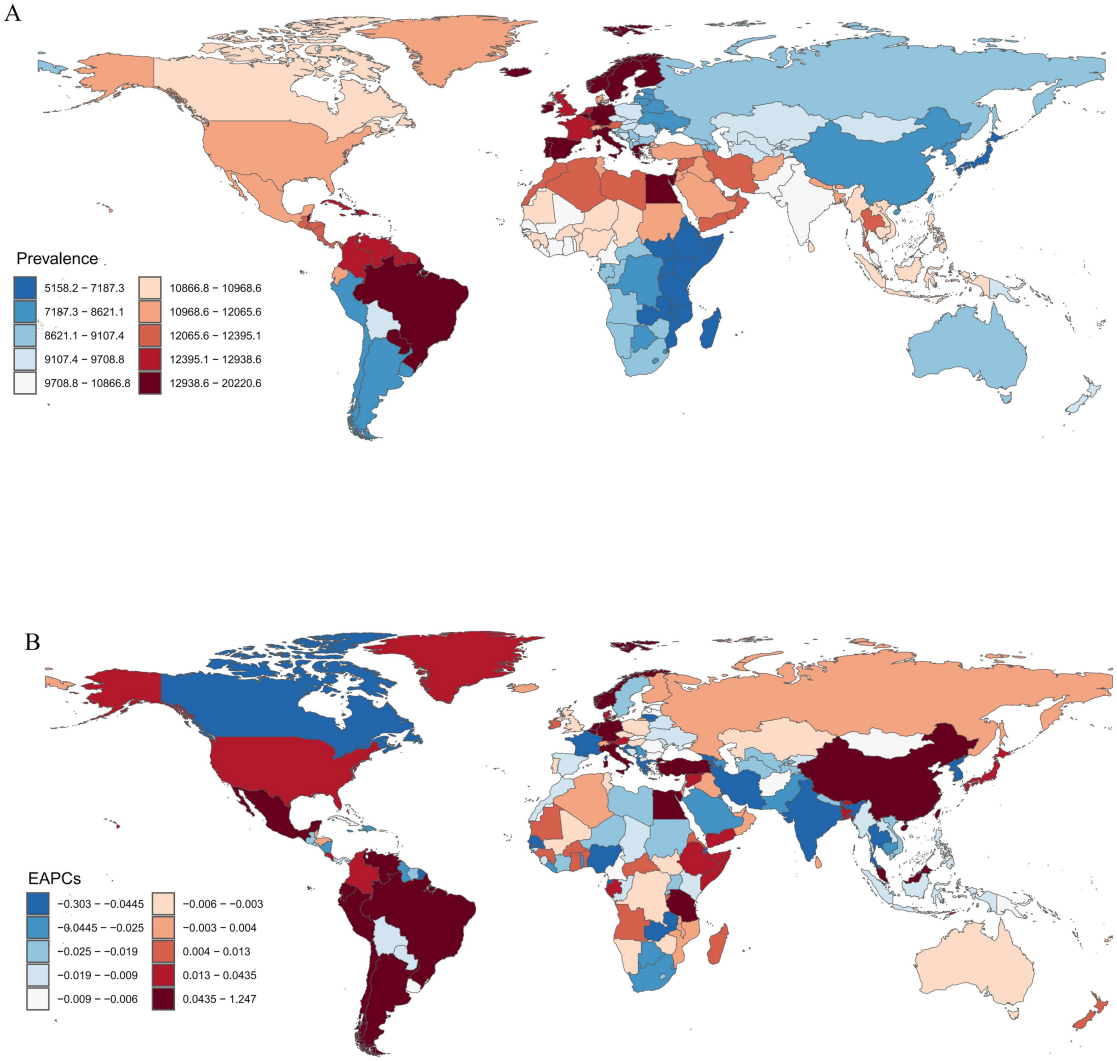
Migraine burden in 204 countries and territories. **(A)** The ASPR in 2021; **(B)** EAPC in ASPR from 1990 to 2021. ASPR, age-standardized prevalence rate; EAPC, estimated annual percentage change.

From 1990 to 2021, migraine prevalence among children and adolescents aged 5–19 years varied substantially across countries. In the year 2021, Brazil documented the highest ASPR for migraine, registering 20,220.58 cases per 100,000 population. Ethiopia reported the lowest ASPR, with a prevalence of 5,158.23 cases per 100,000 population, highlighting a marked discrepancy in the burden of migraine at the global level (Figure 3A). Norway showed the steepest prevalence increase (EAPC = 1.2, 95% CI: 0.99–1.51), contrasting with Thailand’s most pronounced decline (EAPC = −0.3, 95% CI: −0.39 to −0.21) (Figure 3B; Supplementary Table S4).

For incidence, Brazil recorded the highest rate (3,267.32 per 100,000 population) in 2021, compared to Ethiopia’s lowest rate (980.38 per 100,000 population) (Supplementary Figure S1A; Supplementary Table S5). Norway also demonstrated the fastest incidence growth (EAPC = 1.03; 95% CI: 0.82–1.24), while South Korea showed the sharpest reduction (EAPC = −0.25; 95% CI: −0.31 to −0.20) (Supplementary Figure S1B; Supplementary Table S5).

Regarding DALYs, Brazil had the highest age-standardized rate (741.91 per 100,000 population), with Ethiopia again exhibiting the lowest burden (187.17 per 100,000 population) (Supplementary Figure S2A; Supplementary Table S6). Norway ranked first in the increase of ASDR (EAPC = 1.23; 95% CI: 0.97–1.49) (Supplementary Figure S2B; Supplementary Table S6).

### 3.5 Age and sex patterns of migraine

**Figure 4:**
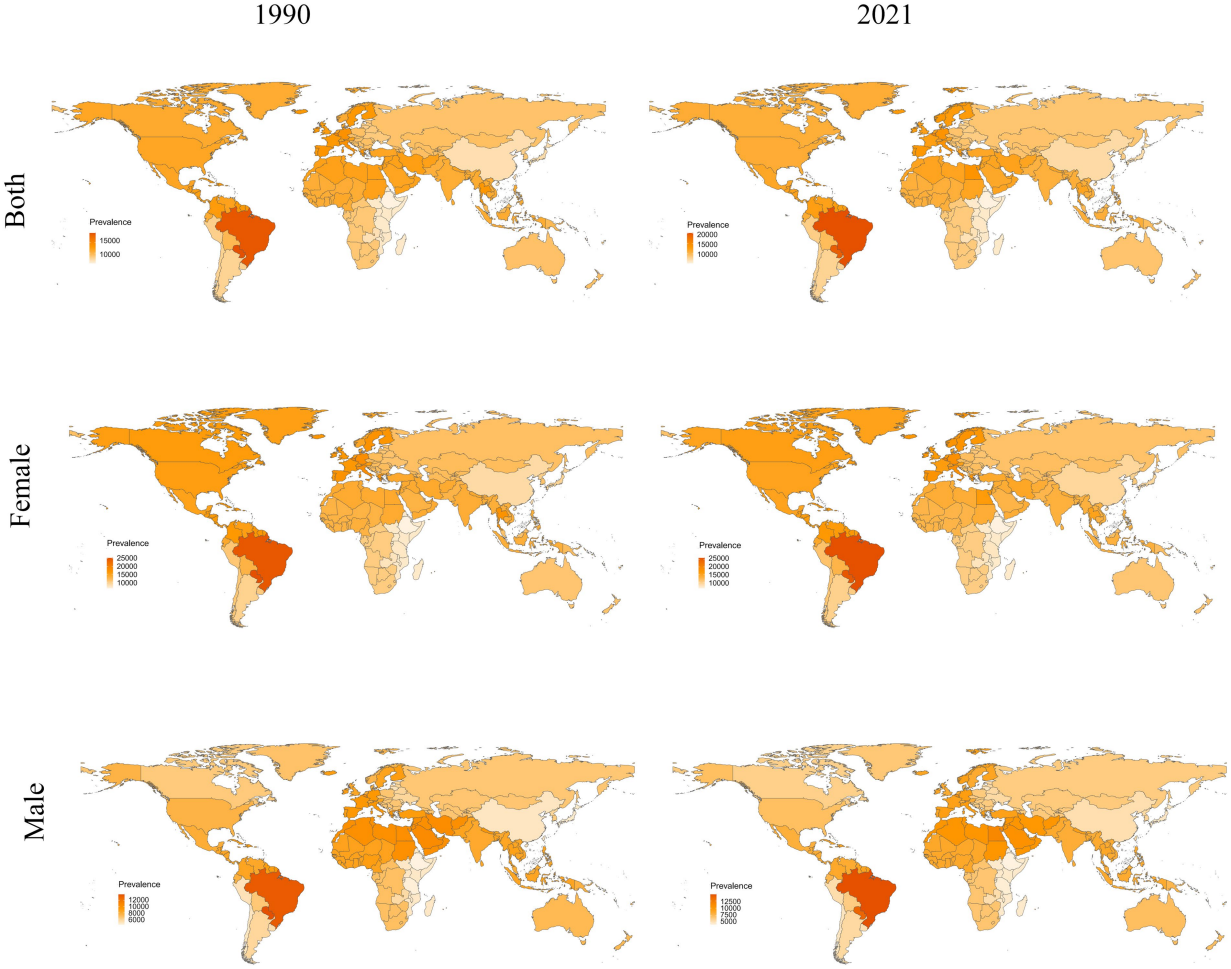
Comparison of the global disease burden of migraine prevalence in 5–19 across 204 countries and territories, 1990–2021.

From 1990 to 2021, a persistent gender disparity existed in ASPR of migraine among children and adolescents aged 5–19 years, with females consistently demonstrating higher rates than males (Figure 4). Similar disparities were observed in ASIR and ASDR, where females maintained elevated ASIR and ASDR levels throughout the study period (Supplementary Figures S3, S4). These findings reveal a gender-specific disease burden pattern in pediatric migraine epidemiology.

### 3.6 Decomposition analysis of migraine

**Figure 5:**
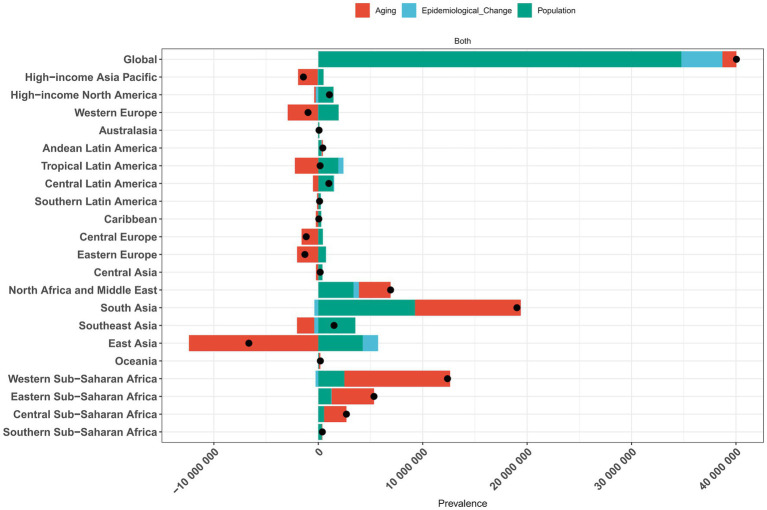
Decomposition analysis of changes in migraine prevalence across 21 GBD regions, 1990–2021. SDI, sociodemographic index; GBD, Global Burden of Disease.

Our decomposition analysis quantified the contributions of population aging, growth, and structural shifts to the epidemiological trajectory of migraine prevalence, incidence and DALYs across 21 GBD study regions. Population growth was the primary contributor to the global increase in migraine prevalence among 5-19-year-olds from 1990 to 2021 (Figure 5). Conversely, the reduced burden observed in the High-income Asia Pacific, Western Europe, Central Europe, Eastern Europe, and East Asia regions is primarily due to the effect of aging, which exerted a downward trend despite the countervailing forces of population growth and epidemiological transitions. During this period, South Asia experienced a marked rise in the total incidence, prevalence, and DALYs for migraine, largely attributable to population growth (Figure 5; Supplementary Figures S5, S6).

### 3.7 Future burden of migraine

**Figure 6:**
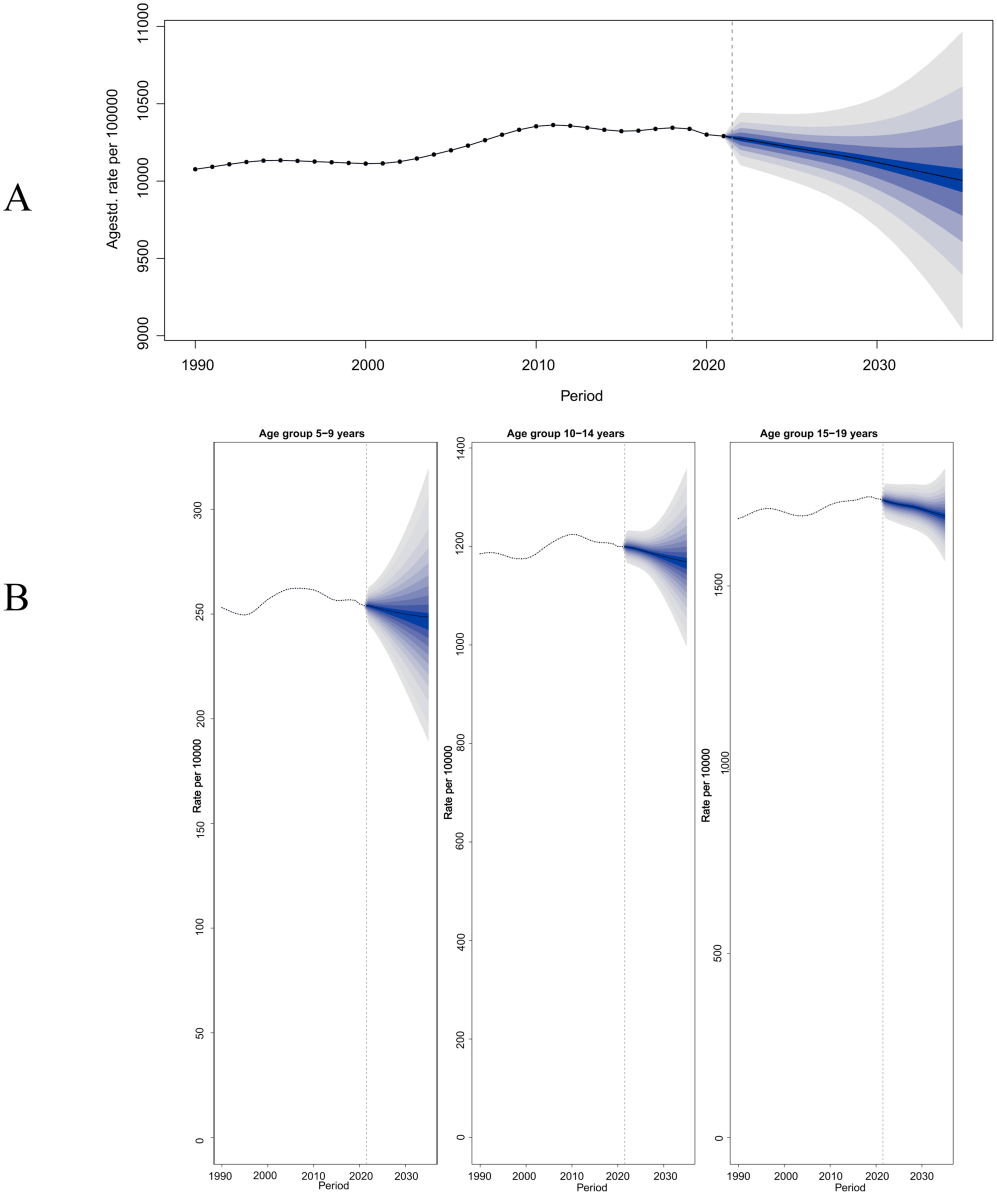
Future forecasts of migraine prevalence from 2021 to 2035. **(A)** GBD-based projections of 5–19 year-olds; **(B)** GBD-based projections at all age stages. GBD, Global Burden of Disease.

We predict that by 2035, the global prevalence trend of migraine among children and adolescents aged 5–19 years will decline (Figure 6), with an estimated 10,004.20 cases per 100,000 population (95% UI: 8,862.31–11,146.09). Among the three age groups of 5–9 years, 10–14 years, and 15–19 years, the 15–19 years age group is expected to maintain the highest prevalence by 2035, with a projected 16,902.06 cases per 100,000 population globally (95% UI: 15,656.98–18,147.16). The predicted trend for the incidence rate in 2035 also shows a considerable decline, with an estimated 1,785.62 cases per 100,000 population (95% UI: 1,504.60–2,066.64). The 10–14 years age group is expected to have the highest incidence rate among the three age groups in 2035, with a projected 2,276.99 cases per 100,000 population (95% UI: 1,919.84–2,634.14) (Supplementary Figure S7). Regarding the ASDR, a slight decrease is also anticipated by 2035, with an estimated 366.83 cases per 100,000 population (95% UI: 325.29–408.38). The 15–19 years age group is projected to maintain the highest DALY rate by 2035, with a global estimate of 632.27 cases per 100,000 population (95% UI: 561.02–703.52) (Supplementary Figure S8). Overall, by 2035, the ASPR, ASIR, and ASDR for migraine among children and adolescents aged 5–19 years are all predicted to be lower than those in 2021.

## 4 Discussion

Utilizing GBD 2021 data, this study examines the evolving global burden of migraine among individuals aged 5–19 years from 1990 to 2021. We observed substantial increases in prevalence, incidence, and DALYs during this period. Additionally, we project migraine burden for this demographic over the next 15 years, offering evidence to guide healthcare policy and resource allocation.

Our epidemiological analysis revealed a significant increase in the global migraine burden among individuals aged 5–19 years. This upward trend may be associated with multiple contributing factors, including shifts in daily routines, disrupted sleep patterns, increased digital device usage, and psychosocial stressors (24). Environmental factors—such as exposure to intense artificial light, noise pollution, elevated temperatures, and airborne irritants—are additional factors potentially linked to migraine burden (24). In specific regions, notably Tropical Latin America and East Asia, migraine burden increased more markedly. Rapid industrialization and urbanization have amplified individual stress, altered lifestyles, and degraded ambient air quality. Additionally, inadequate healthcare resources in these regions may contribute to variations in standardized diagnosis and treatment (25, 26). Inconsistent implementation of medical standards and suboptimal medication management further compromise therapeutic efficacy (27). Collectively, these factors are linked to migraine’s population health burden.

Consistent with the analysis of GBD data from other age groups, a significant discrepancy in the burden of migraine between females and males is observed, with females exhibiting a notably higher prevalence of migraine compared to males (28). Studies have indicated that among adolescents, the prevalence of migraine in girls increases from 4.6 to 9.8% by the age of 17, whereas the prevalence in boys remains relatively stable at around 4% (29). The overall odds ratio for susceptibility to migraine in adolescent females is 1.53 (14). Simultaneously, females with migraine present with a more profound clinical phenotype, characterized by more intense migraine symptoms, a higher level of disability related to migraine, and a greater burden of comorbidities, which intensifies with advancing age (30). This outcome may be correlated with the endocrine profile, specifically the estradiol levels, which are pivotal in the increased incidence of migraine among the female population (31, 32).

We found that low-middle SDI regions bore the heaviest burden of migraine, while the burden in high SDI regions was relatively lightest. The reasons for this disparity are likely multifactorial and complex. Factors potentially associated with lower burden in high-SDI settings include more developed healthcare infrastructure and greater disease awareness, although the current findings can only explain part of the observed variations. Future population-based validation studies in underrepresented regions are still needed to provide a more objective assessment of the differential burden of the disease and to elucidate the complex interplay of factors contributing to these regional differences.

Our findings demonstrate geographic variations in pediatric migraine incidence across nations, potentially linked to multidimensional interactions among biological, psychosocial, and cultural factors (33). However, critical limitations in data quality must be acknowledged when interpreting these disparities, particularly in low-resource settings. For instance, in Brazil, despite nationwide deployment of community health programs, the persistently high recorded migraine prevalence is observed alongside relatively advanced diagnostic capacity compared to neighboring regions (34). This suggests the observed prevalence may encompass both substantial true disease burden and potentially higher levels of case ascertainment. Conversely, Ethiopia’s lower recorded incidence coincides with constrained healthcare access, a known contributor to underdiagnosis, suggesting the true risk may be higher than recorded (35).

The rapid rise reported in Norway and Singapore warrants caution. While it could indicate true epidemiological growth, it may also be associated with factors characteristic of these high-income settings—such as enhanced disease awareness, greater availability of specialists, or systematic headache registries potentially enhancing detection. These factors might influence the magnitude of observed trends. Additionally, studies indicate an association between regional haze in Southeast Asia and elevated migraine incidence in Singapore (36). In contrast, South Korea’s decline in observed migraine prevalence was observed concurrently with healthcare system optimization, notably nationwide education programs designed to improve recognition and promote the dissemination of standardized, guideline-based treatments (37). Cross-regional comparisons must still account for confounding factors like variations in healthcare access, inconsistent diagnostic criteria application, and cultural influences on symptom reporting.

Current evidence supports initiating migraine management in primary care. European data indicate that adequately resourced primary healthcare systems address most patient needs (38). Given insufficient disease awareness, equipping general practitioners with robust diagnostic skills is essential, as even foundational training significantly improves clinical capability (39). While primary care typically suffices for most cases, complex presentations necessitate specialist referral. However, specialist access remains limited even in high-income settings (40, 41). Therefore, joint strengthening of both care tiers is critical for comprehensive management. Stepped-care models must prioritize standardized medication protocols (preventing medication-overuse headache), evidence-based non-pharmacological therapies, and identification of comorbidities and risk factors in special populations (42).

Our epidemiological projections indicate a predicted decline in the migraine incidence burden among children and adolescents aged 5–19 years by 2035. However, the disease burden of migraine within this population is anticipated to remain at a substantial level. This persistent burden underscores the imperative of establishing an international multi-center collaborative mechanism. Such a mechanism is essential to validate the cost-effectiveness of diverse intervention strategies, particularly assessing the feasibility of implementing tiered healthcare systems in resource-limited settings (43). Concurrently, it is imperative to strengthen the standardization of global disease surveillance systems to mitigate data bias arising from discrepancies in diagnostic criteria and reporting frameworks.

While our study provides critical insights into pediatric migraine epidemiology, several limitations should be acknowledged. A key limitation arises from the diagnostic framework’s reliance on Level IV migraine classification rather than broader Level III headache disorders, an approach prone to diagnostic inaccuracies including misclassification and underascertainment that may systematically deflate burden estimates. Further constraints stem from heterogeneous data quality across nations, where suboptimal disease surveillance infrastructure in certain regions introduces non-random missingness patterns that complicate epidemiological extrapolation. Of particular concern are cross-cultural disparities in symptom recognition and healthcare-seeking behaviors, factors that may distort comparative analyses through differential case ascertainment.

Nonetheless, this study provides critical insights into the evolving burden of pediatric migraine, paving the way for targeted policies and clinical improvements. To translate these findings into action, we recommend a dual focus: standardizing diagnostic criteria globally to ensure consistent care, and advancing integrated, multidisciplinary treatment models for personalized precision medicine. These parallel advancements are essential to alleviate patient suffering and reduce the societal burden of migraine.

## 5 Conclusion

Global pediatric migraine burden exhibits marked geographical and gender disparities, with adolescents in low-resource settings and females disproportionately affected. While ASRs are projected to decline, persistently high risks among older adolescents and widening gaps in urbanizing regions underscore the urgency to integrate migraine management into health system reforms. Addressing systemic inequities requires prioritized diagnostic capacity in underserved areas, gender-responsive interventions, and collaborative strategies to align care with local epidemiological trends, ultimately reducing long-term disability and safeguarding youth development.

## Data Availability

The datasets presented in this study can be found in online repositories. The names of the repository/repositories and accession number(s) can be found in the article/Supplementary material.

## Footnotes

^1^ https://ghdx.healthdata.org/gbd-2021/sources

## Supplementary material

Supplementary Figure S1 Migraine burden in 204 countries and territories. **(A)** The ASIR in 2021; **(B)** EAPC in ASIR from 1990 to 2021.

Supplementary Figure S2 Migraine burden in 204 countries and territories. **(A)** The ASDR in 2021; **(B)** EAPC in ASDR from 1990 to 2021.

Supplementary Figure S3 Comparison of the global disease burden of migraine incidence in 5-19 across 204 countries and territories, 1990-2021.

Supplementary Figure S4 Comparison of the global disease burden of migraine DALYs in 5-19 across 204 countries and territories, 1990-2021.

Supplementary Figure S5 Decomposition analysis of changes in migraine incidence across 21 GBD regions, 1990– 2021.

Supplementary Figure S6 Decomposition analysis of changes in migraine DALYs across 21 GBD regions, 1990– 2021.

Supplementary Figure S7 Future forecasts of migraine incidence from 2021 to 2035. **(A)** GBD-based projections of 5-19 year-olds; **(B)** GBD-based projections at all age stages.

Supplementary Figure S8 Future forecasts of migraine DALYs from 2021 to 2035. **(A)** GBD-based projections of 5-19 year-olds; **(B)** GBD-based projections at all age stages.

Supplementary table S1 Prevalence of migraine in children and adolescents aged 5 to 19 years in 1990 and 2021 at global and regional levels, with EAPCs from 1990 to 2021.

Supplementary table S2 Incidence of migraine in children and adolescents aged 5 to 19 years in 1990 and 2021 at global and regional levels, with EAPCs from 1990 to 2021.

Supplementary table S3 DALYs of migraine in children and adolescents aged 5 to 19 years in 1990 and 2021 at global and regional levels, with EAPCs from 1990 to 2021.

Supplementary table S4 Prevalence of migraine in children and adolescents aged 5 to 19 years in 1990 and 2021 across 204 countries and territories, with EAPCs from 1990 to 2021.

Supplementary table S5 Incidence of migraine in children and adolescents aged 5 to 19 years in 1990 and 2021 across 204 countries and territories, with EAPCs from 1990 to 2021.

Supplementary table S6 DALYs of migraine in children and adolescents aged 5 to 19 years in 1990 and 2021 across 204 countries and territories, with EAPCs from 1990 to 2021.
