# Supplementary figures and images for: Rising trends in the burden of migraine among children and adolescents: a comprehensive analysis from 1990 to 2021 with future predictions
Source: Front Public Health. 2025 Oct 23;13:1634098. doi: 10.3389/fpubh.2025.1634098 (PMC12589008; doi:10.3389/fpubh.2025.1634098)

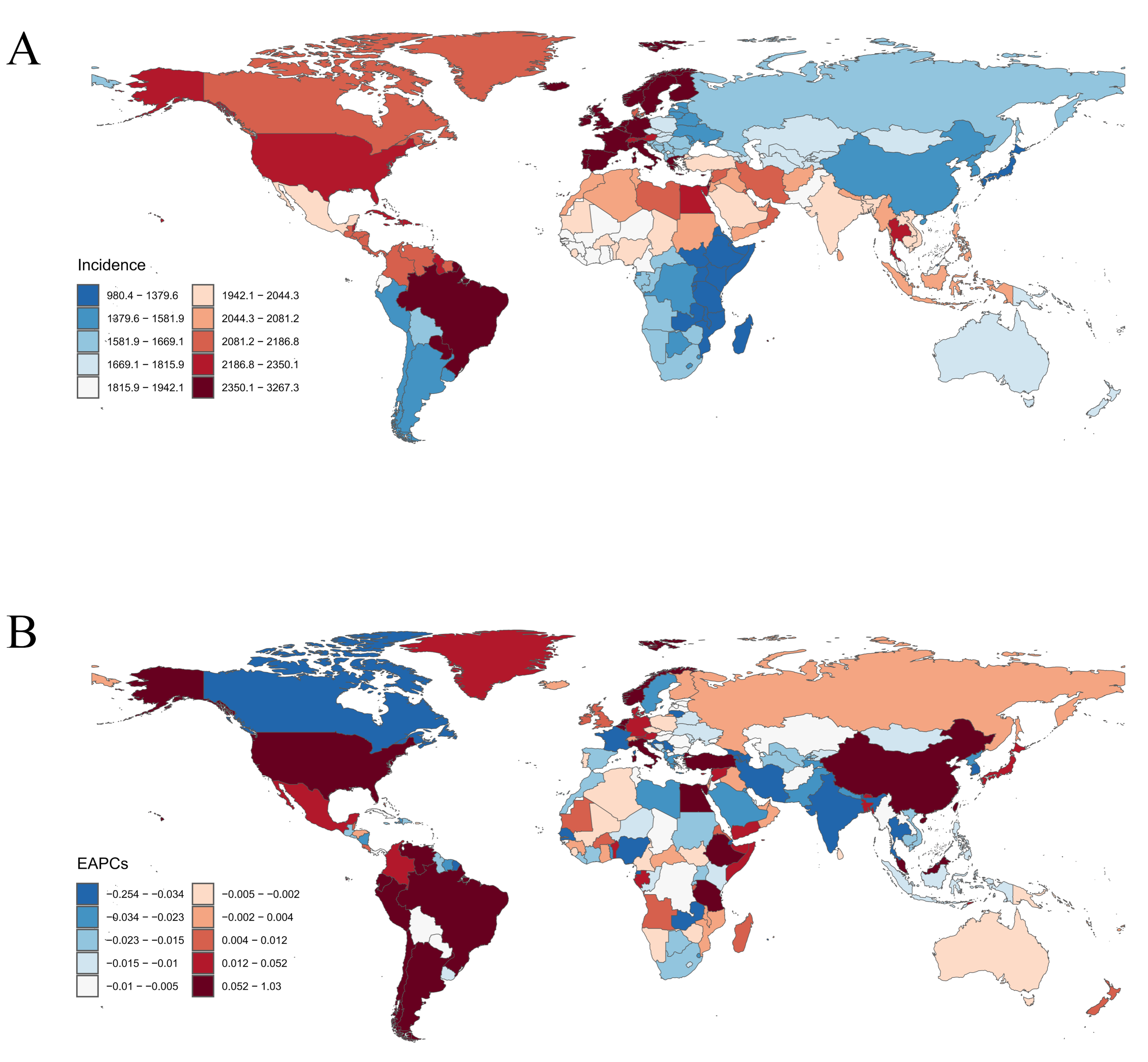

Supplement: Supplementary Figure S1 — Migraine burden in 204 countries and territories. (A) The ASIR in 2021; (B) EAPC in ASIR from 1990 to 2021. [file Image_1.jpeg]

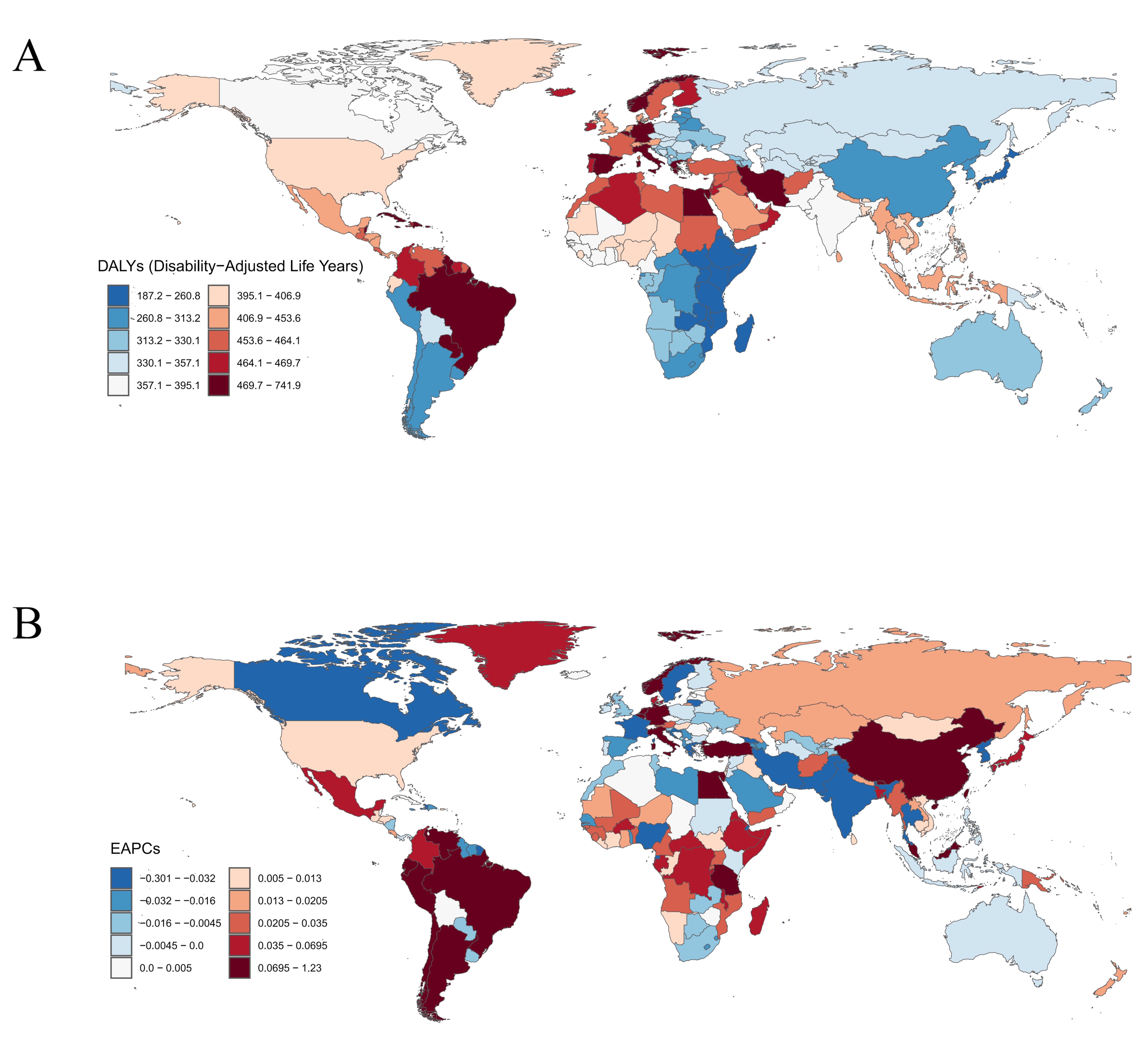

Supplement: Supplementary Figure S2 — Migraine burden in 204 countries and territories. (A) The ASDR in 2021; (B) EAPC in ASDR from 1990 to 2021. [file Image_2.jpeg]

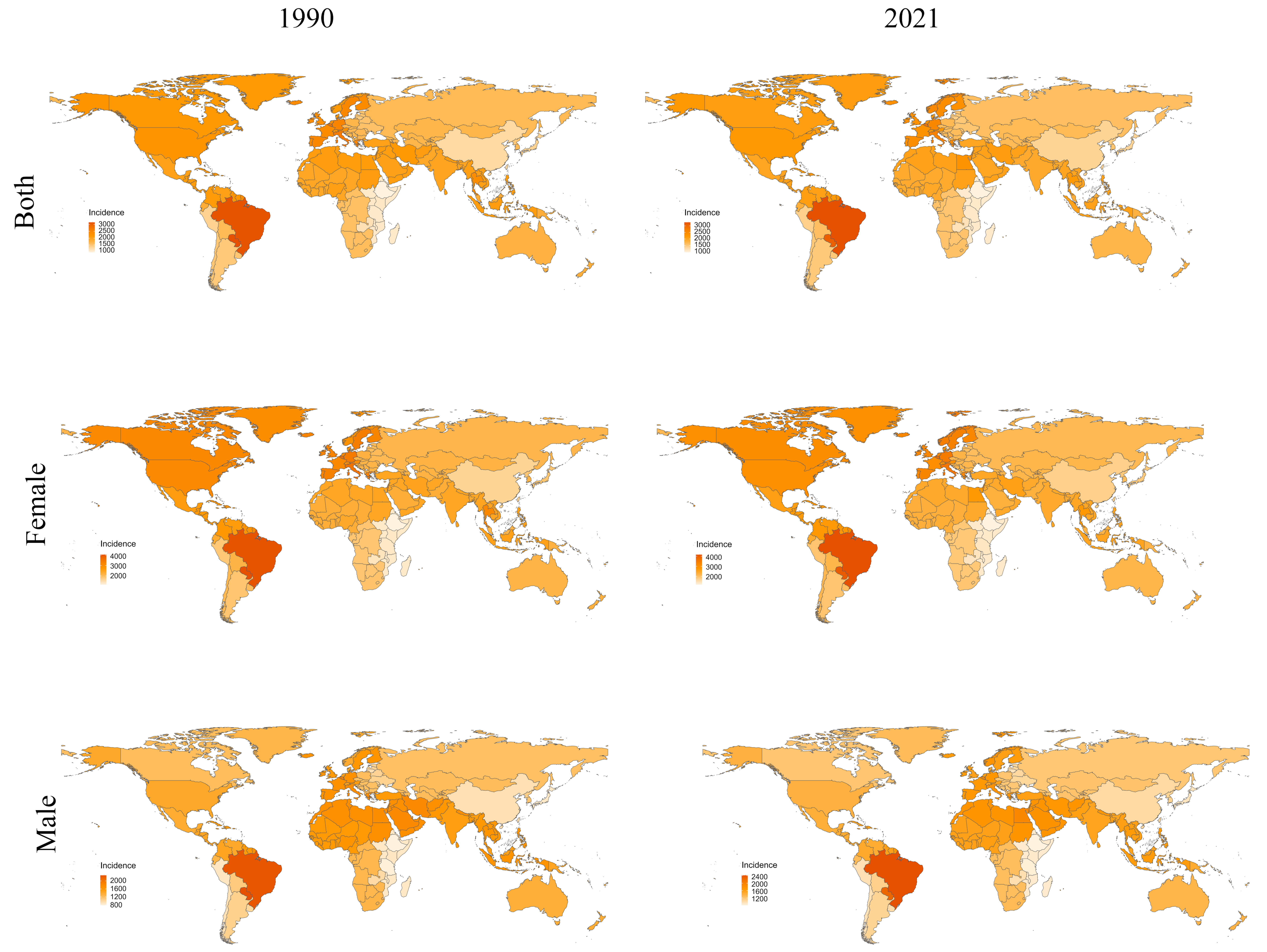

Supplement: Supplementary Figure S3 — Comparison of the global disease burden of migraine incidence in 5-19 across 204 countries and territories, 1990-2021. [file Image_3.jpeg]

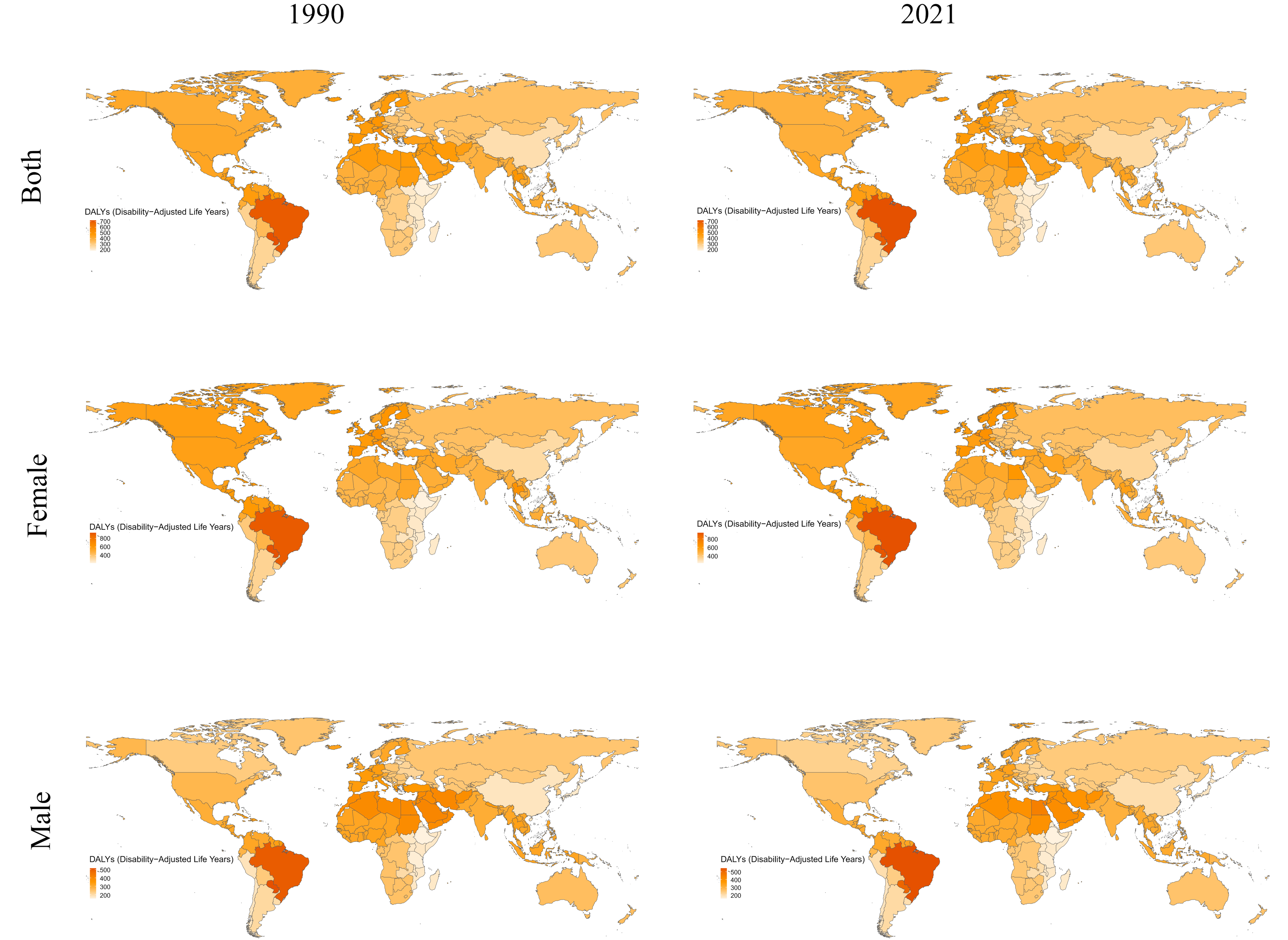

Supplement: Supplementary Figure S4 — Comparison of the global disease burden of migraine DALYs in 5-19 across 204 countries and territories, 1990-2021. [file Image_4.jpeg]

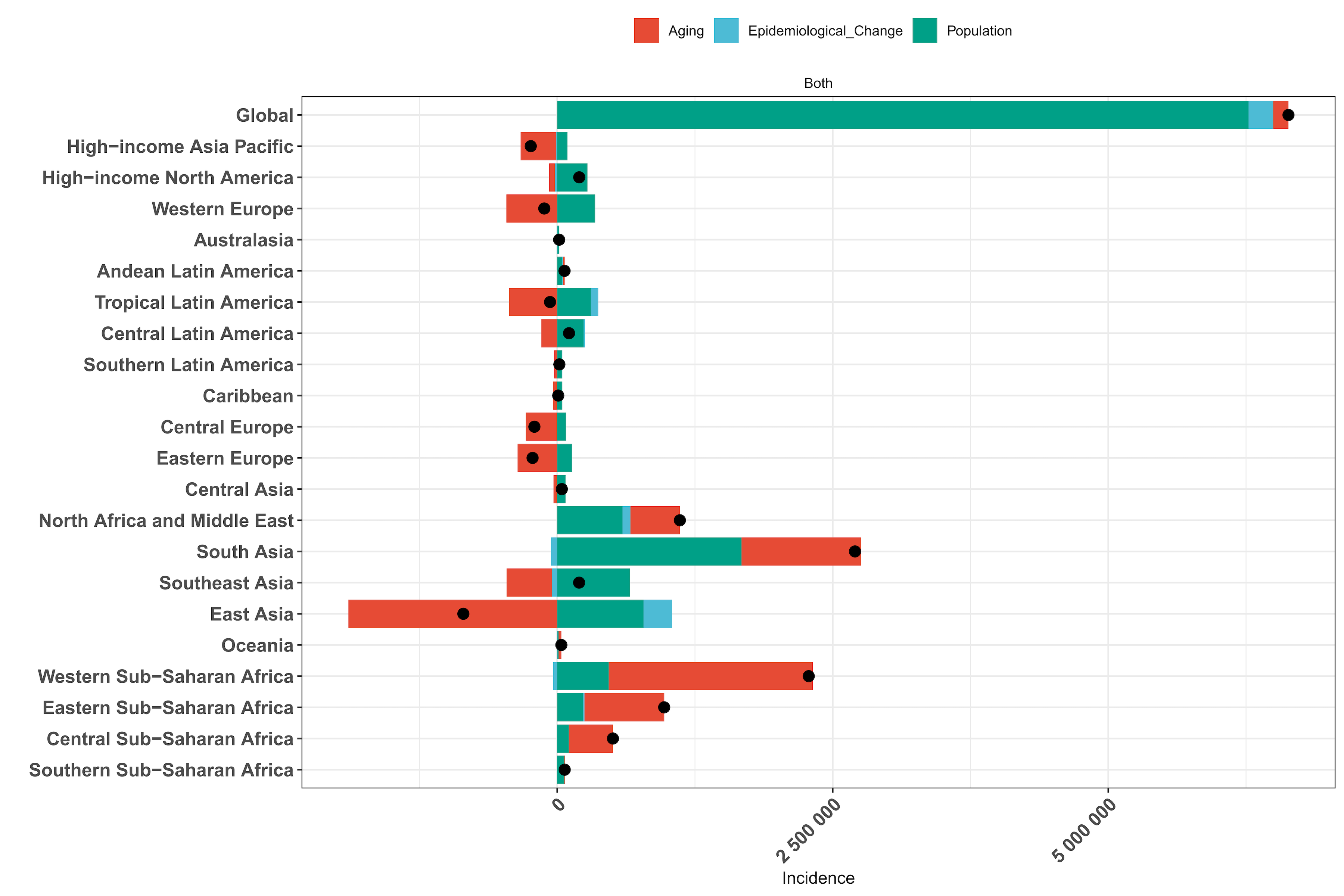

Supplement: Supplementary Figure S5 — Decomposition analysis of changes in migraine incidence across 21 GBD regions, 1990– 2021. [file Image_5.jpeg]

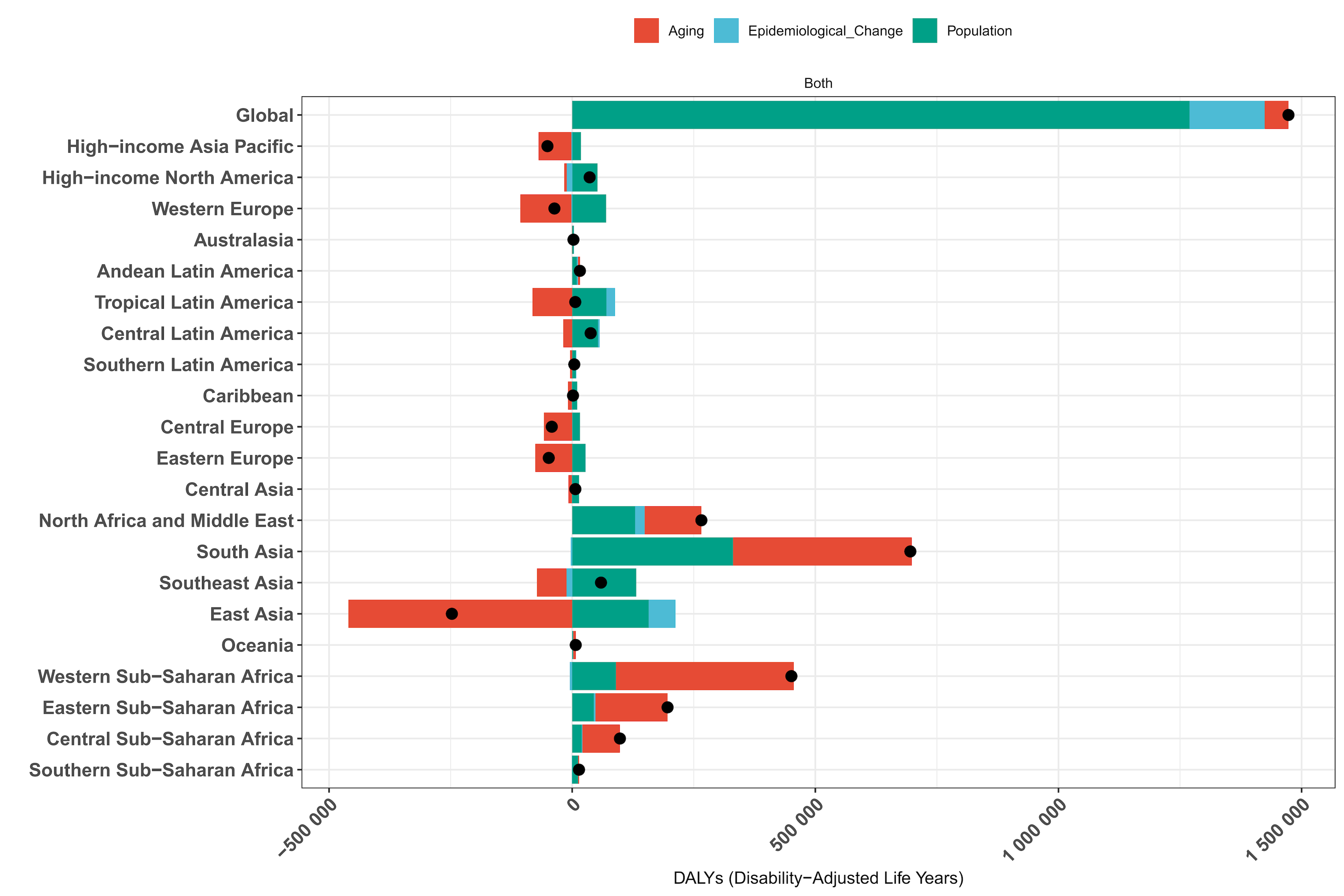

Supplement: Supplementary Figure S6 — Decomposition analysis of changes in migraine DALYs across 21 GBD regions, 1990– 2021. [file Image_6.jpeg]
