# Supplementary material for: Rising trends in the burden of migraine among children and adolescents: a comprehensive analysis from 1990 to 2021 with future predictions
Source: Front Public Health. 2025 Oct 23;13:1634098. doi: 10.3389/fpubh.2025.1634098 (PMC12589008; doi:10.3389/fpubh.2025.1634098)
Supplement: Supplementary table S1 — Prevalence of migraine in children and adolescents aged 5 to 19 years in 1990 and 2021 at global and regional levels, with EAPCs from 1990 to 2021. [file Table_1.docx]

Table S1. Prevalence of migraine in children and adolescents aged 5 to 19 years in 1990 and 2021 at global and regional levels, with EAPCs from 1990 to 2021

| Location | Prevalence | | | | |
| --- | --- | --- | --- | --- | --- |
|  | Number of cases (95% UI) | | ASR per 100,000 population (95% UI) | | EAPC (95% CI) |
|  | 1990 | 2021 | 1990 | 2021 | 1990-2021 |
| Global | 165687027.67(122901783.76,215760523.95) | 205729235.09(152945711.93,268680883.20) | 10042.66(7447.84,13085.15) | 10255.74(7620.86,13399.98) | 0.10(0.08,0.11) |
| High SDI | 20989284.48(15379158.02,27306405.04) | 20333340.38(14980993.54,26631114.17) | 10550.51(7725.02,13763.44) | 10868.58(7999.30,14259.27) | 0.15(0.09,0.21) |
| High-middle SDI | 25442258.98(18824058.34,33205630.83) | 21424548.47(15756493.27,28033166.15) | 8731.52(6451.44,11423.78) | 9122.65(6707.77,11938.76) | 0.22(0.17,0.27) |
| Middle SDI | 57766062.62(42996541.89,74989571.41) | 61363187.55(45686747.77,79772520.57) | 9987.65(7428.99,12979.45) | 10520.21(7828.39,13683.67) | 0.20(0.17,0.24) |
| Low-middle SDI | 45182842.94(33317415.54,59258548.92) | 65004285.01(48392547.78,84971164.86) | 11200.65(8267.89,14669.07) | 11085.09(8246.48,14501.39) | -0.03(-0.06,-0.01) |
| Low SDI | 16150739.10(11739089.35,21465258.21) | 37444401.85(27312720.78,49847283.50) | 9097.90(6628.60,12059.65) | 9066.89(6616.38,12061.40) | -0.01(-0.02,-0.00) |
| Andean Latin America | 1230120.44(897368.17,1613477.75) | 1661871.23(1188623.67,2250254.17) | 9038.72(6597.36,11850.80) | 9361.67(6689.10,12687.90) | 0.14(0.10,0.17) |
| Australasia | 458538.58(323621.30,615803.82) | 531169.09(374058.37,715612.03) | 9099.73(6405.38,12264.86) | 9086.54(6394.73,12248.57) | -0.00(-0.00,0.00) |
| Caribbean | 1460852.74(1034798.08,1971305.65) | 1508612.33(1068136.28,2035961.32) | 12960.39(9158.69,17527.92) | 12902.70(9118.44,17443.32) | -0.02(-0.02,-0.02) |
| Central Asia | 1993838.41(1397876.74,2702579.98) | 2174702.31(1521844.84,2953124.87) | 9211.77(6463.97,12476.31) | 9167.72(6429.65,12416.44) | -0.02(-0.02,-0.02) |
| Central Europe | 2861520.62(2055198.71,3794408.52) | 1706806.90(1228662.43,2262496.17) | 9141.06(6556.99,12135.66) | 9117.13(6554.02,12100.66) | -0.01(-0.02,-0.01) |
| Central Latin America | 7169563.13(5283657.75,9368112.34) | 8178450.67(6010091.17,10762121.59) | 11995.16(8839.97,15672.73) | 12109.81(8886.92,15958.80) | 0.05(0.04,0.06) |
| Central Sub-Saharan Africa | 1684478.30(1167738.89,2292651.54) | 4377370.35(3036838.30,5954280.62) | 8625.26(5997.96,11702.76) | 8622.30(5995.96,11699.18) | -0.00(-0.00,-0.00) |
| East Asia | 25448541.45(18864555.87,33627032.64) | 18798377.52(13787878.35,24813474.27) | 6798.65(5029.67,9015.21) | 7252.15(5320.48,9566.26) | 0.26(0.21,0.30) |
| Eastern Europe | 4440002.40(3266525.27,5717545.63) | 3156228.16(2322147.28,4071902.66) | 8796.04(6469.58,11334.37) | 8808.19(6481.99,11354.00) | 0.00(-0.00,0.01) |
| Eastern Sub-Saharan Africa | 4075235.35(2917163.01,5462099.65) | 9407225.47(6744489.29,12590667.83) | 5754.02(4128.49,7688.40) | 5794.00(4156.02,7749.73) | 0.05(0.04,0.07) |
| High-income Asia Pacific | 3334096.95(2433633.41,4377966.85) | 1908353.12(1413354.16,2501904.66) | 7533.15(5486.96,9939.85) | 7408.53(5479.12,9740.88) | -0.07(-0.08,-0.06) |
| High-income North America | 7172887.72(5304693.31,9257067.44) | 8237933.26(6121132.01,10775679.97) | 11552.85(8541.89,14933.98) | 11189.36(8300.11,14664.37) | 0.03(-0.14,0.20) |
| North Africa and Middle East | 14742930.33(10589256.86,19725551.65) | 21667137.65(15792364.94,28555763.81) | 12056.43(8670.69,16110.12) | 12398.69(9039.14,16335.99) | 0.12(0.09,0.14) |
| Oceania | 223309.31(157339.25,304255.74) | 420794.84(296538.09,573205.39) | 9693.16(6840.77,13185.82) | 9674.15(6827.14,13160.86) | -0.01(-0.01,-0.01) |
| South Asia | 40316771.70(29699310.09,52945347.93) | 59338336.40(44447335.60,77409866.19) | 10891.53(8032.76,14282.12) | 10810.62(8086.02,14122.12) | -0.05(-0.08,-0.03) |
| Southeast Asia | 18053485.60(13182962.33,23661518.61) | 19562535.90(14245746.34,25537824.44) | 11187.31(8168.56,14658.97) | 10963.85(7979.38,14331.01) | -0.06(-0.08,-0.05) |
| Southern Latin America | 1114223.48(781201.98,1495040.32) | 1237143.01(869339.87,1682304.29) | 7752.67(5433.23,10407.59) | 7869.07(5521.32,10721.35) | 0.08(0.06,0.09) |
| Southern Sub-Saharan Africa | 1636631.02(1189411.38,2145535.77) | 2021267.13(1468512.60,2653216.62) | 8673.71(6304.78,11367.68) | 8629.34(6267.86,11329.18) | -0.02(-0.02,-0.02) |
| Tropical Latin America | 10027984.51(7787922.17,12632304.42) | 10202391.79(7970646.83,12962775.51) | 19237.38(14946.82,24225.12) | 20190.52(15753.44,25680.25) | 0.37(0.20,0.53) |
| Western Europe | 10715643.88(7751313.60,14088512.19) | 9732297.28(7065359.00,12798842.46) | 13235.39(9551.98,17472.09) | 13210.62(9574.42,17406.03) | 0.02(0.00,0.03) |
| Western Sub-Saharan Africa | 7526371.74(5448414.26,10021203.19) | 19900230.64(14435287.61,26563280.49) | 11088.40(8046.85,14732.16) | 10888.48(7908.52,14512.34) | -0.06(-0.07,-0.05) |

Abbreviations: ASR, age-standardized rate; EAPC, estimated annual percentage change; UI, uncertainty interval; CI, confidence interval
