# Supplementary material for: Rising trends in the burden of migraine among children and adolescents: a comprehensive analysis from 1990 to 2021 with future predictions
Source: Front Public Health. 2025 Oct 23;13:1634098. doi: 10.3389/fpubh.2025.1634098 (PMC12589008; doi:10.3389/fpubh.2025.1634098)
Supplement: Supplementary table S2 — Incidence of migraine in children and adolescents aged 5 to 19 years in 1990 and 2021 at global and regional levels, with EAPCs from 1990 to 2021. [file Table_2.docx]

Table S2. Incidence of migraine in children and adolescents aged 5 to 19 years in 1990 and 2021 at global and regional levels, with EAPCs from 1990 to 2021

| Location | Incidence | | | | |
| --- | --- | --- | --- | --- | --- |
|  | Number of cases(95% UI) | | ASR per 100,000 population (95% UI) | | EAPC(95% CI) |
|  | 1990 | 2021 | 1990 | 2021 | 1990-2021 |
| Global | 30159792.80(20388195.20,41772246.04) | 36794857.75(24894466.96,50875831.49) | 1845.92(1249.97,2553.58) | 1858.23(1257.53,2569.01) | 0.06(0.04,0.08) |
| High SDI | 3817496.33(2526126.75,5379769.80) | 3742835.92(2479098.88,5280870.09) | 2006.60(1332.26,2819.70) | 2075.70(1376.65,2925.56) | 0.15(0.10,0.20) |
| High-middle SDI | 4519369.60(3014993.86,6326292.67) | 3929414.88(2625896.45,5478636.24) | 1628.62(1090.82,2271.87) | 1681.57(1123.67,2344.71) | 0.21(0.17,0.25) |
| Middle SDI | 10300212.51(7046210.81,14177842.48) | 10832364.58(7384331.94,14883378.80) | 1835.90(1259.92,2521.25) | 1890.30(1289.24,2596.33) | 0.15(0.12,0.19) |
| Low-middle SDI | 8382771.31(5700884.44,11554306.41) | 11368438.93(7751647.90,15673846.20) | 2003.52(1359.60,2764.74) | 1985.74(1355.16,2736.35) | -0.03(-0.04,-0.02) |
| Low SDI | 3112432.76(2069445.27,4336656.22) | 6893720.79(4594843.48,9644385.96) | 1649.27(1093.45,2302.44) | 1644.21(1094.19,2302.67) | -0.01(-0.02,-0.00) |
| Andean Latin America | 211393.71(143370.87,294050.46) | 277809.82(182356.27,397481.62) | 1538.22(1041.53,2141.93) | 1594.45(1047.87,2280.62) | 0.13(0.10,0.17) |
| Australasia | 83923.67(53473.03,120500.32) | 101300.99(64868.80,144936.65) | 1758.89(1125.70,2517.45) | 1756.51(1124.18,2514.26) | 0.00(-0.00,0.00) |
| Caribbean | 238311.82(156157.94,342732.36) | 247731.95(162617.04,355718.61) | 2206.24(1452.26,3163.86) | 2197.67(1446.40,3151.22) | -0.01(-0.02,-0.01) |
| Central Asia | 374690.70(235605.01,555118.74) | 416744.06(262779.16,616449.74) | 1704.89(1072.09,2525.44) | 1695.68(1066.16,2512.54) | -0.02(-0.02,-0.02) |
| Central Europe | 511377.15(334445.80,730376.88) | 304519.89(199263.95,434131.30) | 1682.34(1099.47,2403.45) | 1678.71(1097.45,2394.24) | -0.02(-0.02,-0.01) |
| Central Latin America | 1223867.12(849193.88,1683459.18) | 1330912.32(907149.05,1840440.28) | 2049.41(1420.66,2820.79) | 2064.95(1410.80,2851.08) | 0.04(0.03,0.04) |
| Central Sub-Saharan Africa | 324490.71(203194.28,471541.84) | 830080.89(520231.28,1205625.55) | 1582.88(988.76,2302.75) | 1581.78(988.04,2301.39) | -0.00(-0.00,-0.00) |
| East Asia | 4511442.84(3000596.02,6339385.90) | 3659878.22(2467826.57,5109905.83) | 1301.97(872.47,1819.22) | 1385.96(933.21,1936.20) | 0.24(0.19,0.29) |
| Eastern Europe | 810309.72(546614.59,1131976.00) | 586781.46(397601.64,817298.59) | 1620.73(1094.43,2262.07) | 1621.98(1096.94,2262.95) | 0.00(-0.00,0.01) |
| Eastern Sub-Saharan Africa | 792551.99(517964.97,1130293.86) | 1763122.70(1146100.19,2524154.21) | 1065.40(694.12,1523.08) | 1076.29(698.88,1542.01) | 0.06(0.05,0.07) |
| High-income Asia Pacific | 591921.80(388221.18,845118.06) | 351939.11(233575.89,501063.30) | 1448.88(955.32,2062.47) | 1427.69(949.45,2030.34) | -0.07(-0.09,-0.06) |
| High-income North America | 1353727.55(912027.44,1877667.19) | 1553397.57(1045927.66,2161312.73) | 2245.64(1515.04,3111.38) | 2209.30(1488.32,3075.57) | 0.06(-0.08,0.19) |
| North Africa and Middle East | 2629575.93(1751674.84,3703510.47) | 3742743.64(2515435.03,5248779.39) | 2083.36(1385.00,2937.67) | 2129.58(1429.76,2988.51) | 0.09(0.07,0.10) |
| Oceania | 42752.19(27155.96,62352.16) | 80423.66(51056.73,117341.57) | 1812.99(1149.53,2647.97) | 1811.01(1148.47,2644.86) | -0.00(-0.01,-0.00) |
| South Asia | 7609055.05(5175184.61,10531999.13) | 10311022.72(6982859.01,14226752.50) | 1975.31(1341.04,2735.90) | 1962.81(1331.75,2704.90) | -0.05(-0.07,-0.03) |
| Southeast Asia | 3392685.01(2245713.41,4769026.07) | 3591372.55(2369368.21,5055851.30) | 2096.05(1386.35,2948.18) | 2067.35(1365.45,2907.76) | -0.04(-0.05,-0.03) |
| Southern Latin America | 212833.75(134262.73,313556.62) | 232556.85(146600.05,341582.08) | 1491.62(941.17,2197.30) | 1518.10(958.84,2226.18) | 0.09(0.07,0.10) |
| Southern Sub-Saharan Africa | 305442.25(201836.34,429470.37) | 373454.39(246507.80,526119.82) | 1610.07(1063.66,2264.14) | 1602.59(1057.32,2258.68) | -0.02(-0.02,-0.02) |
| Tropical Latin America | 1642606.12(1196813.65,2162944.18) | 1577341.95(1159828.69,2081444.50) | 3128.34(2275.09,4125.25) | 3262.20(2404.36,4298.24) | 0.32(0.18,0.46) |
| Western Europe | 1866990.94(1205670.85,2654398.15) | 1749526.19(1134547.73,2485994.23) | 2468.17(1603.47,3495.54) | 2467.81(1603.09,3502.35) | 0.02(0.01,0.03) |
| Western Sub-Saharan Africa | 1429842.76(953187.11,1990130.26) | 3712196.83(2479495.94,5197325.11) | 1994.02(1327.86,2779.05) | 1966.16(1310.64,2756.12) | -0.05(-0.06,-0.04) |

Abbreviations: ASR, age-standardized rate; EAPC, estimated annual percentage change; UI, uncertainty interval; CI, confidence interval
