# Supplementary material for: Rising trends in the burden of migraine among children and adolescents: a comprehensive analysis from 1990 to 2021 with future predictions
Source: Front Public Health. 2025 Oct 23;13:1634098. doi: 10.3389/fpubh.2025.1634098 (PMC12589008; doi:10.3389/fpubh.2025.1634098)
Supplement: Supplementary table S3 — DALYs of migraine in children and adolescents aged 5 to 19 years in 1990 and 2021 at global and regional levels, with EAPCs from 1990 to 2021. [file Table_3.docx]

Table S3. DALYs of migraine in children and adolescents aged 5 to 19 years in 1990 and 2021 at global and regional levels, with EAPCs from 1990 to 2021

| Location | DALYs | | | | |
| --- | --- | --- | --- | --- | --- |
|  | Number of cases(95% UI) | | ASR per 100,000 population (95% UI) | | EAPC(95% CI) |
|  | 1990 | 2021 | 1990 | 2021 | 1990-2021 |
| Global | 6042775.97(395006.19,15051635.05) | 7515775.31(484365.23,18769692.03) | 366.11(23.73,912.43) | 374.50(24.02,935.71) | 0.11(0.09,0.12) |
| High SDI | 757485.09(52659.84,1855355.30) | 732009.88(49039.73,1788214.39) | 380.16(25.75,934.35) | 390.82(25.73,957.63) | 0.14(0.08,0.20) |
| High-middle SDI | 940958.87(78279.76,2276674.59) | 792547.94(61452.38,1946891.06) | 322.21(26.07,782.56) | 337.41(26.12,829.05) | 0.23(0.18,0.28) |
| Middle SDI | 2126579.39(132719.00,5330829.83) | 2260066.96(137304.20,5687767.10) | 367.26(22.47,921.62) | 387.25(23.36,975.00) | 0.21(0.18,0.24) |
| Low-middle SDI | 1632620.90(89542.63,4099643.00) | 2367329.51(136646.20,5901590.28) | 405.27(22.65,1015.99) | 403.37(23.05,1006.33) | -0.01(-0.03,0.01) |
| Low SDI | 579429.77(41130.04,1431541.74) | 1357954.95(99197.12,3358735.73) | 327.33(23.94,805.01) | 329.07(24.27,813.13) | 0.02(0.02,0.03) |
| Andean Latin America | 44269.23(3103.23,109593.89) | 60143.28(4116.76,150704.97) | 325.43(23.00,805.12) | 338.59(22.94,849.22) | 0.16(0.12,0.20) |
| Australasia | 16292.47(1223.66,40604.86) | 18904.47(1345.66,47610.80) | 322.73(23.58,807.63) | 323.23(22.93,814.71) | 0.00(-0.00,0.01) |
| Caribbean | 53211.85(2603.54,132440.57) | 54876.40(2671.47,136633.24) | 471.57(22.48,1175.44) | 468.88(22.38,1168.91) | -0.02(-0.02,-0.02) |
| Central Asia | 71781.69(5394.84,176667.72) | 78336.71(5766.65,194452.93) | 331.85(25.07,816.00) | 330.78(24.81,819.81) | -0.01(-0.01,-0.00) |
| Central Europe | 103944.16(9254.41,259014.97) | 61991.20(5698.20,153233.90) | 331.66(29.28,827.68) | 330.72(30.14,818.85) | -0.01(-0.01,-0.00) |
| Central Latin America | 263480.72(14491.65,658632.40) | 301444.99(16921.67,750530.12) | 440.82(24.31,1101.91) | 445.77(24.45,1111.27) | 0.05(0.04,0.06) |
| Central Sub-Saharan Africa | 60150.17(4512.29,148138.62) | 158339.97(12837.34,389765.17) | 308.72(23.68,757.72) | 312.40(25.80,767.81) | 0.04(0.04,0.05) |
| East Asia | 938816.24(78386.13,2296032.86) | 691375.69(51089.58,1679292.14) | 249.84(20.02,614.66) | 266.97(19.90,647.51) | 0.26(0.22,0.31) |
| Eastern Europe | 166269.11(19859.72,406956.45) | 118079.97(13993.15,285872.74) | 329.16(39.06,806.70) | 329.84(39.46,797.34) | 0.02(0.01,0.02) |
| Eastern Sub-Saharan Africa | 147491.88(16930.85,363381.97) | 343637.67(39044.69,861114.59) | 208.97(24.58,512.36) | 211.80(24.19,530.31) | 0.08(0.07,0.10) |
| High-income Asia Pacific | 118873.45(11292.75,305973.93) | 67997.01(6255.87,173514.42) | 267.51(24.49,694.21) | 263.38(23.76,674.48) | -0.06(-0.07,-0.05) |
| High-income North America | 258638.42(15970.92,633530.03) | 294494.55(19116.62,723724.60) | 416.17(25.27,1021.91) | 399.48(25.40,986.86) | 0.01(-0.16,0.18) |
| North Africa and Middle East | 564205.90(40703.60,1402175.38) | 829916.65(59587.86,2053535.06) | 461.96(33.92,1147.29) | 475.02(34.27,1175.15) | 0.12(0.10,0.14) |
| Oceania | 8155.69(454.38,20455.50) | 15432.11(852.87,38098.34) | 354.29(19.99,887.90) | 355.02(19.79,875.97) | 0.01(0.01,0.02) |
| South Asia | 1433305.02(74291.04,3569497.66) | 2128743.46(116421.23,5268538.11) | 387.83(20.47,962.92) | 387.17(20.77,960.58) | -0.03(-0.05,-0.01) |
| Southeast Asia | 670216.27(33197.44,1675055.99) | 729450.94(37098.94,1825627.46) | 415.36(20.64,1038.05) | 408.48(20.53,1022.84) | -0.05(-0.06,-0.04) |
| Southern Latin America | 40081.92(3463.98,100469.12) | 44400.81(3762.27,112084.22) | 278.79(24.03,699.15) | 282.08(23.58,713.42) | 0.07(0.06,0.09) |
| Southern Sub-Saharan Africa | 59345.34(5004.96,145076.69) | 73377.26(6391.48,180781.05) | 314.60(26.59,768.73) | 313.21(27.27,772.01) | -0.01(-0.01,-0.01) |
| Tropical Latin America | 368264.18(11837.33,925550.70) | 374580.22(11808.81,936210.60) | 706.48(22.96,1775.36) | 740.98(22.78,1853.05) | 0.39(0.22,0.56) |
| Western Europe | 386004.15(21263.73,963122.44) | 349458.82(19071.42,867338.54) | 475.91(25.24,1191.62) | 473.87(25.44,1178.13) | 0.02(0.00,0.03) |
| Western Sub-Saharan Africa | 269978.10(17489.41,676101.19) | 720793.13(46089.79,1821893.96) | 398.66(26.55,995.05) | 394.94(25.70,996.52) | -0.03(-0.04,-0.02) |

Abbreviations: ASR, age-standardized rate; DALYs, disability-adjusted life years; EAPC, estimated annual percentage change; UI, uncertainty interval; CI, confidence interval
