# Supplementary material for: Rising trends in the burden of migraine among children and adolescents: a comprehensive analysis from 1990 to 2021 with future predictions
Source: Front Public Health. 2025 Oct 23;13:1634098. doi: 10.3389/fpubh.2025.1634098 (PMC12589008; doi:10.3389/fpubh.2025.1634098)
Supplement: Supplementary table S4 — Prevalence of migraine in children and adolescents aged 5 to 19 years in 1990 and 2021 across 204 countries and territories, with EAPCs from 1990 to 2021. [file Table_4.docx]

Table S4. Prevalence of migraine in children and adolescents aged 5 to 19 years in 1990 and 2021 across 204 countries and territories, with EAPCs from 1990 to 2021

| Location | Prevalence | | | | |
| --- | --- | --- | --- | --- | --- |
|  | Number of cases(95% UI) | | ASR per 100,000 population (95% UI) | | EAPC(95% CI) |
|  | 1990 | 2021 | 1990 | 2021 | 1990-2021 |
| Afghanistan | 480125.28(341157.10,652618.59) | 1439697.70(1020581.30,1964095.67) | 12122.18(8599.53,16504.89) | 12059.14(8561.44,16429.02) | -0.01(-0.01,-0.00) |
| Albania | 95670.77(67251.87,128027.62) | 46129.83(32512.28,61495.06) | 9135.98(6420.19,12236.50) | 9053.55(6360.47,12133.60) | -0.04(-0.05,-0.03) |
| Algeria | 1144245.33(810547.66,1561562.45) | 1347068.17(952834.21,1841515.86) | 12081.42(8574.78,16456.08) | 12075.60(8571.57,16449.03) | -0.00(-0.00,0.00) |
| American Samoa | 1551.99(1094.41,2112.28) | 1557.27(1100.66,2116.94) | 9682.76(6833.29,13171.34) | 9698.51(6844.25,13194.82) | 0.01(0.01,0.02) |
| Andorra | 1519.17(1071.45,2035.37) | 1678.31(1184.04,2252.72) | 12926.92(9092.10,17413.52) | 12945.80(9105.38,17443.03) | -0.01(-0.02,0.00) |
| Angola | 312664.98(216725.16,425592.04) | 1057242.09(732538.57,1440949.60) | 8624.72(5997.18,11701.90) | 8663.18(6029.83,11752.62) | 0.01(0.00,0.02) |
| Antigua and Barbuda | 2340.97(1655.97,3162.56) | 2487.99(1765.42,3350.30) | 12942.21(9145.80,17501.15) | 12882.16(9104.15,17412.72) | -0.04(-0.05,-0.02) |
| Argentina | 739896.02(516041.31,995367.84) | 865672.88(608216.73,1185852.80) | 7721.06(5387.54,10378.28) | 7833.54(5495.74,10752.18) | 0.07(0.06,0.09) |
| Armenia | 85087.48(59626.73,115315.69) | 52034.24(36421.27,70581.61) | 9170.85(6431.26,12422.90) | 9085.04(6364.11,12306.65) | -0.05(-0.06,-0.03) |
| Australia | 376950.54(261861.78,509119.09) | 437609.14(303221.37,593065.37) | 9084.62(6292.09,12319.12) | 9074.06(6284.21,12302.90) | -0.00(-0.00,-0.00) |
| Austria | 184525.41(131567.78,248077.37) | 168006.97(119546.14,226553.36) | 12149.09(8626.96,16432.56) | 12104.27(8589.49,16383.52) | 0.03(0.01,0.06) |
| Azerbaijan | 203729.82(142893.63,275720.97) | 209164.03(146378.33,283740.05) | 9156.71(6419.66,12405.44) | 9076.11(6357.31,12294.40) | -0.04(-0.06,-0.03) |
| Bahamas | 10874.10(7698.17,14679.84) | 13382.26(9497.31,18028.03) | 12934.39(9140.55,17490.01) | 12974.58(9168.70,17548.66) | 0.01(0.01,0.01) |
| Bahrain | 15767.04(11144.96,21549.55) | 38443.14(27421.57,52271.32) | 12108.24(8590.87,16487.41) | 11950.35(8497.77,16299.52) | -0.04(-0.06,-0.03) |
| Bangladesh | 4364771.33(3048488.36,5939648.90) | 5352363.83(3750688.39,7254968.22) | 11106.19(7773.34,15074.44) | 11119.84(7782.85,15093.68) | 0.02(0.01,0.03) |
| Barbados | 8846.91(6271.36,11925.78) | 7350.09(5218.71,9890.79) | 12902.26(9117.87,17442.85) | 12863.57(9091.11,17385.63) | -0.01(-0.01,-0.00) |
| Belarus | 197898.55(139672.95,267399.38) | 127607.61(89847.19,172971.86) | 8439.89(5954.20,11413.84) | 8396.16(5920.38,11354.53) | -0.02(-0.02,-0.01) |
| Belgium | 299210.92(211219.92,401033.76) | 315108.54(227759.54,428281.19) | 15048.14(10588.56,20237.94) | 15625.78(11275.52,21269.96) | 0.13(0.09,0.17) |
| Belize | 9168.25(6465.10,12422.59) | 17923.93(12711.01,24160.30) | 12939.75(9144.19,17497.94) | 12953.63(9153.93,17518.19) | 0.00(0.00,0.01) |
| Benin | 181582.04(126798.20,248332.89) | 536464.97(376032.70,730425.35) | 10846.44(7621.03,14725.55) | 10873.41(7640.32,14760.47) | 0.01(0.01,0.01) |
| Bermuda | 1545.68(1095.53,2083.88) | 1220.83(865.20,1646.11) | 12908.86(9122.47,17451.97) | 12903.72(9119.02,17444.85) | -0.00(-0.01,0.01) |
| Bhutan | 27178.29(19020.29,36865.26) | 22733.06(15943.09,30748.41) | 10929.82(7644.24,14841.56) | 11083.93(7755.66,15042.51) | 0.04(0.04,0.05) |
| Bolivia (Plurinational State of) | 220724.12(155687.23,298034.82) | 327275.09(231384.47,440779.05) | 9701.22(6854.51,13073.38) | 9667.04(6830.63,13028.47) | -0.01(-0.02,-0.01) |
| Bosnia and Herzegovina | 107846.36(75933.34,144060.44) | 49194.02(34638.32,65731.88) | 9116.28(6405.76,12215.55) | 9114.89(6404.82,12213.41) | -0.02(-0.03,-0.01) |
| Botswana | 44592.52(30985.07,60638.82) | 60011.36(41779.57,81328.33) | 8686.16(6049.54,11782.93) | 8617.50(5992.19,11692.92) | -0.03(-0.03,-0.03) |
| Brazil | 9754031.98(7577484.33,12285489.68) | 9798609.69(7663261.01,12439930.67) | 19227.60(14942.65,24210.09) | 20220.58(15794.05,25697.68) | 0.38(0.21,0.55) |
| Brunei Darussalam | 5716.95(3999.81,7831.32) | 7430.25(5211.46,10136.51) | 7214.82(5050.78,9881.54) | 7151.90(5006.40,9790.61) | -0.04(-0.05,-0.02) |
| Bulgaria | 177698.50(125181.63,237418.42) | 93515.95(65795.17,125203.62) | 9124.62(6411.67,12226.06) | 9106.87(6399.16,12203.19) | -0.01(-0.01,-0.00) |
| Burkina Faso | 376240.79(263102.23,514062.81) | 907503.76(636233.13,1235287.78) | 10859.59(7630.41,14742.63) | 10901.22(7659.88,14797.83) | 0.01(0.01,0.01) |
| Burundi | 113578.00(78821.22,153928.82) | 293132.57(203891.03,396541.74) | 5959.47(4155.75,8036.22) | 5968.86(4162.58,8049.43) | 0.01(0.00,0.02) |
| Cabo Verde | 13895.29(9737.12,18926.70) | 16816.14(11839.86,22785.39) | 10858.26(7629.62,14743.09) | 10841.38(7617.87,14722.13) | -0.01(-0.01,-0.00) |
| Cambodia | 399672.93(277972.33,543822.73) | 537388.57(375026.66,728225.56) | 11024.42(7692.97,14940.50) | 10921.12(7619.75,14802.38) | -0.03(-0.03,-0.03) |
| Cameroon | 400254.01(280309.44,545494.33) | 1267308.33(889042.87,1724817.58) | 10861.32(7631.65,14744.90) | 10841.55(7617.93,14721.06) | -0.01(-0.01,-0.01) |
| Canada | 663775.99(473635.97,889779.59) | 719168.88(511096.18,968932.98) | 11149.42(7942.14,14985.82) | 10968.56(7783.37,14796.61) | -0.05(-0.05,-0.04) |
| Central African Republic | 80974.75(56161.61,110204.91) | 175149.48(121733.83,237907.74) | 8632.03(6005.63,11712.17) | 8638.27(6010.10,11720.46) | 0.01(0.00,0.01) |
| Chad | 230929.93(161645.74,314902.12) | 742205.46(519660.05,1011948.65) | 10897.87(7657.51,14793.24) | 10870.59(7638.43,14756.71) | -0.01(-0.01,-0.01) |
| Chile | 309153.75(215435.96,418765.73) | 313175.74(220039.96,422739.96) | 7802.32(5424.42,10604.80) | 7970.96(5593.19,10778.10) | 0.11(0.08,0.14) |
| China | 24476342.35(18154656.79,32384237.92) | 18087810.40(13275385.79,23863271.88) | 6743.73(4991.26,8953.32) | 7222.70(5302.57,9522.30) | 0.27(0.22,0.32) |
| Colombia | 1374297.70(994223.93,1846958.29) | 1485866.96(1070283.80,2017697.42) | 12590.08(9104.09,16924.55) | 12670.16(9078.99,17256.39) | 0.04(0.02,0.05) |
| Comoros | 10440.57(7263.96,14110.99) | 14024.60(9785.14,18892.41) | 5935.79(4138.36,8002.99) | 5929.09(4133.43,7993.60) | -0.00(-0.01,-0.00) |
| Congo | 79159.17(55005.34,107544.58) | 160111.23(111321.87,217419.62) | 8651.93(6020.15,11737.62) | 8641.34(6012.26,11724.32) | -0.01(-0.01,-0.00) |
| Cook Islands | 627.68(443.35,853.31) | 424.30(300.49,575.52) | 9700.22(6845.73,13196.87) | 9796.13(6914.40,13325.55) | 0.03(0.01,0.04) |
| Costa Rica | 122486.64(87131.21,168671.16) | 135742.40(97010.05,186365.28) | 12356.30(8807.61,16993.08) | 12372.69(8819.14,17015.99) | 0.01(0.01,0.02) |
| Croatia | 97277.00(68465.17,130082.17) | 59276.40(42533.90,80472.01) | 9123.56(6410.99,12224.73) | 9029.91(6459.91,12284.95) | -0.14(-0.22,-0.07) |
| Cuba | 389961.04(277962.72,522790.91) | 246311.01(174501.96,332067.57) | 12854.25(9084.47,17372.09) | 12831.32(9068.73,17337.69) | -0.01(-0.01,-0.01) |
| Cyprus | 25462.95(17915.63,34276.37) | 27563.74(19397.89,37093.92) | 12933.21(9097.15,17418.97) | 12935.46(9098.90,17421.30) | 0.02(0.01,0.02) |
| Czechia | 237646.29(167521.33,317389.77) | 153625.83(107989.79,206015.59) | 9126.57(6412.98,12228.69) | 9118.75(6407.54,12218.66) | -0.00(-0.00,-0.00) |
| Cote d'Ivoire | 463184.87(324201.96,631552.66) | 1037705.83(727568.77,1413715.06) | 10850.57(7624.16,14731.32) | 10779.77(7574.43,14645.89) | -0.02(-0.03,-0.02) |
| Democratic People's Republic of Korea | 449153.31(311762.47,611902.40) | 427313.27(296904.50,581223.45) | 7984.61(5528.84,10900.41) | 7817.09(5408.19,10669.70) | -0.05(-0.07,-0.04) |
| Democratic Republic of the Congo | 1169303.90(810417.07,1591674.34) | 2881530.31(1999493.47,3917783.66) | 8621.52(5994.73,11697.83) | 8608.42(5984.46,11681.03) | -0.00(-0.01,-0.00) |
| Denmark | 120115.57(84729.91,162062.36) | 119109.64(85529.92,160693.20) | 11231.83(7885.00,15257.43) | 11373.13(8146.81,15385.56) | 0.04(0.03,0.06) |
| Djibouti | 9487.53(6614.83,12786.30) | 22075.59(15363.37,29808.28) | 5874.35(4093.37,7921.27) | 5811.71(4046.79,7841.87) | -0.06(-0.07,-0.05) |
| Dominica | 3176.08(2249.08,4283.09) | 2208.99(1567.80,2973.55) | 12802.66(9048.78,17295.61) | 12862.96(9090.70,17384.27) | 0.00(-0.01,0.02) |
| Dominican Republic | 332589.61(235301.60,449565.76) | 377869.23(267618.23,509838.55) | 13044.06(9217.45,17651.27) | 12907.47(9121.79,17450.42) | -0.03(-0.04,-0.03) |
| Ecuador | 395655.17(289790.16,525727.68) | 559794.65(396979.98,764268.97) | 10972.77(8039.96,14575.41) | 11148.91(7903.37,15225.12) | 0.08(0.07,0.09) |
| Egypt | 2342287.55(1661631.80,3160805.91) | 4554936.57(3461385.26,5816109.88) | 12242.16(8690.39,16499.89) | 13949.37(10614.18,17793.70) | 0.59(0.47,0.71) |
| El Salvador | 247123.92(176033.42,339985.16) | 221114.66(157765.44,303900.43) | 12410.35(8847.95,17064.83) | 12327.28(8786.70,16953.40) | -0.02(-0.02,-0.02) |
| Equatorial Guinea | 12864.36(8925.74,17515.54) | 49749.32(34453.72,67458.30) | 8655.96(6026.73,11744.85) | 8358.72(5783.80,11343.94) | -0.14(-0.14,-0.13) |
| Eritrea | 75825.39(52715.18,102520.20) | 133936.21(93288.82,180688.09) | 5895.82(4109.25,7948.63) | 5899.47(4111.50,7954.22) | 0.00(0.00,0.01) |
| Estonia | 29091.84(20538.02,39274.64) | 18090.01(12761.73,24490.60) | 8407.39(5928.14,11371.62) | 8410.56(5931.53,11373.90) | -0.01(-0.01,-0.00) |
| Eswatini | 27475.42(19070.41,37395.72) | 34138.45(23731.44,46323.36) | 8675.20(6040.85,11768.57) | 8583.68(5965.69,11649.30) | -0.03(-0.04,-0.02) |
| Ethiopia | 939355.57(691598.60,1230478.68) | 2144244.51(1577951.48,2805369.87) | 5118.91(3773.88,6687.24) | 5158.23(3795.35,6750.38) | 0.03(0.02,0.04) |
| Fiji | 25245.80(17791.97,34396.05) | 25157.69(17745.33,34250.38) | 9730.99(6867.63,13238.12) | 9733.31(6869.25,13241.78) | 0.00(-0.00,0.00) |
| Finland | 126787.73(89271.79,170630.74) | 123240.98(86847.60,165642.98) | 12993.39(9142.12,17501.28) | 12990.21(9139.73,17497.02) | -0.00(-0.00,0.00) |
| France | 1680108.69(1186786.79,2262697.24) | 1632082.45(1172387.33,2178035.46) | 12923.68(9096.08,17489.96) | 12697.69(9097.14,16990.99) | -0.09(-0.12,-0.07) |
| Gabon | 29511.15(20504.26,40120.18) | 53587.92(37341.70,72698.76) | 8675.01(6040.21,11768.44) | 8719.69(6077.05,11826.95) | 0.03(0.02,0.03) |
| Gambia | 38353.03(26858.64,52274.92) | 98212.81(68980.71,133424.81) | 10882.66(7646.78,14772.95) | 10881.62(7646.20,14771.11) | 0.00(0.00,0.00) |
| Georgia | 124364.63(87320.80,168227.84) | 60182.16(42063.37,81760.87) | 9179.16(6437.52,12434.66) | 9091.83(6368.34,12318.55) | -0.05(-0.06,-0.04) |
| Germany | 1853367.52(1316277.52,2499106.36) | 1718470.52(1213891.87,2294868.13) | 13868.46(9827.31,18747.98) | 13983.45(9863.26,18713.21) | 0.08(0.04,0.11) |
| Ghana | 577330.52(404542.50,786610.01) | 1238163.48(869066.25,1683662.83) | 10829.28(7609.24,14706.28) | 10831.64(7610.94,14709.19) | 0.01(0.00,0.01) |
| Greece | 329042.17(236250.26,442737.03) | 214390.76(152839.82,290232.57) | 13703.53(9808.78,18496.72) | 13494.50(9588.20,18324.06) | -0.06(-0.07,-0.05) |
| Greenland | 1315.14(925.78,1787.34) | 1284.87(907.00,1741.43) | 10991.94(7748.21,14922.39) | 11122.85(7845.05,15090.22) | 0.04(0.03,0.05) |
| Grenada | 3807.06(2686.36,5153.99) | 3154.52(2238.50,4244.78) | 12897.56(9114.90,17435.56) | 12783.57(9035.27,17266.96) | -0.05(-0.07,-0.03) |
| Guam | 3734.99(2639.96,5072.40) | 3628.51(2567.01,4926.16) | 9686.78(6835.76,13181.72) | 9718.68(6858.85,13222.36) | -0.01(-0.01,-0.00) |
| Guatemala | 395487.76(280015.22,546237.41) | 640893.09(458142.71,879796.03) | 12486.83(8902.85,17169.75) | 12395.10(8835.42,17046.54) | -0.02(-0.03,-0.02) |
| Guinea | 210688.57(147329.90,287679.42) | 538785.60(377547.10,734104.32) | 10858.10(7629.33,14740.75) | 10875.29(7641.64,14763.01) | 0.01(-0.00,0.02) |
| Guinea-Bissau | 40732.91(28520.38,55541.79) | 82097.46(57568.05,111777.81) | 10885.32(7648.60,14776.60) | 10854.29(7626.92,14736.85) | -0.01(-0.01,-0.01) |
| Guyana | 36015.30(25489.15,48649.02) | 27099.31(19181.57,36584.12) | 12997.68(9184.62,17582.39) | 12913.67(9126.03,17459.52) | -0.02(-0.03,-0.02) |
| Haiti | 287430.48(202543.95,389955.84) | 523431.39(369996.48,707641.15) | 13058.77(9227.26,17672.28) | 12941.76(9145.66,17500.68) | -0.04(-0.05,-0.04) |
| Honduras | 223627.17(158624.29,308504.86) | 413660.80(295587.96,567984.55) | 12387.40(8830.82,17034.45) | 12365.65(8814.29,17006.03) | -0.00(-0.00,0.00) |
| Hungary | 225096.64(158611.57,301054.30) | 136314.04(95994.50,182091.30) | 9120.06(6408.43,12220.35) | 9113.34(6403.79,12211.58) | -0.01(-0.01,-0.00) |
| Iceland | 8490.72(5979.35,11407.82) | 9042.79(6370.88,12166.64) | 12992.69(9141.74,17500.97) | 13002.29(9148.35,17510.08) | 0.00(0.00,0.01) |
| India | 30811589.30(22717859.89,40397910.04) | 44452953.56(33453805.57,58055510.87) | 10876.95(8028.00,14241.88) | 10789.03(8103.10,14110.74) | -0.06(-0.09,-0.03) |
| Indonesia | 7147695.78(5239399.46,9322716.82) | 7790082.25(5712785.39,10147690.52) | 10986.40(8052.67,14325.52) | 10935.13(8013.33,14266.25) | -0.02(-0.02,-0.02) |
| Iran (Islamic Republic of) | 2612191.80(1965931.53,3362427.65) | 2369192.62(1786632.20,3041484.25) | 12301.53(9279.37,15798.28) | 12331.93(9310.89,15804.82) | -0.12(-0.25,0.01) |
| Iraq | 835277.98(592044.88,1139881.49) | 1628660.16(1156802.87,2217991.56) | 12054.66(8559.30,16424.05) | 12062.40(8563.69,16433.23) | 0.00(0.00,0.01) |
| Ireland | 140895.13(99269.02,189332.61) | 136878.97(96417.03,184265.43) | 12983.22(9134.56,17486.35) | 12998.42(9145.89,17507.62) | 0.00(0.00,0.01) |
| Israel | 196286.81(138162.34,264135.08) | 316604.41(222701.21,426590.34) | 12970.89(9125.36,17471.01) | 12984.64(9135.60,17488.91) | 0.00(0.00,0.01) |
| Italy | 1740541.27(1302284.66,2237983.77) | 1251191.61(934918.50,1614040.23) | 14068.03(10486.56,18194.48) | 14111.77(10524.13,18243.35) | 0.08(0.00,0.17) |
| Jamaica | 106767.68(75504.05,144299.35) | 88932.04(63112.36,119744.67) | 12960.75(9158.85,17528.78) | 12887.99(9108.07,17421.57) | -0.03(-0.03,-0.02) |
| Japan | 2099186.94(1571962.23,2715047.86) | 1256815.45(930952.72,1648276.48) | 7027.07(5249.33,9131.13) | 7083.30(5242.06,9312.81) | 0.04(0.02,0.06) |
| Jordan | 179319.43(127378.48,244305.35) | 484179.98(344665.93,658216.10) | 12034.45(8547.54,16399.93) | 12049.33(8556.17,16417.68) | -0.01(-0.01,-0.00) |
| Kazakhstan | 436638.21(306266.31,591676.32) | 420254.44(294006.89,571357.95) | 9206.09(6459.10,12469.83) | 9200.35(6455.63,12459.38) | -0.00(-0.01,-0.00) |
| Kenya | 541079.61(399300.52,710682.79) | 1138068.78(842043.34,1490805.80) | 5976.27(4416.75,7833.15) | 5951.40(4399.76,7800.98) | -0.01(-0.01,-0.01) |
| Kiribati | 2306.03(1623.58,3142.60) | 3763.65(2652.73,5127.71) | 9733.48(6869.34,13241.00) | 9760.71(6888.98,13277.18) | 0.01(0.00,0.01) |
| Kuwait | 56685.87(39739.24,76184.39) | 101500.35(71513.07,138633.07) | 12306.30(8645.90,16494.82) | 12193.54(8593.36,16647.84) | -0.03(-0.04,-0.02) |
| Kyrgyzstan | 132286.98(92733.93,179369.49) | 176982.17(123780.83,240634.64) | 9223.06(6472.90,12491.35) | 9202.28(6456.98,12462.36) | -0.01(-0.01,-0.01) |
| Lao People's Democratic Republic | 162984.94(113432.42,221611.11) | 240289.51(167795.02,325250.73) | 11013.51(7686.07,14924.45) | 10951.46(7640.03,14843.16) | -0.01(-0.01,-0.01) |
| Latvia | 47236.00(33360.90,63693.85) | 24793.50(17486.59,33538.90) | 8414.97(5934.05,11382.07) | 8404.80(5927.22,11366.18) | -0.01(-0.01,-0.00) |
| Lebanon | 114228.09(81108.04,155607.74) | 152580.46(108377.29,207954.33) | 12050.70(8555.96,16418.53) | 12022.18(8538.77,16384.13) | -0.01(-0.02,-0.01) |
| Lesotho | 48587.02(33701.03,66239.49) | 56550.64(39396.06,76586.38) | 8700.21(6063.38,11802.65) | 8621.15(5994.88,11697.48) | -0.02(-0.03,-0.02) |
| Liberia | 90322.13(63201.56,123169.22) | 218286.59(153312.85,296747.94) | 10875.04(7641.11,14763.89) | 10839.94(7616.80,14719.33) | -0.02(-0.03,-0.02) |
| Libya | 201934.16(143165.23,275239.57) | 215869.16(153835.78,292900.83) | 12117.10(8597.36,16499.25) | 12078.28(8573.20,16452.28) | -0.02(-0.03,-0.01) |
| Lithuania | 67308.54(47446.16,90749.96) | 32442.64(22829.05,43828.43) | 7948.17(5588.30,10745.66) | 7930.79(5576.23,10722.54) | -0.08(-0.10,-0.05) |
| Luxembourg | 8432.07(5946.38,11285.81) | 13105.32(9241.67,17552.51) | 12451.37(8770.04,16696.02) | 12418.99(8746.53,16657.01) | -0.02(-0.04,-0.00) |
| Madagascar | 259378.74(180373.24,350766.00) | 636763.05(443570.34,859705.46) | 5945.44(4145.52,8016.56) | 5954.80(4152.31,8029.69) | 0.01(0.01,0.01) |
| Malawi | 208569.53(145054.18,282029.30) | 477275.31(332989.98,643407.63) | 5954.95(4152.42,8029.86) | 5958.08(4154.51,8034.19) | -0.00(-0.01,0.00) |
| Malaysia | 559322.10(391630.67,754598.47) | 805598.62(570948.09,1089370.97) | 9552.21(6692.00,12876.87) | 9878.83(6981.93,13394.05) | 0.16(0.12,0.19) |
| Maldives | 8866.85(6166.81,12061.80) | 10737.12(7492.59,14559.14) | 10963.29(7649.32,14857.90) | 10859.90(7578.89,14722.40) | -0.05(-0.06,-0.04) |
| Mali | 320256.25(224003.54,437225.81) | 994553.38(696979.53,1354730.01) | 10853.32(7625.99,14734.75) | 10847.79(7622.31,14728.93) | -0.00(-0.01,0.00) |
| Malta | 11562.89(8141.42,15560.41) | 8184.32(5761.02,11005.29) | 12951.92(9110.83,17444.11) | 12960.52(9117.77,17452.71) | 0.01(0.01,0.01) |
| Marshall Islands | 1734.04(1216.76,2372.67) | 1745.06(1233.41,2371.14) | 9726.95(6864.65,13232.89) | 9746.24(6878.48,13259.03) | 0.01(0.00,0.01) |
| Mauritania | 78497.22(55006.09,106902.81) | 177259.48(124363.39,241170.58) | 10864.36(7634.15,14748.18) | 10879.27(7644.56,14767.94) | 0.01(0.01,0.01) |
| Mauritius | 36630.94(25564.34,49651.31) | 28277.36(19828.88,38078.29) | 10953.89(7641.57,14847.23) | 10959.43(7645.38,14854.43) | 0.00(0.00,0.00) |
| Mexico | 3735297.54(2785846.32,4782942.12) | 4059124.52(3034354.76,5196420.91) | 11655.00(8689.39,14929.97) | 11751.07(8771.15,15062.07) | 0.04(0.03,0.05) |
| Micronesia (Federated States of) | 3921.73(2759.93,5351.00) | 3272.61(2316.31,4440.63) | 9722.53(6861.78,13224.98) | 9716.32(6857.12,13218.52) | 0.00(-0.00,0.00) |
| Monaco | 505.13(355.93,676.57) | 702.56(494.95,942.67) | 12937.57(9100.62,17418.23) | 12998.54(9144.97,17511.66) | 0.03(0.02,0.04) |
| Mongolia | 72201.00(50619.10,97911.88) | 76713.01(53510.39,104596.08) | 9219.59(6470.12,12486.82) | 9200.45(6455.48,12460.10) | -0.01(-0.01,-0.01) |
| Montenegro | 15203.16(10695.53,20336.60) | 10883.54(7662.67,14541.24) | 9118.18(6406.89,12217.74) | 9077.66(6377.86,12165.00) | -0.02(-0.02,-0.02) |
| Morocco | 1074844.52(762223.59,1464252.12) | 1193750.29(848413.36,1624000.03) | 12118.66(8597.87,16500.78) | 12078.20(8573.06,16452.16) | -0.01(-0.02,-0.01) |
| Mozambique | 289911.99(201481.78,392769.04) | 717172.70(498889.39,970141.38) | 5986.74(4176.90,8075.28) | 5974.50(4167.35,8057.71) | 0.00(-0.00,0.01) |
| Myanmar | 1554725.81(1084882.25,2107062.82) | 1765863.46(1234154.16,2388173.28) | 10993.76(7670.81,14899.50) | 10968.44(7652.45,14865.66) | -0.01(-0.01,-0.01) |
| Namibia | 45840.03(31877.72,62232.21) | 69286.62(48215.89,93991.24) | 8653.16(6021.55,11739.43) | 8645.41(6014.97,11729.32) | -0.00(-0.00,-0.00) |
| Nauru | 328.81(231.30,448.55) | 362.95(256.17,493.72) | 9750.26(6881.35,13264.06) | 9706.94(6850.60,13204.92) | -0.01(-0.01,-0.01) |
| Nepal | 800042.59(558733.67,1098985.62) | 1157280.95(810571.21,1568554.81) | 11888.26(8329.18,16282.17) | 11811.72(8255.22,16057.41) | -0.02(-0.03,-0.02) |
| Netherlands | 382455.60(274923.60,513338.54) | 382900.09(270370.25,510734.93) | 12130.64(8687.75,16419.44) | 12649.75(8908.19,16935.81) | 0.22(0.15,0.29) |
| New Zealand | 81588.04(60120.44,105954.39) | 93559.95(68918.91,121801.64) | 9166.39(6748.10,11946.14) | 9141.25(6729.76,11914.36) | 0.01(-0.01,0.03) |
| Nicaragua | 188644.25(133881.55,260160.19) | 243137.92(173472.19,334179.82) | 12429.41(8861.19,17091.76) | 12307.82(8771.53,16928.64) | -0.03(-0.03,-0.03) |
| Niger | 313261.83(219003.19,427754.02) | 1071969.16(750826.93,1460771.04) | 10920.49(7673.46,14823.24) | 10875.89(7642.13,14763.59) | -0.02(-0.02,-0.02) |
| Nigeria | 3585560.60(2664115.07,4668512.17) | 9677053.83(7149338.19,12708844.63) | 11338.17(8436.39,14746.92) | 10932.21(8085.38,14342.36) | -0.12(-0.15,-0.10) |
| Niue | 70.36(49.50,96.00) | 41.67(29.49,56.53) | 9621.27(6788.57,13094.07) | 9603.60(6776.05,13068.37) | -0.02(-0.03,-0.00) |
| North Macedonia | 49273.33(34660.32,65931.25) | 33036.18(23262.25,44121.98) | 9124.44(6411.67,12225.74) | 9099.79(6394.01,12193.87) | -0.01(-0.01,-0.01) |
| Northern Mariana Islands | 1201.02(850.81,1627.96) | 1191.97(842.29,1620.97) | 9913.06(6995.39,13492.47) | 9698.11(6844.33,13192.79) | -0.07(-0.10,-0.03) |
| Norway | 100306.08(75509.31,129637.63) | 134890.75(100775.37,172588.64) | 11014.89(8274.74,14282.65) | 13468.78(10051.54,17254.07) | 1.25(0.99,1.51) |
| Oman | 74160.05(52357.79,101728.70) | 119017.40(84041.22,162933.79) | 12003.60(8529.15,16363.21) | 12084.42(8577.09,16459.69) | 0.00(-0.01,0.01) |
| Pakistan | 4313190.18(3190506.42,5645337.19) | 8353005.00(6181767.51,10913960.62) | 10617.66(7861.14,13867.20) | 10607.58(7851.50,13852.14) | -0.03(-0.05,-0.02) |
| Palau | 479.17(339.23,649.93) | 357.88(253.26,485.90) | 9712.19(6854.08,13213.88) | 9713.55(6855.22,13216.31) | -0.02(-0.03,-0.01) |
| Palestine | 92777.91(65694.39,126672.54) | 216458.66(153573.19,295048.05) | 12074.90(8571.05,16448.16) | 12075.11(8571.36,16448.49) | 0.00(0.00,0.00) |
| Panama | 99993.06(71335.57,137443.46) | 140694.55(100310.13,193466.63) | 12350.19(8802.52,16985.90) | 12309.09(8772.18,16930.77) | -0.01(-0.01,-0.01) |
| Papua New Guinea | 139496.80(98272.78,190076.44) | 317275.07(223522.76,432285.62) | 9683.46(6833.89,13172.20) | 9663.14(6819.29,13145.87) | -0.01(-0.01,-0.01) |
| Paraguay | 273952.53(201694.79,371140.78) | 403782.10(298518.09,545184.96) | 19568.80(14447.55,26448.38) | 19485.40(14381.18,26345.75) | -0.01(-0.01,-0.01) |
| Peru | 613741.15(449421.41,809381.76) | 774801.49(556270.16,1059699.85) | 7942.87(5817.74,10471.81) | 8288.92(5940.94,11352.96) | 0.15(0.08,0.22) |
| Philippines | 2479416.12(1817003.41,3234859.82) | 3764003.40(2759910.68,4906487.76) | 10984.92(8051.36,14325.42) | 10940.94(8018.37,14272.54) | -0.02(-0.02,-0.02) |
| Poland | 871970.42(650266.61,1153703.84) | 546422.76(407772.74,722468.60) | 9177.81(6843.83,12138.01) | 9170.01(6837.85,12127.61) | -0.00(-0.00,-0.00) |
| Portugal | 341463.42(240971.50,457832.05) | 212975.07(150284.93,285218.75) | 13017.44(9160.32,17531.71) | 12992.39(9141.31,17499.55) | -0.01(-0.01,-0.00) |
| Puerto Rico | 136146.13(96412.07,183695.34) | 77944.40(55405.17,104782.63) | 12892.81(9111.53,17428.72) | 12876.72(9100.40,17404.76) | -0.00(-0.01,-0.00) |
| Qatar | 11694.26(8287.41,15995.13) | 46561.81(32878.62,63765.60) | 11962.24(8503.54,16313.68) | 12079.47(8573.66,16453.76) | -0.02(-0.04,0.00) |
| Republic of Korea | 1185232.46(831285.49,1609143.47) | 596380.48(419589.00,804324.37) | 8745.05(6105.69,11929.32) | 8303.20(5828.06,11232.71) | -0.25(-0.31,-0.20) |
| Republic of Moldova | 95840.97(67557.86,129722.49) | 46904.17(33115.34,63369.63) | 8428.92(5945.06,11400.20) | 8399.27(5922.71,11358.85) | -0.01(-0.01,-0.01) |
| Romania | 558246.82(393209.86,746348.08) | 297337.88(209338.28,397681.48) | 9131.18(6416.16,12234.33) | 9111.56(6402.50,12209.24) | -0.01(-0.01,-0.01) |
| Russian Federation | 3027058.36(2231167.89,3901993.09) | 2317400.96(1707240.82,2991159.62) | 8989.29(6625.19,11591.76) | 8974.13(6613.86,11571.95) | -0.00(-0.01,0.01) |
| Rwanda | 155120.85(107764.85,209993.18) | 281307.82(196236.78,379127.57) | 5964.22(4159.12,8042.83) | 5949.28(4148.28,8021.95) | -0.02(-0.04,-0.01) |
| Saint Kitts and Nevis | 1781.50(1259.28,2408.36) | 1541.25(1095.70,2072.52) | 12935.68(9141.54,17491.43) | 12940.97(9145.08,17499.52) | 0.00(0.00,0.01) |
| Saint Lucia | 6418.36(4536.38,8679.42) | 4574.38(3249.37,6152.15) | 12971.80(9166.25,17545.02) | 12848.32(9080.59,17362.60) | -0.04(-0.04,-0.04) |
| Saint Vincent and the Grenadines | 5336.38(3771.70,7213.52) | 3527.81(2497.93,4759.46) | 12908.16(9122.19,17450.87) | 12865.54(9092.37,17388.26) | -0.02(-0.02,-0.02) |
| Samoa | 6421.06(4531.84,8736.72) | 6812.64(4801.13,9280.89) | 9625.81(6792.66,13095.35) | 9713.77(6855.38,13214.98) | 0.03(0.03,0.03) |
| San Marino | 706.32(498.94,943.99) | 712.34(502.51,954.40) | 13057.36(9190.53,17583.34) | 12936.36(9099.82,17421.15) | -0.04(-0.04,-0.04) |
| Sao Tome and Principe | 5094.01(3569.07,6948.04) | 8496.25(5973.86,11534.24) | 10840.11(7616.98,14720.83) | 10862.93(7633.06,14748.09) | 0.01(0.00,0.01) |
| Saudi Arabia | 655272.27(463793.57,879897.38) | 926624.05(659010.25,1239926.33) | 11740.21(8325.72,15738.30) | 11697.62(8302.69,15685.37) | -0.03(-0.04,-0.03) |
| Senegal | 304991.63(213578.73,415697.03) | 625731.67(439484.02,850982.19) | 10921.61(7674.11,14825.09) | 10771.72(7568.75,14636.62) | -0.05(-0.05,-0.05) |
| Serbia | 211497.60(148625.54,282484.11) | 149969.46(105568.99,199836.47) | 9144.54(6415.61,12236.16) | 9033.31(6334.79,12089.70) | -0.04(-0.04,-0.03) |
| Seychelles | 2607.98(1821.78,3529.36) | 2518.39(1757.93,3411.68) | 10970.62(7654.08,14868.52) | 10941.81(7633.50,14831.30) | -0.01(-0.02,-0.00) |
| Sierra Leone | 146118.67(102354.88,198961.52) | 345471.22(242641.39,469070.78) | 10874.77(7641.05,14763.07) | 10886.49(7649.49,14777.96) | -0.01(-0.01,-0.00) |
| Singapore | 43960.60(31995.26,58780.91) | 47726.95(33647.10,65265.74) | 5613.19(4070.92,7543.59) | 6484.89(4576.23,8867.77) | 0.55(0.43,0.66) |
| Slovakia | 126951.98(89306.06,169971.69) | 78136.28(54957.26,104640.87) | 9135.31(6418.94,12239.59) | 9115.90(6405.61,12214.90) | -0.01(-0.01,-0.01) |
| Slovenia | 41380.68(29127.25,55348.12) | 28121.54(19761.09,37716.46) | 9123.40(6410.67,12224.41) | 9107.93(6399.93,12204.53) | -0.01(-0.01,-0.00) |
| Solomon Islands | 12762.03(8990.33,17392.47) | 22588.55(15930.46,30749.95) | 9709.62(6852.50,13208.22) | 9693.20(6840.76,13186.39) | -0.00(-0.01,-0.00) |
| Somalia | 175992.97(122121.11,238558.97) | 487126.68(338712.92,658246.35) | 5842.87(4070.20,7880.83) | 5890.92(4105.18,7943.33) | 0.02(0.02,0.03) |
| South Africa | 1113777.08(822414.77,1444150.28) | 1310407.06(968520.26,1699383.58) | 8674.48(6404.85,11252.21) | 8621.17(6369.32,11185.46) | -0.02(-0.03,-0.02) |
| South Sudan | 130378.38(90730.93,176081.48) | 232685.65(162129.63,313954.41) | 5883.42(4099.89,7933.28) | 5891.78(4106.15,7943.76) | -0.00(-0.01,0.00) |
| Spain | 1343932.16(956129.55,1803513.78) | 988784.08(704216.92,1330336.15) | 13447.65(9527.68,18118.91) | 13449.98(9558.14,18126.50) | -0.02(-0.04,0.01) |
| Sri Lanka | 607620.51(424055.52,823277.57) | 614482.12(429611.07,830759.61) | 10959.33(7645.50,14854.03) | 10966.31(7650.55,14863.36) | -0.00(-0.01,0.00) |
| Sudan | 885768.37(627353.15,1208839.27) | 1900210.59(1348727.57,2589858.55) | 12087.47(8578.13,16463.10) | 12057.88(8560.66,16427.71) | -0.02(-0.02,-0.01) |
| Suriname | 16489.52(11665.74,22265.83) | 19129.53(13534.37,25821.47) | 12887.55(9107.68,17419.76) | 12825.32(9064.11,17329.72) | -0.02(-0.03,-0.02) |
| Sweden | 216309.06(162285.95,278004.90) | 245143.26(184247.66,318906.92) | 13045.33(9769.25,16825.22) | 12967.43(9737.66,16886.94) | -0.02(-0.03,-0.02) |
| Switzerland | 145175.77(102889.24,196964.97) | 157527.24(111825.88,215135.13) | 11626.56(8215.09,15847.60) | 11626.68(8243.83,15899.48) | -0.00(-0.01,0.01) |
| Syrian Arab Republic | 602138.01(426173.61,822550.51) | 613234.76(438065.83,829407.39) | 12081.64(8575.24,16456.30) | 12114.54(8594.88,16495.71) | 0.02(0.01,0.03) |
| Taiwan (Province of China) | 523045.80(384782.78,683690.62) | 283253.86(198225.85,384429.71) | 8906.43(6544.83,11650.05) | 8643.55(6031.58,11778.80) | -0.19(-0.24,-0.14) |
| Tajikistan | 170641.46(119542.19,231526.73) | 273266.40(191175.31,371172.56) | 9234.23(6482.45,12504.11) | 9171.00(6432.16,12420.93) | -0.02(-0.03,-0.02) |
| Thailand | 2440470.46(1764264.48,3229282.54) | 1415548.61(1007934.50,1893689.15) | 13228.07(9535.77,17547.12) | 12100.39(8582.02,16260.77) | -0.30(-0.39,-0.21) |
| Timor-Leste | 26899.29(18723.98,36560.80) | 56282.11(39331.01,76132.42) | 10823.47(7554.46,14669.90) | 10914.35(7615.28,14792.66) | 0.03(0.02,0.04) |
| Togo | 148829.06(104211.79,202938.32) | 315913.03(221662.83,430040.34) | 10845.45(7620.44,14726.50) | 10792.19(7583.09,14661.86) | -0.03(-0.04,-0.02) |
| Tokelau | 50.92(35.82,69.44) | 39.80(28.06,54.23) | 9671.96(6824.99,13159.40) | 9649.14(6809.18,13127.02) | -0.03(-0.05,-0.00) |
| Tonga | 3692.69(2605.24,5026.21) | 3365.44(2373.35,4580.95) | 9695.63(6842.40,13190.53) | 9700.42(6846.00,13195.50) | 0.00(-0.00,0.00) |
| Trinidad and Tobago | 48619.08(34313.99,65807.24) | 37529.35(26557.01,50669.09) | 12901.51(9117.46,17441.23) | 12886.30(9106.93,17419.19) | -0.01(-0.01,-0.00) |
| Tunisia | 351594.50(249453.44,479132.91) | 324469.22(230307.78,442213.81) | 12080.37(8574.45,16454.80) | 12052.73(8557.20,16420.96) | -0.01(-0.01,-0.00) |
| Turkey | 2338836.79(1683482.22,3145408.89) | 2305751.80(1664716.52,3072823.58) | 11643.79(8375.28,15667.51) | 11757.25(8475.68,15687.33) | 0.12(0.07,0.16) |
| Turkmenistan | 116734.24(81803.06,158296.79) | 129495.86(90773.01,175422.19) | 9203.67(6457.01,12467.10) | 9149.21(6413.97,12394.26) | -0.02(-0.03,-0.01) |
| Tuvalu | 257.20(181.14,350.33) | 359.23(253.93,487.90) | 9730.28(6867.74,13233.16) | 9649.83(6809.80,13128.03) | -0.02(-0.03,-0.01) |
| Uganda | 381724.95(265562.56,516123.22) | 1018404.92(708904.46,1375484.64) | 5963.42(4159.11,8042.00) | 5924.14(4130.10,7986.75) | -0.02(-0.03,-0.02) |
| Ukraine | 975568.15(720952.77,1274070.06) | 588989.27(435055.66,770352.00) | 8433.71(6227.13,11027.50) | 8399.67(6201.52,10984.26) | -0.01(-0.02,-0.01) |
| United Arab Emirates | 51157.17(36176.61,70231.61) | 146466.87(103760.07,200422.66) | 12008.09(8543.33,16401.50) | 12068.70(8569.08,16480.43) | 0.00(-0.02,0.03) |
| United Kingdom | 1449634.39(1083635.10,1885511.46) | 1545433.64(1145350.01,2007061.85) | 12496.00(9323.94,16313.61) | 12493.62(9251.46,16243.65) | -0.00(-0.01,0.00) |
| United Republic of Tanzania | 572782.83(405895.89,780941.10) | 1315739.20(914606.62,1795125.85) | 5882.36(4178.42,7997.39) | 6097.53(4242.91,8307.77) | 0.29(0.21,0.38) |
| United States of America | 6507632.08(4821225.26,8397591.00) | 7517350.32(5603578.94,9819109.10) | 11595.79(8588.79,14986.40) | 11210.16(8340.79,14670.28) | 0.04(-0.15,0.22) |
| United States Virgin Islands | 4072.37(2880.34,5502.18) | 1940.92(1375.26,2617.48) | 12932.36(9138.81,17487.31) | 12892.58(9112.84,17426.29) | -0.03(-0.04,-0.02) |
| Uruguay | 65120.79(45933.75,88632.22) | 58226.11(41108.65,79080.34) | 7873.06(5547.38,10727.44) | 7856.76(5535.61,10704.28) | -0.01(-0.01,-0.01) |
| Uzbekistan | 652154.59(457071.00,884534.27) | 776610.00(543735.44,1053858.91) | 9237.80(6485.19,12509.09) | 9177.75(6437.87,12429.21) | -0.02(-0.02,-0.02) |
| Vanuatu | 5181.03(3643.61,7070.71) | 9958.03(7017.52,13563.23) | 9717.63(6858.35,13217.69) | 9708.85(6851.87,13207.37) | 0.00(0.00,0.01) |
| Venezuela (Bolivarian Republic of) | 782605.09(562206.96,1041114.94) | 838215.77(594629.39,1141432.62) | 11963.31(8597.51,15909.92) | 12445.33(8816.58,16969.82) | 0.16(0.13,0.19) |
| Viet Nam | 2600455.85(1812661.81,3528252.87) | 2504178.79(1745326.06,3399071.93) | 10965.85(7651.60,14860.72) | 10895.25(7602.61,14766.62) | -0.02(-0.02,-0.02) |
| Yemen | 614558.94(433387.57,843384.02) | 1522254.86(1078782.64,2077341.90) | 12030.96(8543.36,16394.83) | 12077.74(8572.64,16451.59) | 0.02(0.02,0.02) |
| Zambia | 208695.99(145730.67,284774.72) | 487078.06(340487.64,665476.20) | 6631.81(4634.88,9038.57) | 6623.95(4637.01,9033.77) | -0.05(-0.07,-0.03) |
| Zimbabwe | 356358.96(247393.93,484669.40) | 490872.99(341086.90,667017.18) | 8654.10(6022.41,11740.59) | 8649.11(6018.66,11734.33) | -0.00(-0.01,-0.00) |

Abbreviations: ASR, age-standardized rate; EAPC, estimated annual percentage change; UI, uncertainty interval; CI, confidence interval
