# Supplementary material for: Rising trends in the burden of migraine among children and adolescents: a comprehensive analysis from 1990 to 2021 with future predictions
Source: Front Public Health. 2025 Oct 23;13:1634098. doi: 10.3389/fpubh.2025.1634098 (PMC12589008; doi:10.3389/fpubh.2025.1634098)
Supplement: Supplementary table S5 — Incidence of migraine in children and adolescents aged 5 to 19 years in 1990 and 2021 across 204 countries and territories, with EAPCs from 1990 to 2021. [file Table_5.docx]

Table S5.Incidence of migraine in children and adolescents aged 5 to 19 years in 1990 and 2021 across 204 countries and territories, with EAPCs from 1990 to 2021

| Location | Incidence | | | | |
| --- | --- | --- | --- | --- | --- |
|  | Number of cases(95% UI) | | ASR per 100,000 population (95% UI) | | EAPC(95% CI) |
|  | 1990 | 2021 | 1990 | 2021 | 1990-2021 |
| Afghanistan | 80047.75(52210.24,114978.66) | 256356.98(167926.82,367809.83) | 2088.13(1366.02,2995.43) | 2077.52(1359.59,2981.09) | -0.01(-0.01,-0.00) |
| Albania | 17337.58(10948.22,25232.10) | 7857.80(4930.70,11493.89) | 1668.00(1055.57,2423.86) | 1658.37(1049.52,2410.47) | -0.03(-0.04,-0.02) |
| Algeria | 204980.50(134479.26,293770.17) | 247705.21(163088.76,354400.84) | 2081.94(1362.23,2986.98) | 2080.63(1361.58,2985.17) | -0.00(-0.00,-0.00) |
| American Samoa | 294.11(186.37,429.65) | 283.66(180.41,413.63) | 1811.43(1148.69,2645.49) | 1816.63(1151.53,2653.48) | 0.01(0.01,0.01) |
| Andorra | 260.93(162.94,378.30) | 290.66(182.35,420.24) | 2408.07(1513.09,3480.42) | 2416.10(1518.07,3492.12) | -0.00(-0.01,0.01) |
| Angola | 60178.39(37712.21,87403.76) | 206628.57(129786.21,299825.44) | 1583.20(988.98,2303.03) | 1588.16(992.16,2310.98) | 0.00(-0.00,0.01) |
| Antigua and Barbuda | 390.94(256.96,561.01) | 388.62(254.71,558.05) | 2205.44(1451.57,3162.64) | 2196.47(1445.66,3149.24) | -0.03(-0.04,-0.02) |
| Argentina | 142843.09(90138.43,211951.57) | 162931.78(102357.30,240811.24) | 1484.37(934.78,2206.43) | 1512.60(952.72,2231.22) | 0.09(0.07,0.11) |
| Armenia | 15970.41(10034.77,23678.62) | 9736.88(6140.50,14404.59) | 1698.36(1068.10,2516.26) | 1681.37(1057.10,2492.51) | -0.05(-0.06,-0.04) |
| Australia | 68944.09(42893.58,100340.76) | 83380.51(52199.21,120866.89) | 1751.01(1095.45,2539.34) | 1749.23(1094.38,2536.80) | -0.00(-0.00,-0.00) |
| Austria | 32486.13(20452.82,47225.38) | 30186.36(19116.41,43747.56) | 2283.93(1454.43,3303.80) | 2277.84(1450.91,3295.30) | 0.03(0.01,0.06) |
| Azerbaijan | 37569.60(23555.82,55784.84) | 39184.74(24711.28,57973.01) | 1696.78(1067.26,2513.98) | 1679.02(1055.60,2489.17) | -0.05(-0.06,-0.04) |
| Bahamas | 1789.74(1174.70,2570.69) | 2039.91(1342.26,2919.52) | 2201.50(1449.00,3157.04) | 2207.85(1453.45,3166.15) | 0.01(0.01,0.01) |
| Bahrain | 2924.32(1922.39,4187.51) | 6296.28(4096.49,9071.89) | 2083.89(1363.27,2989.63) | 2065.18(1353.58,2964.16) | -0.03(-0.05,-0.02) |
| Bangladesh | 820602.49(529708.26,1189579.94) | 922905.94(594268.62,1341653.78) | 1977.14(1274.01,2871.42) | 1980.61(1276.30,2876.64) | 0.02(0.01,0.03) |
| Barbados | 1411.91(925.96,2027.65) | 1127.56(738.87,1618.75) | 2198.82(1446.97,3153.27) | 2191.40(1442.07,3142.27) | -0.01(-0.02,-0.01) |
| Belarus | 35905.96(22425.97,52177.55) | 23954.91(15047.20,34673.37) | 1542.48(965.28,2238.74) | 1535.71(961.08,2228.39) | -0.01(-0.02,-0.01) |
| Belgium | 50919.04(31586.12,74284.50) | 55425.23(34805.84,81152.85) | 2723.35(1704.18,3952.28) | 2826.35(1774.99,4140.43) | 0.13(0.09,0.17) |
| Belize | 1626.94(1073.50,2330.29) | 2829.76(1855.06,4065.23) | 2202.69(1449.74,3158.91) | 2204.73(1451.19,3161.80) | 0.01(0.00,0.01) |
| Benin | 36442.96(23181.98,52290.49) | 100581.19(63817.59,144531.19) | 1934.53(1223.78,2783.89) | 1942.30(1229.11,2794.90) | 0.02(0.02,0.02) |
| Bermuda | 250.62(163.93,360.80) | 194.14(127.66,278.18) | 2202.15(1449.30,3157.74) | 2198.72(1447.02,3152.92) | -0.01(-0.02,-0.00) |
| Bhutan | 4795.03(3085.41,6977.50) | 3815.65(2448.84,5562.24) | 1961.64(1264.49,2850.58) | 1980.97(1277.18,2876.96) | 0.03(0.02,0.03) |
| Bolivia (Plurinational State of) | 39000.84(25893.51,55276.50) | 54820.36(36227.05,77905.69) | 1646.40(1089.25,2338.02) | 1642.57(1086.80,2332.68) | -0.01(-0.01,-0.00) |
| Bosnia and Herzegovina | 18892.91(11907.95,27542.86) | 8610.25(5433.89,12540.79) | 1670.39(1057.36,2427.27) | 1668.90(1056.28,2425.16) | -0.01(-0.02,-0.01) |
| Botswana | 8409.27(5275.55,12210.92) | 10796.42(6736.44,15715.71) | 1591.01(993.96,2315.64) | 1580.91(987.45,2300.15) | -0.02(-0.02,-0.02) |
| Brazil | 1595486.04(1162897.89,2100470.79) | 1515049.16(1112736.51,1996771.09) | 3127.35(2275.23,4123.09) | 3267.32(2405.30,4299.35) | 0.33(0.19,0.48) |
| Brunei Darussalam | 1109.66(693.85,1638.03) | 1360.74(847.48,2015.07) | 1387.14(868.46,2045.79) | 1376.30(862.34,2029.15) | -0.04(-0.06,-0.02) |
| Bulgaria | 30800.46(19472.98,44809.46) | 16687.89(10581.69,24225.83) | 1670.81(1057.47,2427.96) | 1668.23(1055.93,2424.19) | -0.01(-0.01,-0.00) |
| Burkina Faso | 73712.40(46968.10,105669.50) | 169474.96(107509.48,243606.35) | 1939.78(1227.30,2791.25) | 1946.61(1231.87,2801.72) | 0.01(0.01,0.01) |
| Burundi | 22604.72(14202.87,33092.67) | 56169.03(35297.17,82215.23) | 1098.14(686.91,1611.29) | 1099.74(688.00,1613.35) | 0.01(-0.00,0.01) |
| Cabo Verde | 2622.41(1663.51,3768.19) | 2886.40(1824.43,4154.50) | 1942.52(1229.31,2794.63) | 1939.67(1227.64,2790.05) | -0.00(-0.01,-0.00) |
| Cambodia | 79031.61(50173.07,114232.49) | 99815.08(63059.80,144536.26) | 2054.44(1297.76,2975.65) | 2039.61(1288.10,2954.57) | -0.02(-0.03,-0.02) |
| Cameroon | 76241.90(48385.24,109538.61) | 234012.78(148559.25,336072.43) | 1939.11(1226.89,2790.41) | 1938.22(1226.59,2788.19) | -0.00(-0.00,-0.00) |
| Canada | 124711.17(79619.69,182283.37) | 136414.42(85940.03,197355.77) | 2158.83(1380.30,3154.03) | 2131.15(1342.60,3085.01) | -0.04(-0.04,-0.03) |
| Central African Republic | 15607.14(9763.84,22699.19) | 32552.72(20368.44,47330.03) | 1582.04(988.03,2302.45) | 1583.24(988.92,2304.09) | 0.00(0.00,0.01) |
| Chad | 44453.62(28230.95,63859.11) | 142254.41(90366.05,204278.25) | 1946.63(1231.90,2801.61) | 1942.52(1229.35,2794.97) | -0.01(-0.01,-0.01) |
| Chile | 57652.96(36063.87,84590.40) | 58812.24(36983.96,85913.30) | 1503.37(945.71,2198.89) | 1533.87(965.36,2239.97) | 0.10(0.08,0.13) |
| China | 4336527.29(2892592.80,6089657.56) | 3534800.91(2384719.05,4928043.05) | 1292.68(868.84,1805.00) | 1382.16(930.97,1928.15) | 0.25(0.20,0.31) |
| Colombia | 229304.05(152205.44,321792.79) | 230739.64(151846.50,328530.71) | 2119.51(1407.48,2973.31) | 2131.60(1409.90,3022.01) | 0.03(0.02,0.04) |
| Comoros | 1986.59(1246.61,2911.44) | 2548.68(1593.08,3743.06) | 1094.00(684.24,1605.98) | 1092.82(683.49,1604.46) | -0.00(-0.01,-0.00) |
| Congo | 14795.30(9261.62,21502.67) | 29495.62(18474.67,42854.20) | 1587.00(991.41,2308.96) | 1584.11(989.51,2305.21) | -0.01(-0.01,-0.01) |
| Cook Islands | 115.93(73.47,169.39) | 74.95(47.44,109.65) | 1815.39(1150.83,2651.68) | 1825.85(1156.59,2668.01) | 0.02(0.01,0.03) |
| Costa Rica | 21310.98(14023.63,30712.86) | 21913.14(14377.48,31628.61) | 2086.46(1370.60,3010.14) | 2090.03(1372.73,3015.30) | 0.01(0.01,0.01) |
| Croatia | 17200.78(10872.17,25024.33) | 10401.99(6483.63,15146.32) | 1670.46(1057.33,2427.29) | 1654.81(1031.96,2409.57) | -0.14(-0.22,-0.06) |
| Cuba | 56841.91(36681.04,82526.80) | 40056.30(26270.98,57534.50) | 2190.97(1441.67,3141.88) | 2187.45(1439.50,3136.35) | -0.01(-0.01,-0.01) |
| Cyprus | 4700.85(2952.80,6795.55) | 5063.92(3180.70,7320.42) | 2406.63(1512.50,3478.11) | 2406.49(1512.48,3477.87) | 0.01(0.01,0.02) |
| Czechia | 40617.53(25711.70,59049.18) | 27983.68(17794.62,40538.82) | 1671.31(1057.86,2428.59) | 1670.42(1057.34,2427.30) | -0.00(-0.00,-0.00) |
| Cote d'Ivoire | 89230.51(56603.85,128218.01) | 193868.48(123040.06,278345.42) | 1938.68(1226.71,2789.31) | 1929.81(1221.42,2774.61) | -0.02(-0.02,-0.02) |
| Democratic People's Republic of Korea | 80894.18(50165.03,119204.30) | 75076.91(46604.92,110518.89) | 1487.10(927.92,2182.83) | 1467.47(916.38,2151.09) | -0.03(-0.04,-0.02) |
| Democratic Republic of the Congo | 225847.34(141408.35,328213.41) | 542585.07(339844.35,788251.20) | 1582.42(988.47,2301.99) | 1579.63(986.65,2298.15) | -0.00(-0.01,-0.00) |
| Denmark | 20826.78(12899.47,30698.54) | 21493.14(13717.58,31441.99) | 2138.37(1333.62,3141.40) | 2162.23(1382.41,3160.66) | 0.04(0.03,0.05) |
| Djibouti | 1732.25(1082.50,2546.41) | 4109.17(2571.52,6039.32) | 1083.12(677.11,1591.91) | 1073.31(670.84,1578.47) | -0.05(-0.06,-0.04) |
| Dominica | 520.31(341.58,746.83) | 342.30(224.53,491.10) | 2180.28(1434.56,3126.21) | 2195.27(1444.82,3147.51) | 0.01(-0.01,0.02) |
| Dominican Republic | 55119.02(36232.69,79118.92) | 61931.71(40590.24,89039.68) | 2214.07(1457.74,3175.48) | 2197.28(1446.13,3150.68) | -0.02(-0.03,-0.02) |
| Ecuador | 66255.16(44760.91,91005.46) | 91751.94(60372.81,132356.87) | 1827.04(1232.54,2511.93) | 1853.52(1218.37,2676.51) | 0.07(0.06,0.08) |
| Egypt | 410356.57(268098.07,583799.98) | 790231.67(545832.45,1085610.92) | 2106.80(1373.90,3000.82) | 2336.72(1610.57,3215.92) | 0.47(0.37,0.56) |
| El Salvador | 41765.59(27515.49,60116.30) | 36893.73(24199.76,53288.44) | 2091.76(1373.45,3017.98) | 2081.78(1367.78,3003.55) | -0.02(-0.02,-0.01) |
| Equatorial Guinea | 2482.15(1554.52,3609.88) | 9009.49(5629.48,13112.43) | 1583.30(988.93,2305.45) | 1539.92(962.04,2242.02) | -0.11(-0.12,-0.10) |
| Eritrea | 14547.38(9125.43,21336.61) | 24959.75(15613.34,36657.79) | 1086.82(679.48,1596.71) | 1088.10(680.46,1598.29) | 0.01(0.00,0.01) |
| Estonia | 5210.30(3251.75,7575.32) | 3285.11(2067.45,4751.96) | 1539.44(963.69,2233.67) | 1537.96(962.46,2231.88) | -0.01(-0.01,-0.00) |
| Eswatini | 5264.79(3302.41,7645.87) | 6225.94(3890.14,9056.36) | 1589.13(992.68,2312.92) | 1574.16(983.00,2290.73) | -0.03(-0.04,-0.02) |
| Ethiopia | 189465.15(127572.89,264572.05) | 405192.08(273349.69,567697.77) | 966.55(648.18,1352.33) | 980.38(661.40,1373.69) | 0.06(0.05,0.07) |
| Fiji | 4814.48(3058.65,7021.23) | 4736.99(3008.10,6911.69) | 1820.04(1153.54,2658.89) | 1820.87(1154.11,2660.17) | 0.00(-0.00,0.00) |
| Finland | 23208.51(14578.26,33542.06) | 22061.72(13845.24,31897.37) | 2418.67(1519.59,3495.56) | 2418.13(1519.26,3494.82) | -0.00(-0.00,0.00) |
| France | 294116.49(184945.66,424362.26) | 290445.33(182134.30,419151.70) | 2405.68(1527.45,3459.21) | 2363.47(1487.88,3405.99) | -0.09(-0.11,-0.07) |
| Gabon | 5580.39(3493.74,8112.93) | 9809.42(6134.90,14271.24) | 1589.19(992.81,2312.93) | 1596.05(997.26,2323.28) | 0.02(0.02,0.03) |
| Gambia | 7319.71(4647.52,10514.89) | 17703.97(11221.40,25454.50) | 1944.46(1230.47,2798.19) | 1943.98(1230.29,2797.32) | 0.00(-0.00,0.00) |
| Georgia | 22602.80(14190.02,33522.40) | 11551.22(7287.95,17087.16) | 1700.65(1069.60,2519.47) | 1685.40(1059.94,2498.13) | -0.05(-0.06,-0.04) |
| Germany | 326490.93(203968.96,473226.41) | 306145.99(192158.53,444209.02) | 2545.22(1600.72,3678.50) | 2574.38(1624.01,3724.45) | 0.04(0.01,0.08) |
| Ghana | 109025.87(69181.44,156606.56) | 226270.11(143381.49,325249.62) | 1936.77(1225.65,2785.87) | 1937.11(1225.91,2786.35) | 0.00(0.00,0.01) |
| Greece | 56906.65(36409.63,82670.38) | 37358.66(23211.09,53591.52) | 2515.03(1615.97,3645.25) | 2493.07(1550.51,3575.63) | -0.03(-0.04,-0.03) |
| Greenland | 261.04(162.64,381.68) | 244.88(152.72,357.86) | 2137.44(1334.52,3122.00) | 2151.28(1342.46,3141.71) | 0.02(0.01,0.03) |
| Grenada | 668.09(440.58,956.96) | 496.42(325.09,713.28) | 2197.48(1446.32,3150.82) | 2185.80(1438.36,3133.86) | -0.04(-0.06,-0.02) |
| Guam | 686.65(433.17,1006.34) | 653.84(414.08,956.21) | 1817.34(1152.15,2654.58) | 1818.74(1152.90,2656.89) | -0.01(-0.01,-0.00) |
| Guatemala | 73231.01(48404.85,105219.14) | 104324.36(68211.04,150948.21) | 2104.61(1381.76,3036.70) | 2093.73(1374.79,3021.06) | -0.02(-0.02,-0.02) |
| Guinea | 41280.39(26238.43,59257.65) | 101554.27(64538.39,145806.17) | 1939.09(1226.83,2790.32) | 1941.75(1228.74,2794.24) | 0.00(-0.00,0.01) |
| Guinea-Bissau | 7800.25(4957.25,11199.54) | 15288.68(9708.06,21955.85) | 1945.47(1231.09,2799.68) | 1940.29(1227.90,2791.42) | -0.01(-0.01,-0.01) |
| Guyana | 5907.17(3887.18,8467.44) | 4489.13(2945.31,6450.23) | 2213.09(1456.91,3173.64) | 2199.39(1447.52,3153.89) | -0.02(-0.02,-0.01) |
| Haiti | 51502.25(34022.96,73729.56) | 88795.36(58401.20,127408.96) | 2220.66(1462.08,3184.72) | 2203.39(1450.28,3159.80) | -0.03(-0.03,-0.03) |
| Honduras | 40391.84(26658.71,58094.05) | 67200.71(44052.53,97064.86) | 2089.78(1372.31,3015.22) | 2088.36(1371.67,3013.01) | -0.00(-0.00,0.00) |
| Hungary | 38909.85(24728.15,56413.26) | 23793.06(15007.38,34669.19) | 1670.74(1057.51,2427.85) | 1669.30(1056.60,2425.70) | -0.01(-0.01,-0.00) |
| Iceland | 1533.11(959.65,2220.22) | 1638.26(1030.42,2365.82) | 2418.54(1519.55,3495.32) | 2419.68(1520.29,3497.23) | 0.00(0.00,0.00) |
| India | 5810082.86(3963684.52,8022382.13) | 7647830.20(5197423.44,10531551.79) | 1977.42(1347.28,2731.73) | 1962.64(1336.91,2697.51) | -0.06(-0.08,-0.03) |
| Indonesia | 1361937.83(914582.30,1875475.90) | 1429316.64(957267.15,1971641.84) | 2085.47(1399.12,2873.71) | 2080.25(1395.65,2866.37) | -0.01(-0.01,-0.01) |
| Iran (Islamic Republic of) | 489581.12(340957.65,669873.91) | 425744.50(298042.09,576061.33) | 2145.35(1493.25,2938.90) | 2150.58(1503.52,2913.85) | -0.09(-0.20,0.01) |
| Iraq | 149417.57(97950.56,214302.21) | 277287.60(181539.21,397715.04) | 2078.13(1360.29,2981.75) | 2078.66(1360.45,2982.54) | 0.00(0.00,0.00) |
| Ireland | 25259.44(15842.16,36538.35) | 24950.38(15698.17,36021.93) | 2415.95(1518.05,3491.78) | 2419.38(1520.03,3496.58) | 0.01(0.00,0.01) |
| Israel | 36025.53(22614.74,52093.13) | 59280.51(37257.49,85666.21) | 2414.33(1517.04,3489.45) | 2416.66(1518.43,3492.75) | 0.00(0.00,0.00) |
| Italy | 291978.99(196999.52,401153.36) | 223019.23(151438.56,305407.91) | 2651.61(1800.42,3636.03) | 2656.18(1805.85,3636.70) | 0.10(0.00,0.19) |
| Jamaica | 17876.50(11759.85,25640.44) | 13830.86(9064.01,19861.56) | 2205.48(1451.65,3162.95) | 2197.17(1445.99,3150.55) | -0.02(-0.02,-0.01) |
| Japan | 373907.76(250457.40,525066.03) | 233010.23(157445.73,328152.09) | 1364.52(918.95,1914.72) | 1377.13(932.81,1938.78) | 0.05(0.02,0.07) |
| Jordan | 30980.12(20249.75,44500.77) | 78963.54(51586.82,113351.22) | 2075.63(1359.04,2978.33) | 2077.41(1359.91,2980.78) | -0.01(-0.01,-0.00) |
| Kazakhstan | 81194.82(51101.14,120215.54) | 81335.27(51423.10,120076.87) | 1704.74(1072.12,2525.20) | 1701.11(1069.55,2520.17) | -0.01(-0.01,-0.01) |
| Kenya | 105347.07(70882.62,146441.86) | 209071.90(140387.60,291079.98) | 1116.14(749.07,1554.17) | 1112.50(746.69,1549.54) | -0.01(-0.01,-0.01) |
| Kiribati | 448.04(283.57,654.72) | 715.43(454.89,1042.91) | 1820.38(1153.62,2659.37) | 1822.98(1155.25,2663.52) | 0.00(0.00,0.01) |
| Kuwait | 10311.49(6657.95,14668.27) | 17514.61(11289.70,25049.85) | 2115.92(1359.49,3019.29) | 2097.72(1348.07,3006.51) | -0.03(-0.03,-0.02) |
| Kyrgyzstan | 24973.82(15709.76,36988.68) | 34456.81(21763.01,50906.96) | 1706.89(1073.36,2528.24) | 1701.91(1070.09,2521.29) | -0.01(-0.01,-0.01) |
| Lao People's Democratic Republic | 31852.22(20213.48,46041.27) | 44134.26(27797.33,64026.17) | 2051.22(1295.43,2971.52) | 2044.28(1291.16,2961.15) | -0.01(-0.01,-0.00) |
| Latvia | 8413.86(5231.42,12259.82) | 4522.32(2834.57,6555.86) | 1540.30(964.27,2234.73) | 1536.47(961.56,2229.32) | -0.01(-0.01,-0.01) |
| Lebanon | 19681.41(12867.83,28259.18) | 26149.25(17137.46,37498.08) | 2075.52(1358.16,2978.51) | 2071.10(1355.50,2972.63) | -0.01(-0.02,-0.01) |
| Lesotho | 9424.00(5933.23,13664.95) | 10075.54(6282.47,14670.26) | 1590.14(993.33,2315.78) | 1581.93(988.12,2301.47) | -0.01(-0.01,-0.01) |
| Liberia | 17484.54(11085.38,25136.23) | 39385.22(24997.03,56566.65) | 1942.80(1229.08,2796.11) | 1938.28(1226.65,2788.21) | -0.02(-0.02,-0.01) |
| Libya | 35158.97(23092.60,50347.98) | 34141.60(22214.38,49090.77) | 2090.36(1367.94,2999.64) | 2081.32(1362.01,2986.09) | -0.03(-0.03,-0.02) |
| Lithuania | 12032.82(7498.22,17592.02) | 5939.66(3716.41,8663.38) | 1472.98(922.90,2146.46) | 1468.93(920.21,2140.93) | -0.07(-0.10,-0.04) |
| Luxembourg | 1523.05(951.46,2212.89) | 2378.07(1489.96,3448.51) | 2332.11(1464.80,3377.58) | 2325.34(1460.24,3367.01) | -0.02(-0.04,-0.01) |
| Madagascar | 49855.42(31285.39,73054.18) | 119098.35(74647.46,174601.68) | 1095.53(685.21,1607.97) | 1097.17(686.33,1610.08) | 0.01(0.01,0.01) |
| Malawi | 40109.48(25156.83,58774.10) | 86877.45(54413.52,127391.43) | 1097.48(686.47,1610.41) | 1098.28(687.07,1611.40) | 0.00(-0.00,0.00) |
| Malaysia | 108123.09(68286.52,155958.90) | 146638.53(91241.67,211515.35) | 1818.99(1147.98,2625.29) | 1872.49(1168.63,2695.24) | 0.14(0.11,0.16) |
| Maldives | 1754.94(1112.36,2539.51) | 2008.87(1270.47,2907.10) | 2044.60(1291.23,2961.93) | 2032.68(1283.92,2944.15) | -0.03(-0.04,-0.03) |
| Mali | 62491.92(39730.66,89689.14) | 187364.42(118897.01,269158.87) | 1939.22(1226.94,2790.28) | 1939.41(1227.32,2790.04) | -0.00(-0.01,-0.00) |
| Malta | 2109.22(1325.81,3047.25) | 1493.92(937.15,2161.20) | 2410.91(1514.91,3484.39) | 2409.51(1514.41,3482.19) | 0.00(0.00,0.01) |
| Marshall Islands | 350.96(224.01,509.91) | 318.79(202.04,465.91) | 1820.05(1153.50,2658.85) | 1822.62(1155.15,2662.89) | 0.01(0.00,0.01) |
| Mauritania | 14805.58(9393.16,21274.85) | 32650.21(20730.60,46900.35) | 1940.50(1228.16,2791.88) | 1943.02(1229.68,2795.95) | 0.01(0.01,0.01) |
| Mauritius | 6740.22(4274.19,9732.33) | 4785.81(2989.99,6966.40) | 2045.59(1292.14,2962.76) | 2046.30(1292.54,2963.83) | 0.00(0.00,0.00) |
| Mexico | 633663.44(443531.13,863379.93) | 669067.89(461327.44,908021.39) | 2009.46(1406.71,2738.11) | 2026.51(1399.44,2746.33) | 0.04(0.03,0.05) |
| Micronesia (Federated States of) | 761.26(485.07,1107.80) | 585.51(370.51,856.70) | 1816.68(1151.50,2653.72) | 1818.18(1152.41,2655.97) | 0.01(0.00,0.01) |
| Monaco | 88.25(55.03,128.10) | 124.84(77.96,181.03) | 2403.19(1510.78,3472.50) | 2424.20(1523.16,3503.54) | 0.04(0.03,0.05) |
| Mongolia | 13574.99(8550.98,20086.64) | 15567.24(9847.01,22982.87) | 1706.36(1073.03,2527.51) | 1701.69(1069.98,2520.98) | -0.01(-0.01,-0.01) |
| Montenegro | 2711.43(1712.94,3945.94) | 1903.51(1200.44,2774.14) | 1670.02(1056.93,2426.92) | 1662.55(1052.19,2416.32) | -0.02(-0.02,-0.02) |
| Morocco | 186713.41(122314.53,267688.39) | 200230.76(130793.66,287475.18) | 2088.40(1366.45,2995.93) | 2081.10(1361.83,2985.82) | -0.01(-0.02,-0.01) |
| Mozambique | 56560.81(35566.46,82756.32) | 137722.44(86441.63,201711.16) | 1100.74(688.32,1615.02) | 1099.64(687.75,1613.42) | 0.00(0.00,0.01) |
| Myanmar | 289138.92(182754.11,418569.34) | 318914.18(200811.98,462553.18) | 2050.36(1295.04,2969.85) | 2046.38(1292.52,2964.20) | -0.01(-0.01,-0.01) |
| Namibia | 8489.45(5307.47,12347.72) | 12618.61(7891.61,18346.98) | 1586.71(991.20,2308.76) | 1585.55(990.48,2306.99) | -0.00(-0.00,-0.00) |
| Nauru | 64.58(40.96,94.26) | 67.78(42.97,98.99) | 1822.95(1155.29,2663.40) | 1815.33(1150.78,2651.61) | -0.01(-0.01,-0.01) |
| Nepal | 148788.38(95647.55,215737.59) | 192809.08(121652.07,277396.99) | 2092.85(1339.66,3042.28) | 2080.33(1320.09,2984.64) | -0.02(-0.03,-0.02) |
| Netherlands | 65735.10(40948.93,96437.40) | 67494.88(42341.75,98809.95) | 2274.50(1436.45,3321.02) | 2373.21(1498.27,3461.53) | 0.23(0.15,0.30) |
| New Zealand | 14979.59(9978.78,20838.29) | 17920.48(11994.91,24858.72) | 1794.57(1201.85,2490.07) | 1790.02(1199.12,2483.85) | 0.01(-0.01,0.03) |
| Nicaragua | 33924.78(22361.86,48831.13) | 40369.96(26512.98,58260.20) | 2096.43(1376.36,3024.98) | 2081.77(1367.94,3003.50) | -0.02(-0.02,-0.02) |
| Niger | 61674.70(39204.04,88570.97) | 203854.53(129416.84,292854.29) | 1948.69(1233.35,2805.02) | 1941.96(1228.95,2794.46) | -0.01(-0.01,-0.01) |
| Nigeria | 669807.04(458220.66,919559.01) | 1810015.93(1238465.50,2516135.39) | 2054.80(1404.86,2822.70) | 1994.25(1362.36,2775.10) | -0.10(-0.12,-0.08) |
| Niue | 13.81(8.77,20.13) | 7.46(4.72,10.92) | 1810.30(1148.20,2643.38) | 1806.02(1145.90,2636.74) | -0.01(-0.02,0.01) |
| North Macedonia | 8817.15(5574.24,12824.26) | 5770.48(3635.23,8415.38) | 1670.26(1057.13,2427.08) | 1666.56(1054.83,2421.86) | -0.01(-0.01,-0.01) |
| Northern Mariana Islands | 211.65(133.10,310.98) | 217.30(138.50,316.45) | 1846.99(1169.81,2699.99) | 1813.78(1149.95,2649.22) | -0.04(-0.05,-0.03) |
| Norway | 18153.37(12078.54,25347.51) | 24693.02(16847.60,33866.69) | 2143.99(1435.92,2981.28) | 2550.32(1740.15,3498.35) | 1.03(0.82,1.24) |
| Oman | 14324.88(9477.29,20457.16) | 22622.80(14909.31,32359.40) | 2072.44(1357.35,2974.01) | 2082.61(1362.90,2987.76) | 0.00(-0.00,0.01) |
| Pakistan | 824786.28(569183.70,1125324.46) | 1543661.85(1063807.82,2107381.46) | 1937.50(1333.93,2647.02) | 1938.95(1335.08,2648.67) | -0.02(-0.04,-0.01) |
| Palau | 85.59(54.06,125.39) | 64.07(40.66,93.60) | 1818.67(1152.78,2656.71) | 1818.44(1152.87,2656.44) | -0.02(-0.03,-0.01) |
| Palestine | 16829.61(11037.77,24131.19) | 37509.31(24607.53,53739.89) | 2080.30(1361.33,2984.75) | 2080.80(1361.74,2985.38) | 0.00(0.00,0.00) |
| Panama | 16660.16(10936.18,24046.28) | 23616.53(15519.20,34069.49) | 2087.51(1371.28,3011.72) | 2082.52(1368.50,3004.41) | -0.01(-0.01,-0.01) |
| Papua New Guinea | 26760.66(17000.49,39026.26) | 60905.18(38670.12,88854.09) | 1811.07(1148.49,2644.99) | 1809.34(1147.57,2642.26) | -0.00(-0.01,-0.00) |
| Paraguay | 47120.08(32645.94,65269.20) | 62292.79(42984.89,86398.72) | 3150.81(2177.14,4369.04) | 3142.56(2171.06,4357.89) | -0.01(-0.01,-0.01) |
| Peru | 106137.71(71416.93,146228.66) | 131237.52(85228.16,188542.74) | 1370.31(920.97,1889.12) | 1435.90(934.79,2060.55) | 0.16(0.09,0.22) |
| Philippines | 475456.97(319193.11,654822.31) | 701638.22(470610.48,967028.01) | 2086.47(1399.78,2874.87) | 2079.36(1395.24,2865.24) | -0.01(-0.02,-0.01) |
| Poland | 163262.64(111633.89,228669.37) | 100368.50(68653.38,140414.59) | 1708.90(1167.09,2395.47) | 1707.09(1165.88,2392.76) | -0.00(-0.00,-0.00) |
| Portugal | 58888.12(36868.97,85238.11) | 36385.02(22712.05,52758.14) | 2421.52(1521.45,3499.57) | 2418.43(1519.44,3495.27) | -0.00(-0.00,-0.00) |
| Puerto Rico | 22050.65(14508.37,31590.47) | 11646.30(7621.43,16727.93) | 2196.18(1445.38,3149.04) | 2193.80(1443.83,3145.46) | -0.00(-0.01,-0.00) |
| Qatar | 2137.79(1406.59,3062.16) | 8781.28(5804.39,12536.39) | 2066.10(1353.28,2965.70) | 2081.75(1362.16,2986.72) | -0.01(-0.03,0.00) |
| Republic of Korea | 208978.81(128017.82,311396.32) | 108153.37(66953.46,158710.98) | 1644.41(1013.25,2439.84) | 1567.99(972.73,2297.47) | -0.25(-0.31,-0.20) |
| Republic of Moldova | 17724.85(11102.88,25707.01) | 8381.23(5249.47,12159.36) | 1542.16(965.30,2237.90) | 1536.34(961.49,2229.32) | -0.01(-0.01,-0.01) |
| Romania | 97121.72(61548.00,141068.14) | 52270.51(33100.65,75956.61) | 1672.05(1058.26,2429.77) | 1668.90(1056.34,2425.14) | -0.01(-0.01,-0.01) |
| Russian Federation | 554026.62(376716.08,769234.31) | 432366.69(294685.25,599332.14) | 1653.08(1124.90,2293.81) | 1649.43(1122.51,2289.19) | -0.00(-0.01,0.01) |
| Rwanda | 30401.89(19079.43,44527.84) | 51451.32(32166.22,75533.34) | 1099.25(687.65,1612.66) | 1096.17(685.67,1608.81) | -0.02(-0.03,-0.01) |
| Saint Kitts and Nevis | 301.29(198.37,431.90) | 233.38(151.98,336.77) | 2203.09(1450.20,3159.17) | 2203.49(1450.33,3159.95) | 0.00(-0.00,0.00) |
| Saint Lucia | 1084.80(714.35,1554.81) | 704.12(459.98,1013.40) | 2209.05(1453.85,3168.11) | 2192.02(1442.67,3142.74) | -0.03(-0.04,-0.03) |
| Saint Vincent and the Grenadines | 902.63(594.82,1292.79) | 576.65(379.05,826.87) | 2200.89(1448.60,3155.88) | 2195.76(1445.04,3148.46) | -0.01(-0.02,-0.01) |
| Samoa | 1197.98(761.65,1747.09) | 1300.81(825.76,1897.91) | 1803.84(1144.60,2633.71) | 1817.35(1151.95,2654.72) | 0.02(0.02,0.02) |
| San Marino | 117.61(73.21,170.77) | 122.20(76.40,177.04) | 2426.83(1524.86,3507.00) | 2405.24(1511.88,3475.96) | -0.04(-0.04,-0.03) |
| Sao Tome and Principe | 964.73(614.49,1382.94) | 1499.57(951.12,2154.80) | 1939.80(1227.73,2790.17) | 1942.29(1229.25,2794.34) | 0.01(0.00,0.01) |
| Saudi Arabia | 118042.73(76722.49,167042.94) | 153856.54(99675.13,218469.83) | 2027.90(1317.16,2872.68) | 2018.78(1313.17,2858.55) | -0.03(-0.04,-0.03) |
| Senegal | 58233.55(36978.99,83669.45) | 113028.27(71674.09,162330.08) | 1951.02(1234.73,2808.31) | 1929.31(1221.14,2773.65) | -0.04(-0.05,-0.04) |
| Serbia | 37519.09(23739.88,54573.14) | 25244.21(15944.06,36768.82) | 1680.13(1063.61,2442.46) | 1653.09(1045.75,2403.51) | -0.06(-0.07,-0.06) |
| Seychelles | 475.61(299.79,689.48) | 464.83(293.66,672.95) | 2046.91(1292.80,2964.95) | 2044.21(1291.25,2960.72) | -0.00(-0.01,0.00) |
| Sierra Leone | 27723.22(17549.04,39889.34) | 62286.18(39387.27,89671.16) | 1942.70(1229.14,2795.79) | 1945.36(1231.08,2799.49) | -0.00(-0.01,0.00) |
| Singapore | 7925.56(5023.76,11601.28) | 9414.77(5804.74,13756.04) | 1093.51(700.70,1591.10) | 1261.88(780.83,1839.94) | 0.53(0.42,0.65) |
| Slovakia | 22706.25(14398.91,32958.53) | 14040.16(8901.30,20382.71) | 1672.75(1058.77,2430.67) | 1669.75(1056.89,2426.35) | -0.01(-0.01,-0.01) |
| Slovenia | 7301.89(4621.48,10613.66) | 5155.48(3275.06,7473.26) | 1670.47(1057.21,2427.51) | 1668.23(1055.92,2424.18) | -0.01(-0.01,-0.00) |
| Solomon Islands | 2447.92(1555.61,3568.93) | 4267.22(2707.96,6228.26) | 1815.54(1150.90,2651.92) | 1813.51(1149.81,2648.76) | -0.00(-0.00,-0.00) |
| Somalia | 34376.17(21649.77,50345.89) | 93352.63(58438.36,137076.21) | 1077.79(673.62,1584.64) | 1086.62(679.51,1596.37) | 0.02(0.02,0.03) |
| South Africa | 206288.72(138260.84,284188.82) | 242056.38(162399.29,333151.00) | 1619.38(1086.43,2229.52) | 1611.76(1081.53,2218.72) | -0.02(-0.02,-0.02) |
| South Sudan | 24566.79(15390.35,36064.33) | 42872.50(26880.68,62899.56) | 1084.87(678.30,1594.16) | 1086.23(679.15,1595.94) | -0.00(-0.01,0.00) |
| Spain | 227992.06(144065.27,328386.68) | 174613.27(110585.60,251077.31) | 2484.01(1575.22,3575.33) | 2483.26(1572.68,3572.97) | -0.02(-0.04,0.00) |
| Sri Lanka | 112408.86(71040.67,162710.99) | 109933.72(69262.18,159338.47) | 2046.20(1292.40,2963.75) | 2047.00(1292.95,2964.90) | -0.00(-0.00,0.00) |
| Sudan | 158609.01(104086.30,227268.62) | 327891.17(214972.56,469989.25) | 2081.53(1361.78,2986.51) | 2077.59(1359.64,2981.17) | -0.02(-0.02,-0.01) |
| Suriname | 2778.76(1823.25,3993.03) | 3165.67(2083.86,4535.00) | 2202.51(1449.69,3157.69) | 2186.78(1438.65,3136.20) | -0.03(-0.04,-0.03) |
| Sweden | 38469.11(26085.56,52765.82) | 45461.63(30973.08,62707.79) | 2479.21(1689.86,3394.32) | 2464.91(1679.92,3400.59) | -0.02(-0.03,-0.02) |
| Switzerland | 26000.32(16338.08,38117.49) | 29199.67(18164.21,42836.90) | 2203.62(1396.04,3214.29) | 2205.23(1375.20,3230.66) | 0.00(-0.00,0.00) |
| Syrian Arab Republic | 109368.52(71932.66,156542.84) | 90256.04(58472.83,129908.98) | 2081.75(1362.28,2986.65) | 2086.78(1365.18,2993.49) | 0.02(0.01,0.03) |
| Taiwan (Province of China) | 94021.37(61532.53,134249.68) | 50000.40(31098.59,73211.31) | 1634.23(1066.06,2337.34) | 1597.11(1002.95,2327.46) | -0.14(-0.17,-0.10) |
| Tajikistan | 32690.65(20559.28,48425.96) | 52671.72(33203.66,77928.66) | 1707.19(1073.32,2528.75) | 1696.51(1066.69,2513.71) | -0.02(-0.02,-0.02) |
| Thailand | 421162.09(265236.79,607312.75) | 241355.06(151697.07,352947.22) | 2386.86(1504.50,3439.91) | 2218.27(1400.18,3235.62) | -0.25(-0.32,-0.18) |
| Timor-Leste | 5315.59(3355.42,7713.41) | 10212.98(6429.37,14817.28) | 2023.53(1277.31,2932.27) | 2037.45(1286.67,2951.72) | 0.02(0.01,0.03) |
| Togo | 28480.28(18084.54,40898.65) | 58173.94(36913.72,83534.56) | 1939.70(1227.28,2790.65) | 1932.58(1223.09,2778.91) | -0.03(-0.03,-0.02) |
| Tokelau | 10.07(6.37,14.72) | 7.52(4.80,10.95) | 1814.59(1150.47,2650.15) | 1807.80(1146.75,2639.80) | -0.02(-0.03,-0.01) |
| Tonga | 693.13(440.19,1011.26) | 636.56(403.66,929.52) | 1814.87(1150.56,2650.84) | 1813.78(1149.95,2649.22) | -0.00(-0.00,0.00) |
| Trinidad and Tobago | 8543.94(5625.49,12254.83) | 6190.68(4073.92,8871.11) | 2200.16(1448.02,3154.89) | 2195.69(1445.00,3148.42) | -0.01(-0.01,-0.01) |
| Tunisia | 60989.32(39959.92,87443.57) | 56035.37(36715.22,80353.96) | 2081.71(1362.24,2986.61) | 2075.82(1358.36,2978.91) | -0.01(-0.01,-0.01) |
| Turkey | 397874.34(258910.83,566638.14) | 386792.76(250115.39,555321.92) | 2014.72(1309.56,2870.84) | 2027.55(1313.10,2909.38) | 0.08(0.05,0.11) |
| Turkmenistan | 22060.21(13875.13,32681.18) | 24044.65(15113.10,35642.58) | 1704.77(1072.18,2525.23) | 1695.02(1065.97,2511.62) | -0.02(-0.02,-0.01) |
| Tuvalu | 49.64(31.43,72.54) | 65.68(41.57,96.07) | 1814.57(1150.29,2650.64) | 1807.45(1146.55,2639.33) | -0.01(-0.01,-0.00) |
| Uganda | 73216.26(45900.09,107310.49) | 192146.52(120451.71,281810.61) | 1098.03(686.71,1611.29) | 1091.51(682.54,1602.86) | -0.02(-0.02,-0.02) |
| Ukraine | 176995.30(118294.10,246570.82) | 108331.55(72715.80,150263.29) | 1565.43(1047.84,2177.95) | 1559.02(1043.52,2169.18) | -0.01(-0.02,-0.01) |
| United Arab Emirates | 10053.85(6583.20,14448.82) | 26440.72(17316.18,37849.30) | 2072.25(1353.85,2976.49) | 2079.99(1358.64,2980.13) | 0.00(-0.02,0.02) |
| United Kingdom | 261666.91(173278.68,368426.80) | 288659.92(193178.19,400795.69) | 2378.74(1583.40,3339.02) | 2387.16(1597.58,3316.21) | 0.01(0.00,0.02) |
| United Republic of Tanzania | 108921.35(69649.91,160380.42) | 245836.90(154028.39,360773.15) | 1079.23(687.92,1593.57) | 1117.62(698.73,1642.65) | 0.28(0.20,0.36) |
| United States of America | 1228724.29(831502.29,1694987.87) | 1416713.91(955224.85,1969462.44) | 2254.89(1528.43,3106.89) | 2216.90(1496.09,3082.70) | 0.07(-0.08,0.22) |
| United States Virgin Islands | 674.81(444.65,966.20) | 309.55(203.77,443.10) | 2202.26(1449.25,3158.42) | 2186.72(1440.02,3133.32) | -0.03(-0.04,-0.02) |
| Uruguay | 12327.59(7757.66,18090.44) | 10800.00(6763.92,15907.28) | 1519.84(956.25,2230.81) | 1516.34(954.04,2225.38) | -0.01(-0.01,-0.01) |
| Uzbekistan | 124053.39(78028.11,183734.88) | 148195.54(93289.04,219447.03) | 1708.06(1073.88,2529.96) | 1696.72(1066.78,2513.98) | -0.02(-0.02,-0.02) |
| Vanuatu | 1018.36(647.53,1483.70) | 1904.11(1207.49,2779.79) | 1815.27(1150.73,2651.55) | 1815.95(1151.13,2652.52) | 0.00(0.00,0.01) |
| Venezuela (Bolivarian Republic of) | 133615.26(88619.67,186446.05) | 136786.36(90235.46,194148.82) | 2034.17(1346.91,2841.67) | 2098.54(1385.54,2979.58) | 0.13(0.11,0.16) |
| Viet Nam | 494379.24(312527.57,716032.67) | 477145.17(302044.68,690511.93) | 2044.42(1290.99,2961.87) | 2033.49(1284.12,2946.26) | -0.02(-0.02,-0.02) |
| Yemen | 119754.15(79251.67,170967.16) | 268444.78(176592.46,384038.80) | 2072.13(1355.73,2974.16) | 2080.86(1361.60,2985.53) | 0.02(0.01,0.02) |
| Zambia | 38294.26(23758.45,55687.56) | 90179.03(56003.50,131067.43) | 1199.37(742.43,1746.07) | 1197.59(741.31,1743.39) | -0.04(-0.06,-0.03) |
| Zimbabwe | 67566.02(42350.70,98135.80) | 91681.50(57423.67,133217.89) | 1586.91(991.31,2309.05) | 1585.55(990.42,2307.28) | -0.00(-0.00,-0.00) |

Abbreviations: ASR, age-standardized rate; EAPC, estimated annual percentage change; UI, uncertainty interval; CI, confidence interval
