# Supplementary material for: Rising trends in the burden of migraine among children and adolescents: a comprehensive analysis from 1990 to 2021 with future predictions
Source: Front Public Health. 2025 Oct 23;13:1634098. doi: 10.3389/fpubh.2025.1634098 (PMC12589008; doi:10.3389/fpubh.2025.1634098)
Supplement: Supplementary table S6 — DALYs of migraine in children and adolescents aged 5 to 19 years in 1990 and 2021 across 204 countries and territories, with EAPCs from 1990 to 2021. [file Table_6.docx]

Table S6. DALYs of migraine in children and adolescents aged 5 to 19 years in 1990 and 2021 across 204 countries and territories, with EAPCs from 1990 to 2021

| Location | DALYs | | | | |
| --- | --- | --- | --- | --- | --- |
|  | Number of cases(95% UI) | | ASR per 100,000 population (95% UI) | | EAPC(95% CI) |
|  | 1990 | 2021 | 1990 | 2021 | 1990-2021 |
| Afghanistan | 18159.60(1350.02,45675.58) | 54959.58(4024.61,139662.62) | 457.98(33.44,1153.14) | 460.82(34.28,1170.30) | 0.03(0.03,0.04) |
| Albania | 3467.89(294.38,8751.27) | 1678.65(153.91,4160.37) | 331.02(27.91,835.59) | 328.35(28.96,817.30) | -0.03(-0.04,-0.02) |
| Algeria | 43880.19(3098.88,110349.66) | 51663.24(3440.50,130983.74) | 464.00(33.45,1165.73) | 464.31(32.16,1175.40) | 0.01(0.00,0.01) |
| American Samoa | 57.08(3.22,143.76) | 57.37(3.16,144.05) | 356.27(20.14,896.62) | 357.01(19.63,896.73) | 0.02(0.01,0.03) |
| Andorra | 54.81(2.89,138.50) | 60.38(3.24,149.33) | 465.53(23.60,1180.56) | 464.82(24.28,1152.60) | -0.01(-0.02,-0.00) |
| Angola | 11277.50(844.57,27434.47) | 38366.58(2950.74,94588.73) | 311.81(23.95,756.25) | 315.33(25.21,773.96) | 0.03(0.02,0.04) |
| Antigua and Barbuda | 85.75(4.08,213.80) | 90.98(4.42,223.40) | 473.86(22.32,1182.03) | 470.07(21.96,1156.31) | -0.04(-0.05,-0.02) |
| Argentina | 26597.71(2224.23,65903.40) | 31117.96(2597.98,78765.17) | 277.64(23.35,687.81) | 281.23(23.14,713.33) | 0.07(0.05,0.09) |
| Armenia | 3059.87(235.50,7650.48) | 1879.45(146.24,4663.63) | 329.96(25.46,824.26) | 328.32(25.79,814.64) | -0.02(-0.04,-0.01) |
| Australia | 13400.11(1002.61,33523.61) | 15584.88(1086.33,39398.00) | 322.38(23.46,809.08) | 323.03(22.46,817.00) | -0.00(-0.01,0.00) |
| Austria | 6622.12(395.22,16559.44) | 6010.96(360.63,15064.39) | 435.08(24.80,1091.21) | 432.40(25.15,1085.20) | 0.03(0.01,0.06) |
| Azerbaijan | 7354.36(570.06,18537.53) | 7547.67(561.57,19127.37) | 330.44(25.38,832.98) | 327.73(24.68,830.33) | -0.03(-0.04,-0.02) |
| Bahamas | 397.91(19.49,983.35) | 489.57(23.77,1218.74) | 472.91(22.71,1170.80) | 473.35(22.12,1182.37) | 0.01(0.01,0.02) |
| Bahrain | 605.21(41.77,1501.08) | 1481.94(110.36,3736.68) | 466.00(33.53,1152.30) | 459.76(33.02,1161.88) | -0.03(-0.05,-0.01) |
| Bangladesh | 156138.85(7339.11,393371.94) | 193052.02(10634.54,482689.08) | 398.07(19.19,999.25) | 400.64(21.77,1003.29) | 0.05(0.03,0.06) |
| Barbados | 324.10(15.99,805.75) | 269.35(14.11,661.83) | 471.88(22.56,1176.48) | 470.15(23.51,1160.29) | -0.01(-0.01,-0.00) |
| Belarus | 7378.37(822.19,17854.32) | 4733.99(495.96,11680.71) | 314.49(34.83,761.61) | 312.03(33.24,767.95) | -0.00(-0.01,0.00) |
| Belgium | 10839.91(485.86,27182.98) | 11379.88(487.55,28729.51) | 544.51(23.56,1368.77) | 564.00(23.93,1424.88) | 0.12(0.09,0.16) |
| Belize | 334.90(15.38,841.11) | 653.43(31.62,1616.49) | 473.21(22.21,1187.52) | 471.34(21.98,1168.67) | 0.00(-0.00,0.01) |
| Benin | 6508.57(416.83,16539.60) | 19461.20(1209.01,48545.19) | 390.58(26.64,987.26) | 395.26(25.15,983.76) | 0.03(0.03,0.04) |
| Bermuda | 56.67(2.85,140.26) | 44.87(2.20,112.28) | 472.58(22.93,1172.27) | 473.35(22.64,1187.09) | 0.00(-0.01,0.01) |
| Bhutan | 974.91(48.04,2493.90) | 821.13(42.13,2039.10) | 391.79(19.10,1003.01) | 399.37(19.83,994.09) | 0.07(0.06,0.08) |
| Bolivia (Plurinational State of) | 7942.98(478.28,19885.54) | 11826.71(765.24,29950.58) | 349.66(21.54,873.50) | 349.17(22.42,884.95) | 0.00(-0.00,0.01) |
| Bosnia and Herzegovina | 3917.47(355.00,9871.76) | 1786.41(159.61,4440.82) | 330.45(29.19,834.48) | 330.38(28.93,823.02) | 0.00(-0.01,0.01) |
| Botswana | 1613.65(126.01,3961.66) | 2185.72(171.33,5378.98) | 314.77(25.03,771.74) | 313.56(24.38,772.55) | -0.01(-0.01,-0.00) |
| Brazil | 358140.22(11575.43,900497.03) | 359674.51(11334.94,899585.62) | 705.98(23.05,1774.92) | 741.92(22.79,1856.69) | 0.41(0.23,0.58) |
| Brunei Darussalam | 203.63(17.44,516.72) | 266.45(24.31,673.60) | 257.07(22.04,651.96) | 255.78(22.69,649.69) | -0.03(-0.05,-0.02) |
| Bulgaria | 6463.77(561.05,16286.02) | 3390.40(300.53,8450.47) | 331.18(28.15,837.23) | 329.85(29.08,823.43) | -0.00(-0.01,0.01) |
| Burkina Faso | 13466.09(863.00,34104.01) | 32954.99(2035.15,82367.53) | 390.42(26.43,984.20) | 396.68(25.04,990.50) | 0.06(0.05,0.07) |
| Burundi | 4117.14(464.95,10257.60) | 10735.14(1187.10,26756.22) | 217.08(25.37,536.87) | 219.25(24.77,543.59) | 0.04(0.04,0.05) |
| Cabo Verde | 506.00(31.05,1296.19) | 614.98(40.02,1565.01) | 396.29(24.91,1012.95) | 395.86(25.31,1008.89) | 0.01(0.01,0.02) |
| Cambodia | 14702.92(675.11,36725.71) | 19963.75(961.64,49775.95) | 406.32(19.23,1012.77) | 405.65(19.52,1011.62) | 0.01(0.01,0.02) |
| Cameroon | 14421.64(921.17,36042.40) | 46125.23(2830.25,116183.34) | 392.32(25.91,976.05) | 395.07(24.72,994.45) | 0.03(0.02,0.03) |
| Canada | 24006.73(1410.08,61033.46) | 25910.03(1553.21,64027.34) | 402.83(23.30,1025.24) | 394.89(23.48,977.48) | -0.06(-0.06,-0.05) |
| Central African Republic | 2886.69(225.51,7145.22) | 6341.17(508.42,15627.86) | 308.50(24.59,760.49) | 312.97(25.32,770.86) | 0.05(0.04,0.06) |
| Chad | 8309.79(513.13,20838.54) | 26899.35(1654.71,67008.00) | 393.28(25.23,984.15) | 395.12(25.26,980.90) | 0.00(-0.00,0.01) |
| Chile | 11133.82(979.00,28326.20) | 11181.59(969.67,28555.41) | 280.55(24.17,715.94) | 284.31(24.41,727.40) | 0.09(0.07,0.12) |
| China | 903071.93(76020.61,2207892.93) | 665235.30(49378.45,1613597.72) | 247.83(20.01,609.63) | 265.91(19.94,643.95) | 0.28(0.23,0.32) |
| Colombia | 50518.40(2388.96,127092.34) | 54854.99(2825.37,137739.47) | 462.70(21.79,1164.51) | 466.82(22.97,1174.84) | 0.05(0.03,0.06) |
| Comoros | 381.71(43.81,964.87) | 516.19(56.87,1259.63) | 217.56(25.39,548.75) | 218.04(23.89,532.77) | 0.02(0.01,0.02) |
| Congo | 2865.30(225.10,7027.20) | 5815.36(458.14,14445.98) | 313.47(24.91,768.35) | 313.99(24.91,779.76) | 0.01(0.00,0.01) |
| Cook Islands | 23.22(1.24,59.26) | 15.69(0.87,39.37) | 358.76(19.04,915.91) | 361.56(19.61,909.13) | 0.03(0.01,0.04) |
| Costa Rica | 4506.04(212.39,11365.04) | 5006.01(243.65,12686.01) | 454.94(21.81,1145.43) | 455.76(21.74,1157.02) | 0.02(0.01,0.03) |
| Croatia | 3544.82(303.34,8858.66) | 2158.36(198.83,5357.08) | 332.00(27.99,831.32) | 328.22(29.82,816.44) | 0.01(0.01,0.02) |
| Cuba | 14289.56(726.64,35592.65) | 9028.03(454.41,22429.81) | 469.36(21.76,1176.87) | 469.74(23.01,1168.96) | -0.13(-0.21,-0.06) |
| Cyprus | 919.06(45.99,2259.59) | 990.93(48.01,2494.00) | 466.71(23.27,1147.85) | 464.89(22.41,1170.66) | 0.00(-0.00,0.01) |
| Czechia | 8650.77(762.19,21628.52) | 5584.22(478.96,13909.29) | 331.31(28.51,831.59) | 331.50(28.63,826.84) | 0.01(0.00,0.02) |
| Cote d'Ivoire | 16636.47(1037.39,41246.69) | 37706.68(2308.81,94318.23) | 390.86(25.20,965.77) | 392.37(24.58,979.40) | 0.00(-0.00,0.01) |
| Democratic People's Republic of Korea | 16540.05(1109.17,40286.56) | 15728.11(1090.58,39254.07) | 293.63(19.26,716.48) | 286.98(19.23,718.27) | -0.05(-0.07,-0.04) |
| Democratic Republic of the Congo | 41602.89(3071.44,102568.09) | 104066.82(8339.25,256655.96) | 307.51(23.23,754.78) | 311.30(25.32,766.74) | 0.05(0.04,0.06) |
| Denmark | 4355.31(253.15,10916.50) | 4308.96(251.92,10865.94) | 406.21(22.55,1024.74) | 410.88(23.48,1039.98) | 0.04(0.03,0.06) |
| Djibouti | 348.69(38.95,857.99) | 813.86(94.85,2004.04) | 215.76(24.01,531.26) | 214.39(25.11,527.44) | -0.04(-0.05,-0.03) |
| Dominica | 116.13(5.39,291.42) | 80.72(4.13,201.85) | 467.62(21.27,1174.22) | 468.95(23.05,1176.37) | 0.00(-0.01,0.02) |
| Dominican Republic | 12138.05(577.44,30311.90) | 13818.83(635.59,34733.97) | 475.76(22.33,1189.08) | 471.57(21.21,1186.80) | -0.02(-0.02,-0.01) |
| Ecuador | 14306.27(830.15,35840.67) | 20372.99(1126.74,50660.03) | 396.85(23.19,993.83) | 405.60(22.40,1008.97) | 0.09(0.08,0.11) |
| Egypt | 89774.15(6207.40,222616.35) | 174196.57(10887.72,425448.94) | 469.54(32.89,1163.80) | 533.99(33.98,1302.67) | 0.59(0.47,0.70) |
| El Salvador | 9006.82(466.80,23047.36) | 8157.73(419.54,20672.91) | 452.36(23.70,1157.00) | 454.65(23.15,1153.14) | 0.01(0.00,0.02) |
| Equatorial Guinea | 458.75(36.98,1137.19) | 1809.48(144.32,4505.55) | 309.44(25.55,765.10) | 303.80(24.10,757.16) | -0.07(-0.07,-0.06) |
| Eritrea | 2742.76(311.56,6974.59) | 4925.31(560.30,12171.12) | 213.83(24.77,541.57) | 217.09(24.79,536.02) | 0.05(0.04,0.06) |
| Estonia | 1084.03(119.65,2629.84) | 673.38(75.37,1633.16) | 312.84(34.07,760.41) | 313.17(35.38,759.31) | 0.00(-0.00,0.01) |
| Eswatini | 999.20(74.94,2519.59) | 1238.61(100.53,3056.67) | 316.19(24.32,794.77) | 311.38(25.25,768.73) | -0.03(-0.04,-0.02) |
| Ethiopia | 33768.11(3785.62,85754.34) | 77831.35(9105.15,196367.97) | 184.89(21.42,465.47) | 187.17(21.86,472.38) | 0.05(0.04,0.06) |
| Fiji | 925.20(47.98,2345.81) | 923.50(51.33,2313.80) | 356.87(18.72,904.10) | 357.37(19.98,895.13) | 0.01(0.01,0.02) |
| Finland | 4564.01(225.95,11463.02) | 4435.71(226.66,11054.52) | 467.52(23.01,1175.07) | 467.10(23.50,1166.43) | -0.00(-0.01,0.00) |
| France | 60685.95(3497.70,151615.90) | 58836.95(3528.41,146299.03) | 465.90(25.88,1168.20) | 457.17(26.87,1139.34) | -0.09(-0.11,-0.06) |
| Gabon | 1059.04(78.35,2647.23) | 1940.56(150.09,4774.53) | 311.78(23.43,777.59) | 315.78(24.47,776.98) | 0.05(0.05,0.06) |
| Gambia | 1384.70(80.78,3495.41) | 3561.80(224.57,8963.97) | 394.05(23.75,991.93) | 394.78(25.04,993.18) | 0.01(0.00,0.01) |
| Georgia | 4498.94(343.49,11276.17) | 2168.36(153.50,5376.14) | 331.77(25.05,832.56) | 328.11(23.66,811.67) | -0.05(-0.06,-0.03) |
| Germany | 66186.62(3293.20,166155.36) | 61284.11(3131.41,154791.12) | 494.72(23.98,1244.09) | 498.18(24.96,1259.52) | 0.07(0.04,0.11) |
| Ghana | 20881.12(1319.40,53351.90) | 45020.46(2897.44,113489.19) | 392.48(25.44,1000.47) | 394.16(25.65,992.71) | 0.02(0.01,0.02) |
| Greece | 11819.31(588.44,29882.30) | 7659.50(403.41,19520.36) | 491.47(23.83,1244.40) | 481.36(24.78,1230.59) | -0.07(-0.08,-0.06) |
| Greenland | 47.28(2.84,117.53) | 46.07(2.73,115.11) | 395.41(23.83,981.78) | 398.69(23.48,996.90) | 0.04(0.03,0.06) |
| Grenada | 138.46(6.60,341.51) | 115.20(5.53,289.04) | 469.50(22.76,1156.98) | 465.72(21.50,1172.33) | -0.05(-0.07,-0.02) |
| Guam | 138.36(7.23,352.58) | 133.54(7.27,336.29) | 358.58(18.41,914.66) | 357.21(19.14,900.92) | -0.01(-0.02,-0.01) |
| Guatemala | 14320.10(690.43,36614.44) | 23480.89(1246.68,59381.69) | 453.21(23.30,1155.40) | 453.69(23.54,1149.43) | 0.01(0.00,0.01) |
| Guinea | 7569.13(444.90,19046.65) | 19491.09(1242.73,50111.71) | 391.54(24.08,982.29) | 394.36(25.99,1011.15) | 0.02(0.01,0.04) |
| Guinea-Bissau | 1466.01(86.72,3618.23) | 2976.54(195.04,7437.57) | 392.83(24.06,966.52) | 394.18(26.43,982.72) | 0.02(0.02,0.03) |
| Guyana | 1307.81(64.68,3270.10) | 986.72(48.27,2499.20) | 471.52(22.96,1180.50) | 469.91(22.54,1191.10) | -0.02(-0.02,-0.01) |
| Haiti | 10313.48(454.63,26049.92) | 18841.51(927.82,46733.10) | 469.24(21.29,1183.44) | 465.80(22.86,1155.60) | -0.04(-0.06,-0.03) |
| Honduras | 8168.58(386.92,20926.06) | 15177.47(800.02,38377.33) | 453.40(22.42,1157.08) | 453.25(23.42,1148.08) | 0.01(0.00,0.02) |
| Hungary | 8162.40(723.90,20150.65) | 4952.75(466.36,12256.17) | 330.00(28.91,818.09) | 330.43(30.38,819.91) | 0.01(-0.00,0.01) |
| Iceland | 305.21(15.72,765.01) | 325.55(16.57,814.74) | 466.68(23.66,1170.17) | 467.75(23.67,1172.21) | 0.00(-0.00,0.01) |
| India | 1094541.09(58252.06,2715028.90) | 1596330.60(91949.44,3936815.68) | 386.97(20.93,957.21) | 386.59(21.70,956.19) | -0.03(-0.06,-0.01) |
| Indonesia | 265644.81(13608.25,666456.71) | 290919.75(15213.09,729657.64) | 408.37(21.02,1024.67) | 407.90(20.95,1023.54) | -0.00(-0.00,0.00) |
| Iran (Islamic Republic of) | 99507.35(7210.32,245028.28) | 90233.06(6752.14,220177.38) | 469.83(35.19,1154.94) | 470.21(35.88,1146.27) | -0.12(-0.25,0.00) |
| Iraq | 31987.86(2223.56,80269.18) | 62598.12(4437.88,157251.59) | 462.23(32.71,1158.42) | 463.47(32.74,1164.38) | 0.01(0.01,0.02) |
| Ireland | 5050.26(253.79,12566.41) | 4898.33(233.61,12216.23) | 464.97(23.01,1158.98) | 464.92(22.11,1160.67) | -0.00(-0.01,0.00) |
| Israel | 7105.24(357.13,17868.61) | 11410.91(602.67,28316.27) | 469.35(23.43,1181.33) | 468.06(24.79,1161.12) | 0.01(0.00,0.01) |
| Italy | 63226.16(3127.16,157742.26) | 45268.54(2123.56,114361.93) | 509.75(23.77,1275.53) | 509.92(23.37,1290.15) | 0.08(-0.00,0.17) |
| Jamaica | 3901.31(186.45,9591.07) | 3248.60(161.20,8161.08) | 473.36(22.46,1164.36) | 469.78(22.37,1185.05) | -0.02(-0.03,-0.01) |
| Japan | 75055.29(7568.57,192813.36) | 44816.29(4367.76,115260.57) | 250.08(24.28,649.57) | 251.94(24.04,650.71) | 0.04(0.02,0.06) |
| Jordan | 6892.99(490.47,17538.29) | 18700.91(1364.76,47170.74) | 462.57(32.79,1177.22) | 464.56(33.10,1173.03) | -0.00(-0.01,0.00) |
| Kazakhstan | 15740.95(1214.44,39115.83) | 15162.16(1141.37,37654.07) | 331.95(25.69,824.66) | 332.68(25.78,823.38) | 0.01(0.01,0.02) |
| Kenya | 19828.14(2314.82,49694.88) | 41930.77(4947.40,105207.27) | 219.67(26.17,548.21) | 219.08(25.74,550.33) | -0.00(-0.00,-0.00) |
| Kiribati | 84.15(4.66,209.80) | 137.98(7.53,346.92) | 355.64(19.91,884.87) | 358.08(19.78,900.13) | 0.03(0.02,0.03) |
| Kuwait | 2177.31(137.68,5363.15) | 3894.55(269.55,9624.38) | 473.57(31.06,1161.22) | 467.99(32.72,1155.72) | -0.03(-0.04,-0.03) |
| Kyrgyzstan | 4768.65(355.55,12013.21) | 6361.93(482.88,15777.79) | 332.74(25.01,837.48) | 331.59(25.88,821.17) | -0.00(-0.01,0.00) |
| Lao People's Democratic Republic | 6020.68(306.02,14953.76) | 8932.00(437.20,22706.58) | 407.43(21.27,1011.03) | 406.86(19.71,1034.36) | 0.02(0.01,0.02) |
| Latvia | 1769.99(189.94,4328.91) | 923.41(98.74,2260.02) | 314.71(33.00,771.45) | 313.05(33.55,766.19) | 0.00(-0.00,0.01) |
| Lebanon | 4366.53(319.54,10930.63) | 5841.06(412.67,14450.67) | 460.63(33.64,1153.18) | 460.20(32.56,1138.34) | -0.00(-0.01,0.00) |
| Lesotho | 1768.41(138.82,4338.81) | 2052.97(166.80,5071.70) | 317.61(25.83,777.63) | 312.55(25.04,773.51) | -0.03(-0.03,-0.02) |
| Liberia | 3205.91(199.60,8074.14) | 7832.89(491.02,19685.32) | 387.12(25.03,971.32) | 389.10(24.58,977.82) | 0.01(-0.00,0.02) |
| Libya | 7765.74(544.44,19589.65) | 8298.09(622.52,20762.44) | 466.20(33.11,1175.18) | 462.99(33.40,1160.45) | -0.02(-0.03,-0.02) |
| Lithuania | 2502.84(264.39,6163.20) | 1204.97(121.07,2954.97) | 294.88(30.46,727.70) | 294.37(29.37,722.15) | -0.07(-0.10,-0.04) |
| Luxembourg | 304.38(18.45,755.12) | 473.08(27.19,1171.71) | 448.91(26.57,1115.55) | 447.87(25.32,1110.88) | -0.01(-0.03,0.00) |
| Madagascar | 9378.47(1025.09,23545.81) | 23192.46(2491.67,57552.56) | 215.61(24.08,539.11) | 217.13(23.55,538.04) | 0.04(0.03,0.04) |
| Malawi | 7512.41(871.79,18652.32) | 17500.74(1962.59,43859.07) | 215.07(25.48,531.61) | 218.34(24.45,547.63) | 0.07(0.06,0.08) |
| Malaysia | 20906.62(1145.62,53306.03) | 30149.25(1568.66,76913.76) | 357.24(19.71,910.34) | 369.23(18.84,942.09) | 0.15(0.12,0.18) |
| Maldives | 327.25(15.75,821.56) | 399.43(18.67,1010.89) | 405.36(20.03,1016.29) | 403.99(18.94,1022.17) | -0.03(-0.04,-0.02) |
| Mali | 11479.53(683.19,28944.88) | 35923.26(2183.27,89841.41) | 390.61(24.30,983.06) | 392.69(24.53,979.67) | 0.03(0.02,0.04) |
| Malta | 416.03(21.27,1051.19) | 294.80(15.13,739.55) | 465.75(23.68,1178.10) | 466.62(23.66,1171.57) | 0.01(0.00,0.01) |
| Marshall Islands | 63.48(3.28,159.57) | 64.42(3.52,162.34) | 357.12(19.28,896.71) | 359.53(19.52,906.78) | 0.02(0.01,0.02) |
| Mauritania | 2838.75(176.10,7034.86) | 6447.31(399.72,16224.96) | 393.74(25.08,974.46) | 396.23(25.02,995.46) | 0.02(0.01,0.02) |
| Mauritius | 1363.33(68.02,3418.35) | 1053.57(52.53,2643.94) | 407.50(20.42,1021.99) | 407.35(19.38,1024.93) | 0.01(0.00,0.01) |
| Mexico | 137645.46(7763.45,343493.91) | 149811.86(8768.55,372578.31) | 429.28(24.09,1071.82) | 433.13(24.83,1078.77) | 0.05(0.04,0.06) |
| Micronesia (Federated States of) | 143.95(7.74,357.49) | 120.66(6.94,302.38) | 357.33(19.78,886.77) | 357.81(20.17,898.15) | 0.02(0.01,0.02) |
| Monaco | 18.19(0.95,45.90) | 25.22(1.24,64.72) | 465.16(23.33,1176.48) | 466.16(22.26,1197.49) | 0.03(0.02,0.04) |
| Mongolia | 2594.19(185.47,6498.33) | 2744.32(185.39,6921.86) | 331.51(23.92,829.53) | 330.55(23.40,827.68) | 0.01(0.00,0.01) |
| Montenegro | 552.39(48.21,1378.11) | 395.38(35.62,1002.32) | 330.92(28.52,826.68) | 329.08(28.99,836.37) | -0.01(-0.02,-0.01) |
| Morocco | 41190.22(2878.88,103235.03) | 45842.25(3311.08,115392.68) | 464.57(32.67,1163.67) | 463.43(33.02,1167.17) | -0.01(-0.01,-0.00) |
| Mozambique | 10424.69(1129.97,25498.15) | 25916.47(2947.19,64733.32) | 216.20(24.09,526.76) | 216.48(25.22,538.66) | 0.02(0.01,0.03) |
| Myanmar | 57384.08(2818.62,144026.48) | 65775.20(3102.33,164645.66) | 405.74(19.94,1018.37) | 408.13(18.99,1022.61) | 0.02(0.01,0.03) |
| Namibia | 1669.19(127.70,4132.19) | 2516.31(202.90,6233.12) | 315.25(24.24,779.93) | 313.90(25.32,778.15) | 0.01(0.00,0.02) |
| Nauru | 12.09(0.65,31.00) | 13.39(0.74,34.20) | 359.10(19.66,919.86) | 358.06(19.76,914.77) | -0.01(-0.01,0.00) |
| Nepal | 28570.84(1399.89,71812.93) | 41913.69(2092.30,105424.90) | 425.31(21.49,1066.53) | 426.99(20.71,1076.16) | 0.02(0.01,0.02) |
| Netherlands | 13794.01(717.49,34408.95) | 13721.17(626.26,34878.37) | 436.58(21.54,1094.68) | 452.62(20.03,1153.04) | 0.21(0.14,0.28) |
| New Zealand | 2892.35(226.46,7275.63) | 3319.59(256.43,8369.37) | 324.22(24.50,821.12) | 324.06(24.83,818.54) | 0.01(-0.00,0.03) |
| Nicaragua | 6898.77(342.08,17481.29) | 8938.76(454.22,22641.94) | 455.37(23.47,1149.91) | 452.26(22.78,1146.52) | -0.01(-0.02,-0.01) |
| Niger | 11276.06(680.64,28535.28) | 38909.86(2403.45,99210.56) | 394.63(25.02,995.38) | 395.78(25.30,1006.53) | 0.01(0.01,0.02) |
| Nigeria | 128281.03(8313.48,321936.38) | 349911.64(22625.31,883042.84) | 406.17(26.73,1017.40) | 395.76(25.98,997.22) | -0.09(-0.11,-0.06) |
| Niue | 2.59(0.15,6.45) | 1.54(0.08,3.90) | 354.86(20.93,882.02) | 354.16(19.11,898.00) | -0.02(-0.04,0.00) |
| North Macedonia | 1789.03(165.69,4397.91) | 1200.08(108.78,3001.83) | 330.95(30.36,814.40) | 329.84(29.24,827.16) | -0.00(-0.01,0.00) |
| Northern Mariana Islands | 44.37(2.51,112.36) | 43.95(2.40,112.45) | 365.63(20.04,929.04) | 357.32(19.50,914.65) | -0.07(-0.10,-0.03) |
| Norway | 3601.15(218.08,8750.62) | 4819.17(231.84,11941.23) | 394.51(22.91,964.72) | 480.77(22.83,1193.89) | 1.23(0.97,1.49) |
| Oman | 2838.64(186.68,7083.59) | 4567.38(323.59,11536.31) | 461.47(32.84,1147.61) | 465.37(35.01,1172.40) | 0.01(-0.00,0.02) |
| Pakistan | 153079.34(7431.70,383629.80) | 296626.02(13364.38,743631.38) | 377.58(18.75,943.56) | 376.85(17.10,944.27) | -0.04(-0.06,-0.03) |
| Palau | 17.71(1.04,44.01) | 13.22(0.74,33.09) | 358.37(20.47,892.24) | 358.28(19.92,898.34) | -0.02(-0.03,-0.01) |
| Palestine | 3550.62(246.29,9095.71) | 8314.07(585.19,20841.79) | 462.90(32.95,1184.22) | 463.96(32.91,1162.49) | 0.01(0.00,0.02) |
| Panama | 3672.27(181.24,9373.51) | 5185.49(267.46,13103.51) | 453.39(22.20,1157.97) | 453.57(23.31,1146.53) | -0.00(-0.01,0.00) |
| Papua New Guinea | 5081.59(279.39,12759.81) | 11629.23(637.29,28634.47) | 353.03(19.67,885.65) | 354.47(19.62,872.16) | 0.02(0.02,0.03) |
| Paraguay | 10123.96(287.51,25735.91) | 14905.71(448.82,37488.19) | 723.64(21.36,1837.67) | 718.99(21.22,1809.13) | -0.01(-0.01,-0.01) |
| Peru | 22019.99(1739.13,55849.84) | 27943.57(2139.86,70439.04) | 285.04(22.62,722.79) | 298.66(22.54,753.74) | 0.18(0.11,0.26) |
| Philippines | 91769.63(4635.93,231179.26) | 139858.84(7132.05,352174.64) | 406.71(20.66,1024.49) | 406.28(20.57,1023.39) | -0.00(-0.00,0.00) |
| Poland | 31606.30(2819.52,79109.15) | 19811.97(1811.83,49277.91) | 332.79(29.87,832.34) | 332.35(30.46,827.08) | -0.00(-0.00,0.00) |
| Portugal | 12248.17(618.69,30686.66) | 7637.46(401.43,19180.59) | 466.05(22.79,1172.35) | 465.03(23.50,1172.54) | -0.00(-0.01,0.01) |
| Puerto Rico | 4988.14(232.22,12532.75) | 2864.50(137.13,7147.25) | 471.81(21.57,1187.64) | 471.76(21.31,1182.31) | 0.01(0.00,0.01) |
| Qatar | 449.27(32.62,1130.47) | 1786.92(122.57,4508.87) | 460.57(34.56,1155.50) | 465.29(34.07,1169.29) | -0.02(-0.04,0.01) |
| Republic of Korea | 41999.82(3265.91,109342.92) | 21197.21(1650.60,54340.82) | 309.04(23.26,807.59) | 294.57(22.51,757.35) | -0.24(-0.29,-0.19) |
| Republic of Moldova | 3563.19(379.46,8775.63) | 1747.60(196.53,4262.63) | 313.57(33.57,771.57) | 312.61(34.93,763.82) | 0.00(-0.00,0.01) |
| Romania | 20329.21(1774.03,50782.09) | 10807.31(940.48,26933.45) | 331.85(28.51,831.10) | 330.66(28.39,825.44) | 0.00(-0.00,0.01) |
| Russian Federation | 113559.68(14065.07,278645.10) | 86834.97(10421.97,211249.73) | 337.09(41.58,827.82) | 336.66(40.83,817.18) | 0.01(0.00,0.03) |
| Rwanda | 5631.19(590.38,14288.04) | 10322.29(1190.84,25746.92) | 217.38(23.49,548.50) | 218.20(25.09,544.65) | 0.03(0.01,0.05) |
| Saint Kitts and Nevis | 64.95(3.02,161.24) | 56.52(2.90,140.79) | 471.55(21.86,1170.71) | 473.19(22.70,1184.56) | 0.02(0.01,0.02) |
| Saint Lucia | 233.04(11.35,579.11) | 166.83(8.20,411.66) | 470.89(22.91,1170.38) | 467.42(21.80,1158.77) | -0.03(-0.04,-0.03) |
| Saint Vincent and the Grenadines | 194.60(9.26,490.60) | 128.59(6.14,323.26) | 470.61(22.40,1186.67) | 468.45(21.95,1178.65) | -0.01(-0.02,-0.01) |
| Samoa | 236.03(12.85,589.07) | 250.28(13.79,636.26) | 353.79(19.31,883.23) | 357.09(19.87,907.19) | 0.03(0.02,0.04) |
| San Marino | 25.51(1.40,62.84) | 25.61(1.28,63.70) | 470.21(24.40,1163.07) | 464.16(22.41,1158.86) | -0.04(-0.05,-0.04) |
| Sao Tome and Principe | 185.36(11.71,466.49) | 310.46(19.24,772.46) | 395.41(26.01,992.31) | 396.75(24.59,987.91) | 0.02(0.02,0.03) |
| Saudi Arabia | 25181.59(1787.25,62881.59) | 35786.85(2755.02,87527.26) | 451.79(32.68,1127.41) | 451.04(33.86,1104.89) | -0.02(-0.03,-0.02) |
| Senegal | 10954.03(655.56,27181.45) | 22673.49(1390.60,57017.83) | 393.46(24.37,973.85) | 390.47(24.12,981.62) | -0.02(-0.03,-0.02) |
| Serbia | 7680.74(664.09,18951.47) | 5470.38(479.43,13428.24) | 331.59(28.30,819.64) | 328.32(27.97,809.90) | -0.02(-0.03,-0.01) |
| Seychelles | 97.31(4.65,242.68) | 93.85(4.71,238.40) | 409.06(19.36,1020.62) | 407.59(20.41,1035.75) | 0.00(-0.01,0.01) |
| Sierra Leone | 5247.99(326.72,13045.63) | 12535.52(791.72,31346.75) | 391.50(25.00,970.83) | 395.13(25.00,987.65) | 0.03(0.02,0.03) |
| Singapore | 1614.71(187.99,4083.81) | 1717.06(169.82,4320.84) | 205.09(22.85,521.84) | 233.38(22.97,586.74) | 0.50(0.40,0.60) |
| Slovakia | 4610.63(388.75,11459.64) | 2833.55(240.49,7032.00) | 331.47(27.85,825.48) | 330.37(27.96,820.87) | -0.00(-0.01,0.00) |
| Slovenia | 1506.48(136.20,3825.86) | 1019.42(89.40,2576.66) | 331.59(29.63,843.63) | 330.26(29.19,835.34) | -0.01(-0.01,-0.00) |
| Solomon Islands | 468.05(24.36,1173.52) | 830.58(47.47,2117.01) | 356.46(18.81,892.83) | 356.54(20.48,908.32) | 0.00(-0.00,0.01) |
| Somalia | 6374.03(704.87,15952.32) | 17759.56(1965.33,44704.99) | 212.53(24.39,528.48) | 215.25(24.17,540.18) | 0.04(0.04,0.05) |
| South Africa | 40381.08(3554.11,99758.60) | 47509.82(4190.92,116542.25) | 314.35(27.52,777.23) | 312.40(27.46,767.19) | -0.01(-0.02,-0.01) |
| South Sudan | 4737.44(570.87,11696.13) | 8498.79(957.54,20941.28) | 214.11(26.12,527.69) | 215.28(24.38,530.19) | 0.01(0.00,0.02) |
| Spain | 48589.68(2676.96,123221.40) | 35619.99(1981.70,89507.93) | 485.08(25.75,1234.54) | 483.95(26.55,1218.44) | -0.02(-0.04,0.01) |
| Sri Lanka | 22577.77(1122.58,56633.00) | 22908.04(1100.83,58144.96) | 407.10(20.22,1021.19) | 408.38(19.31,1037.57) | 0.01(0.01,0.02) |
| Sudan | 33759.49(2496.36,84666.58) | 72804.52(5309.50,183104.35) | 461.46(34.87,1156.87) | 462.05(33.87,1161.78) | -0.00(-0.01,0.00) |
| Suriname | 601.08(28.00,1515.31) | 695.14(32.72,1730.38) | 469.62(21.56,1184.86) | 465.73(21.73,1160.29) | -0.02(-0.03,-0.02) |
| Sweden | 7759.74(376.24,19361.62) | 8779.32(426.85,21786.98) | 467.24(21.83,1168.45) | 464.09(22.34,1153.85) | -0.04(-0.04,-0.03) |
| Switzerland | 5212.56(307.04,13153.67) | 5626.43(315.87,14102.73) | 416.91(23.67,1055.48) | 415.01(23.03,1041.38) | 0.00(-0.00,0.01) |
| Syrian Arab Republic | 23063.99(1552.89,58511.84) | 23500.72(1731.27,58970.71) | 463.66(32.28,1173.74) | 462.06(31.82,1163.98) | 0.00(-0.01,0.01) |
| Taiwan (Province of China) | 19204.26(1127.05,47481.59) | 10412.29(661.28,25994.90) | 326.80(19.16,808.80) | 317.12(19.68,793.21) | -0.18(-0.23,-0.13) |
| Tajikistan | 6141.07(452.47,15261.92) | 9852.29(732.24,24551.39) | 332.86(24.87,825.09) | 331.26(25.12,823.90) | -0.01(-0.02,-0.00) |
| Thailand | 90835.66(3824.52,227746.94) | 52715.58(2264.76,134459.84) | 491.76(20.35,1234.05) | 449.68(18.72,1148.72) | -0.30(-0.39,-0.21) |
| Timor-Leste | 992.42(48.87,2465.19) | 2099.25(102.20,5275.23) | 400.01(20.03,991.75) | 406.77(19.50,1023.21) | 0.07(0.06,0.08) |
| Togo | 5350.98(334.37,13336.53) | 11427.97(766.10,28886.04) | 391.13(25.24,972.72) | 390.91(26.65,987.33) | -0.02(-0.03,-0.01) |
| Tokelau | 1.87(0.10,4.71) | 1.47(0.08,3.67) | 356.54(19.49,894.74) | 355.88(20.30,889.97) | -0.02(-0.04,0.00) |
| Tonga | 136.17(7.11,338.05) | 123.97(6.86,317.19) | 357.57(18.75,887.49) | 357.46(19.87,914.21) | 0.00(-0.00,0.01) |
| Trinidad and Tobago | 1774.52(81.79,4420.92) | 1368.80(64.76,3422.65) | 471.23(21.99,1172.93) | 469.62(21.97,1175.23) | -0.00(-0.01,0.00) |
| Tunisia | 13507.51(987.46,34066.98) | 12411.53(882.73,31142.68) | 464.23(34.12,1170.32) | 461.12(32.94,1156.80) | -0.00(-0.01,0.00) |
| Turkey | 90127.83(6780.60,225054.57) | 89113.72(6848.16,216699.25) | 448.48(33.65,1120.16) | 453.96(34.42,1105.12) | 0.13(0.08,0.18) |
| Turkmenistan | 4194.11(298.41,10570.24) | 4675.61(347.95,11569.12) | 330.92(23.72,832.61) | 330.36(24.57,817.33) | -0.00(-0.01,0.00) |
| Tuvalu | 9.46(0.54,24.19) | 13.30(0.74,33.65) | 358.28(20.55,915.64) | 357.03(19.56,903.55) | 0.00(-0.01,0.01) |
| Uganda | 13835.39(1545.56,34349.32) | 37241.08(4236.79,94405.80) | 216.73(24.70,535.72) | 217.02(25.01,548.81) | 0.02(0.01,0.03) |
| Ukraine | 36411.01(4151.07,87966.54) | 21961.65(2489.67,53443.32) | 314.30(35.41,761.34) | 313.28(35.74,762.10) | -0.01(-0.01,-0.00) |
| United Arab Emirates | 1951.15(133.63,5014.75) | 5615.07(399.48,14347.19) | 460.22(33.47,1179.53) | 463.38(33.75,1182.15) | 0.03(-0.00,0.05) |
| United Kingdom | 51983.52(2911.90,126764.11) | 55258.18(2966.97,136613.60) | 447.44(24.22,1095.35) | 446.43(23.78,1105.49) | -0.00(-0.01,0.00) |
| United Republic of Tanzania | 20721.81(2364.48,51116.13) | 48364.41(5351.05,120705.70) | 213.30(24.81,524.68) | 224.44(25.09,559.23) | 0.35(0.27,0.43) |
| United States of America | 234578.49(14487.99,572681.27) | 268533.83(17412.84,659624.02) | 417.59(25.36,1022.17) | 399.90(25.36,987.99) | 0.01(-0.17,0.20) |
| United States Virgin Islands | 149.56(7.13,371.00) | 71.14(3.20,175.69) | 474.58(22.50,1178.55) | 471.82(20.77,1167.19) | -0.03(-0.04,-0.02) |
| Uruguay | 2348.49(202.45,5983.65) | 2098.81(173.34,5278.33) | 283.71(24.28,723.86) | 282.69(22.91,714.43) | -0.01(-0.01,-0.00) |
| Uzbekistan | 23429.56(1745.78,58473.93) | 27944.92(1936.30,69327.01) | 332.29(25.02,828.07) | 330.70(23.24,819.36) | -0.01(-0.01,-0.00) |
| Vanuatu | 190.01(9.84,476.26) | 365.17(20.36,921.98) | 357.05(18.90,893.30) | 356.28(20.04,898.79) | 0.01(0.00,0.02) |
| Venezuela (Bolivarian Republic of) | 28744.28(1479.91,72221.85) | 30831.78(1587.93,78143.16) | 439.44(22.78,1103.86) | 457.39(23.24,1160.01) | 0.16(0.14,0.19) |
| Viet Nam | 96624.25(4709.71,239584.09) | 93565.00(4375.40,236018.09) | 407.71(20.04,1010.28) | 407.33(19.25,1027.11) | 0.01(0.00,0.01) |
| Yemen | 23160.02(1568.68,58665.69) | 57532.45(3981.97,146837.02) | 455.92(33.55,1151.62) | 456.93(32.37,1163.91) | 0.02(0.02,0.03) |
| Zambia | 7584.49(871.84,18861.64) | 17790.08(2095.84,44289.49) | 241.30(27.98,599.15) | 242.40(28.90,602.33) | -0.01(-0.03,0.01) |
| Zimbabwe | 12913.82(966.69,31877.30) | 17873.83(1458.81,44534.24) | 314.13(23.94,773.66) | 315.22(26.03,784.10) | 0.00(-0.00,0.01) |

Abbreviations: ASR, age-standardized rate; EAPC, estimated annual percentage change; UI, uncertainty interval; CI, confidence interval
